# Identification of Pathways Mediating Growth Differentiation Factor5-Induced Tenogenic Differentiation in Human Bone Marrow Stromal Cells

**DOI:** 10.1371/journal.pone.0140869

**Published:** 2015-11-03

**Authors:** Sik-Loo Tan, Tunku Sara Ahmad, Wuey-Min Ng, Amir Abbas Azlina, Mahmood Merican Azhar, Lakshmi Selvaratnam, Tunku Kamarul

**Affiliations:** 1 Tissue Engineering Group (TEG), National Orthopaedic Centre of Excellence for Research and Learning (NOCERAL), Department of Orthopaedic Surgery, Faculty of Medicine, University of Malaya, Pantai Valley, Kuala Lumpur, 50603, Malaysia; 2 School of Medicine & Health Sciences, Monash University, Sunway Campus, Selangor, Malaysia; University of Maryland School of Medicine, UNITED STATES

## Abstract

To date, the molecular signalling mechanisms which regulate growth factors-induced MSCs tenogenic differentiation remain largely unknown. Therefore, a study to determine the global gene expression profile of tenogenic differentiation in human bone marrow stromal cells (hMSCs) using growth differentiation factor 5 (GDF5) was conducted. Microarray analyses were conducted on hMSCs cultures supplemented with 100 ng/ml of GDF5 and compared to undifferentiated hMSCs and adult tenocytes. Results of QuantiGene® Plex assay support the use and interpretation of the inferred gene expression profiles and pathways information. From the 27,216 genes assessed, 873 genes (3.21% of the overall human transcriptome) were significantly altered during the tenogenic differentiation process (corrected *p<0*.*05*). The genes identified as potentially associated with tenogenic differentiation were ARHGAP29, CCL2, integrin alpha 8 and neurofilament medium polypeptides. These genes, were mainly associated with cytoskeleton reorganization (stress fibers formation) signaling. Pathway analysis demonstrated the potential molecular pathways involved in tenogenic differentiation were: cytoskeleton reorganization related i.e. keratin filament signaling and activin A signaling; cell adhesion related i.e. chemokine and adhesion signaling; and extracellular matrix related i.e. arachidonic acid production signaling. Further investigation using atomic force microscopy and confocal laser scanning microscopy demonstrated apparent cytoskeleton reorganization in GDF5-induced hMSCs suggesting that cytoskeleton reorganization signaling is an important event involved in tenogenic differentiation. Besides, a reduced nucleostemin expression observed suggested a lower cell proliferation rate in hMSCs undergoing tenogenic differentiation. Understanding and elucidating the tenogenic differentiation signalling pathways are important for future optimization of tenogenic hMSCs for functional tendon cell-based therapy and tissue engineering.

## Introduction

Tendon degeneration and overuse in sports are common ailment encountered in the field of orthopaedic surgery. One of the major contributing risk factors to these conditions is the loss of fibroblast function with age. This affects the synthesis and organization of ECM proteins as well as matrix remodelling during tendon healing. Consequently, tendon exhibits poor regenerative capacity and heals with fibrous tissues which compromise their function. To date, tendon repair remains a great challenge to orthopaedic surgeons and an excellent functional repair is highly demanded. Current tendon tissue engineering research has been focused in the investigation of intrinsic and extrinsic factors that can induce bone marrow stromal cells (MSCs) into tenogenic lineage for use as an alternative cell source to replenish functional tendon cells at tendon injured site. In this regards, growth and differentiation factor 5 (GDF5) has been identified as one of the important factors in inducing tenogenic differentiation in MSCs [1–3]. It can be used to induce MSCs tenogenic differentiation by either direct supplementing the growth factor into the cell culture medium [1, 2] or via blending/coating it onto a scaffold where the MSCs were seeded [3]. These methods have successfully induced tenogenic differentiation in MSCs *in vitro* with the presence of GDF-5.

In previous studies, it was demonstrated that the use of GDF-5 resulted in the increase in candidate tenogenic associated markers expression of MSCs [1–3]. The implications of the findings were many folds. Among which, it is suggested that the use of GDF-5 results in an ever increasing tenogenic response correlating to an increase in dosing [1, 2]. Furthermore, that the potential of using pre-differentiated MSCs provides several benefits which includes avoiding ectopic tissue formation and higher cellular phenotypic expression [4]. However, despite the outcome being remarkably observed, the cellular events which initiate these changes remain largely unexplained. One of the difficulties in studying the molecular events in tenogenic differentiation is the lack of clearly defined tenogenic molecular markers. The molecular footprint of tendon progenitor cells through to differentiated cells has only started to emerge in recent years with the discovery of scleraxis (*Scx*) which expressed in tendons from the early progenitor stage to the formation of mature tendons [5].

The transcriptional control of *Scx* in MSCs and tenocytes is been suggested dependent on bone morphogenetic protein (BMP)-signalling and Smad 8 [6]. Briefly, BMP or GDF ligands bind to the plasma membrane spanning type II BMP serine/threonine kinase receptor (BMPR II) which in turn binds to intracellular type I receptor (ALK2) forming an active receptor complex. Smad 8 is phosphorylated by the activated receptor, bound to Smad4 and translocate into the nucleus where it regulates transcription of target genes, i.e. scleraxis (*Scx*).This basic helix-loop-helix transcription factor, *Scx*, subsequently drive expression of genes, i.e. candidate tenogenic associated markes, (tenomodulin (*Tnmd*) and type-I collagen (*Col-I*)). Nevertheless, the GDF5 initiated translocation of Smads into the nucleus has also been reported in the transcription of genes involved in chondrogenic [7, 8] and osteogenic differentiation [9, 10]. In contrast to chondrogenic and osteogenic differentiation, the transcriptomes involve in tenogenic differentiation, largely remain to be explored. Analysis and identification of pathways involved in tenogenic differentiation is crucial to understand how GDF5 mediate their pleiotropic effect. Thus, it may ultimately be possible to block or stimulate specific pathways, promoting “desirable” effect (tenogenic differentiation) of GDF5 while blocking “undesirable” effects (such as osteogenic and chondrogenic differentiation) for tendon related therapeutic purposes.

In order to allow better applications of tenogenic MSCs in tendon cell based therapy and tissue engineering, it is an urgent need to understand the pathways that governs initial commitment and further differentiation into tenogenic lineage by GDF5 induction. In this study, we compared the gene expression profiles of human MSCs (hMSCs) at day 4 and 10 of GDF5 induction to the untreated hMSC as well as primary tenocyte culture. Our data suggest a set of co-expressed genes which were up- or down- regulated in the GDF5-induced hMSCs and tenocytes. These genes were potentially associated with tenogenic differentiation. Atomic force microscopy and confocal laser scanning microscopy showed complementary findings that cytoskeleton reorganization is an important event during tenogenic differentiation. Understanding the transcriptional profiles behind the GDF5 induction may therefore generate control over the production of *in vitro* tenogenic cells for tendon regeneration.

## Materials and Methods

### Human bone marrow stromal cell (hMSC) culture

Ethics approval to conduct this study was granted by the University of Malaya Medical Centre (UMMC) Ethics Committee (Reference number: 602.22). Written informed consent was obtained from each donor. Human bone marrow was harvested from six adult donors (S1 Table) undergoing intramedullary nailing in UMMC. The mononuclear cells were isolated from the bone marrow suspension with Ficoll-Paque Premium (GE Healthcare, Sweden) gradient centrifugation method [11, 12] and were characterized as hMSCs via various tests including flow cytometry analysis for specific cell surface markers, cell morphology analysis and the ability to undergo tri-lineage differentiation, i.e. osteogenic, chondrogenic and adipogenic differentiation [12, 13].

### Primary native human tenocytes (hTeno) isolation and culture

Native human tenocytes were isolated and cultured from adult human hamstring tendons free of pathology (n = 6) obtained from donors who underwent ligamentous reconstruction of the knees and arthroplasty of the knees (S1 Table), as previously described [2]. These cells were used for comparisons in the subsequent experiment.

### GDF5-induced tenogenic differentiation in hMSCs

The hMSC primary cultures (at P2, n = 6) were seeded in standard T25 culture flasks and supplemented with 100 ng/ml of recombinant GDF5 (Abcam, UK) for tenogenic differentiation as previously described [2], for 4 and 10 days. The tenocyte primary cultures (n = 6) were seeded in similar density to that of hMSCs and were used as positive control. These cells were not supplemented with GDF5. Immunofluorescence staining for candidate tenogenic markers (scleraxis (SCX), collagen type I (COL-I), tenascin C (TNC) and tenomodulin (TNMD)) was conducted to confirm tenogenic phenotypic expression in GDF5-induced hMSCs (day 4 and 10), compared to control hMSCs and primary tenocytes, prior to global gene expression analysis. Cells were collected from: (Group 1) control (untreated) hMSCs, (Group 2) day-4 GDF5-induced hMSCs, (Group 3) day-10 GDF5-induced hMSCs, and (Group 4) human primary tenocytes cultures; for total RNA isolation and target preparation for microarray analysis (S1 Fig).

### Immunofluorescence staining

In brief, cells seeded on cover slips were fixed with ice cold acetone for five min. Then, the specimens were rinsed twice with stain buffer (BD Pharmingen™, USA) before being hybridized with primary antibodies (unconjugated mouse monoclonal or goat polyclonal antibodies; S2 Table) at 4°C, overnight in a humid chamber. After overnight hybridization, the specimens were washed twice with stain buffer before incubated with fluorescence-conjugated-secondary antibodies (fluorescein isothiocyanate (FITC)-conjugated anti-mouse IgG and Texas Red-conjugated anti-goat IgG) and counterstained with nucleus stain, for 30 min at room temperature. Then, the specimens were washed twice with stain buffer and subsequently mounted with fluoroGel mounting medium (GeneTex, Inc, Irvine, CA). Stained specimens were examined and captured with Leica TCS SPII confocal laser scanning microscopy system (CLSM,; Leica Microsystem, GmbH, Germany) with LAS AF Lite software (Leica Microsystems, Mannheim, Germany). Fluorescence images were captured with sequential scanning to avoid fluorescence signal cross-talk; and line averaging (8 lines) to enhance the quality of the image.

### cDNA microarray analysis

The samples collected for microarray analysis were used for total RNA isolation and target preparation for hybridization onto the human gene arrays. In brief, total RNA was extracted using AllPrep DNA/RNA/Protein Mini kit (Qiagen GmbH, Hilden, Germany) according to manufacturer’s instructions and treated with RNase-free DNase I (Qiagen GmbH, Hilden, Germany) and stored at -80°C until further prepared as targets for microarray analysis. The integrity of total RNA samples used for microarray analysis was determined by Agilent 2100 bioanalyzer and Agilent RNA 6000 Nano kit (Agilent Technologies, Germany). Only samples with RNA Integrity Number (RIN) value ≥ 7.0 were proceed for microarray analysis. The concentration of total RNA was determined by NanoDrop™ 2000c UV-Vis Spectrophotometer (Thermo Scientific, Wilmington, DE). Then, an aliquot of 200 ng of total RNA (in 5 μL of nuclease free water) was convert to cDNA and amplified with Applaus™ WT-Amp ST System (NuGEN Technologies, Inc, San Carlos, CA, USA), as described in NuGEN Ovation manual and Affymetrix GeneChip Expression Analysis Technical Manual. The MinElute reaction cleanup kit was used to purify the cDNA samples, prior to *in vitro* cDNA fragmentation and transcription with biotinylated nucleotide with Encore™ Biotin Module (NuGEN Technologies, Inc, San Carlos, CA, USA). Subsequently, the 2.5 μg of purified and labeled cDNA samples were hybridized onto Affymetrix GeneChip® Human Gene 1.0 ST Array (HuGene, Affymetrix Inc, Santa Clara, CA, USA) using GeneChip® hybridization wash and stain kit and GeneChip® hybridization oven 640 (Affymetrix Inc, Santa Clara, CA). Then, automated washing and staining of gene arrays were conducted with the fluidics station 450 and scanned with the GeneChip® Scanner 3000 7G (Affymetrix Inc, Santa Clara, CA) and Affymetrix GeneChip Command Console 3.2.4 to get the CEL intensities files. The Affymetrix Power Tools and DABG detection calls were used for data pre-processing and filtering prior to the differential expression analysis with Linear Models for Microarray Data (Limma) software package for R programming. The significant differentially expressed genes obtained by Limma analysis were used for further comparison to the gene list obtained from Liu at al. [14] and Mensen et al. [15], to exclude the genes previously reported as up-regulated in adipogenic, chongrogenic and osteogenic differentiation in hMSCs, to remove the non-specific genes or non-tenogenic related genes. Then, these significant differentially expressed genes (unmatched with the adipogenic, chondrogenic and osteogenic related genes) were used for signaling pathway analysis with GeneGo Metacore™ software (Thomson Reuters). All microarray data may be accessed through the NCBI GEO database (Superseries number: GSE55027).

The microarray data were then validated by QuantiGene® Plex 2.0 assay, atomic force microscopy (AFM) and confocal laser scanning microscopy (CLSM) imaging of cytoskeletal reorganization in GDF5-induced hMSCs.

### QuantiGene® Plex 2.0 Assay

QuantiGene® Plex 2.0 assay (Affymetrix, Santa Clara, CA) kit was used for confirmation of the microarray analysis for the candidate tenogenic and non-tenogenic markers expression. This assay determined the mRNA expression levels of 15 genes (12 targets and three housekeeping genes, as detailed in S3 Table). It was performed for: (i) control hMSCs, (ii) day 4 GDF5-induced hMSCs, (iii) day 10 GDF5-induced hMSCs and (iv) tenocytes; according to the manufacturer’s protocol. Luminescence was measured using a microtiter plate luminometer (Bio-Rad, Hercules, CA, USA). The samples’ background signals were determined in the absence of RNA samples and subtracted from signals obtained in the presence of RNA samples. The presence and absence call was determined by limit of detection (LOD) of the assay, where LOD = background + 3 x standard deviation of background. Prior to the calculation of gene expression fold change value, the expression value of each sample was calculated by normalizing the average background-subtracted signal of each sample to the geomean of the selected reference genes (which consist of TATA box binding protein (*Tbp*), hypoxanthine phophoribosyltransferase 1 (*Hprt1*) and phosphoglycerate kinase 1 (*Pgk1*) that represented low, medium and high abundant housekeeping genes, respectively). The gene expression fold change value, for instance fold change in sample X versus sample Y, was calculated with formula log_2_ fold changes = log_2_(expression value of X/expression value of Y). A gene is considered for fold change analysis if the signal in both sample X and sample Y passes the LOD.

### Atomic force microscopy (AFM) live cell imaging

For atomic force microscopy (AFM) live cell imaging analysis, hMSCs were seeded onto glass cover slip with and without GDF5 supplementation and human native tenocytes were seeded onto glass cover slip without GDF5. Prior to AFM imaging, cells were incubated with mild concentration of glutaraldehyde (0.5%) for 2 h at 37°C, to increase the stability of cell membrane and to prevent the lateral mobility of receptors. The cover slip was attached to a closed cell incubation sample plate (S2 Fig) for imaging in a fluidic environment. AFM imaging was conducted with an atomic force scanner (AFM5500, Agilent Technologies, Germany) mounted in an acoustic chamber (vibration free environment) to reduce the noise or turbulence from the surrounding environment. Cantilever used were sharpened microlever (Bruker, Italy) with silicon nitride probe (spring constant 0.0005 to 0.02; nominal value = 0.01) for soft sample such as culture cells. Atomic force images were acquired in AC mode for liquid imaging with harmonic frequency (tip resonance frequency) at ~3–5 V amplitude. Images were taken in cell culture medium at 37°C, with low scanning speed at ~0.3 Hz (or <0.5) and with at least 512x512 points/line resolution. Each sample was scanned for at least three times, and the best representative image was shown. During the entire experiment process, the cells were tightly adhered to the substrate (cover slip).

### Immunofluorescence staining for cytoskeletal reorganization in GDF5-induced hMSCs

Cytoskeleton reorganization imaging was conducted with the same protocol for immunofluorescence imaging as described in the earlier section, except the overnight hybridization was conducted with primary antibodies and fluorochrome conjugated phalloidin (Molecular Probe, USA) at 4°C.

## Results

### hMSCs isolation and differentiation into tenogenic lineage

**Fig 1:**
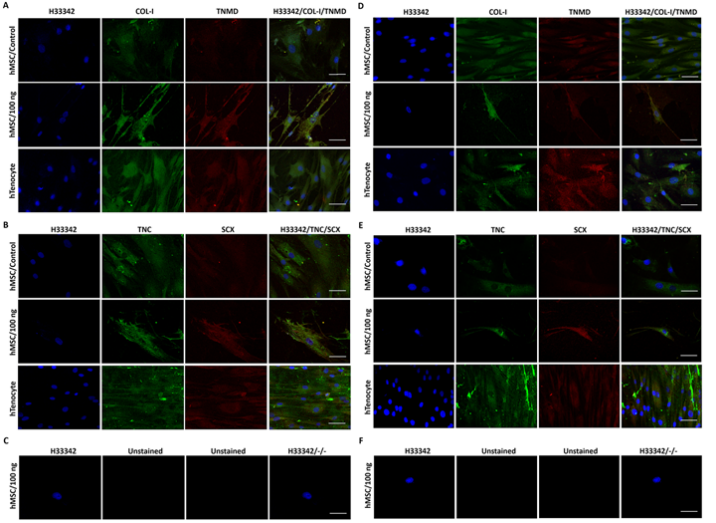
The candidate tenogenic markers (COL-I, TNMD, TNC and SCX) expression of GDF5 (100 ng/ml)-induced hMSC on day 4 (A, B, C) and day 10 (D, E, F) by immunofluorescence imaging. The extent of candidate tenogenic markers expressions were increased in GDF5 treated hMSC compared to the untreated control. An increase in the intensity of the expression of these markers was also observed in day 10 GDF5-induced hMSCs compared to that of day 4. Images were captured at 63X objective and a scale bar (50 μm) was depicted on the right bottom corner of the overlay images.

Bone marrow derived cells enriched for hMSCs (n = 6) were characterized to confirm their MSC phenotypic markers expression (CD29^+^, CD44^+^, CD73^+^, CD81^+^, CD90^+^, CD105^+^, CD166^+^, CD14^-^, CD19^-^, CD34^-^, CD45^-^, CD117^-^ and HLA-DR^-^) and capability for tri-lineage differentiation (osteogenic, chondrogenic and adipogenic differentiation) as previously described [12]. Tenogenic differentiation was conducted as previously described [2], and verified by immunofluorescence staining for candidate tenogenic markers. The results revealed an increase in candidate tenogenic markers protein expression in hMSCs in day 4 and day 10 GDF5-induced hMSCs compared to control (Fig 1); and considerable similarity in the cellular distribution of candidate tenogenic markers in GDF5-induced hMSCs and tenocytes at day 4 and 10. In addition, the day-10 GDF5-induced hMSCs showed an elongated “tendon cell-like” morphology.

### Quality assessment and normalization of microarray data

In this study, a total of 24 arrays were analysed, which consist of a sextuplicate (n = 6) of hMSC samples in each of the undifferentiated control (Group 1), and day 4 (Group 2) or day 10 (Group 3) GDF5-induced hMSCs, as well as a sextuplicate of tenocyte primary cultures (Group 4) (S1 Fig). Microarray data pre-processing analysis (S3 Fig) showed that the target prepared hybridized efficiently and specifically onto all arrays. The signals detected for the 24 arrays were comparable to one another and no outlier was detected. The total number of features detected was 33, 297. The robust multi-array averages (RMA expression values) were used to normalize the values in each group (based on the signal intensity values). The intensities that were below background signal, absent DABG (detected above background) detection calls were omitted.

**Fig 2:**
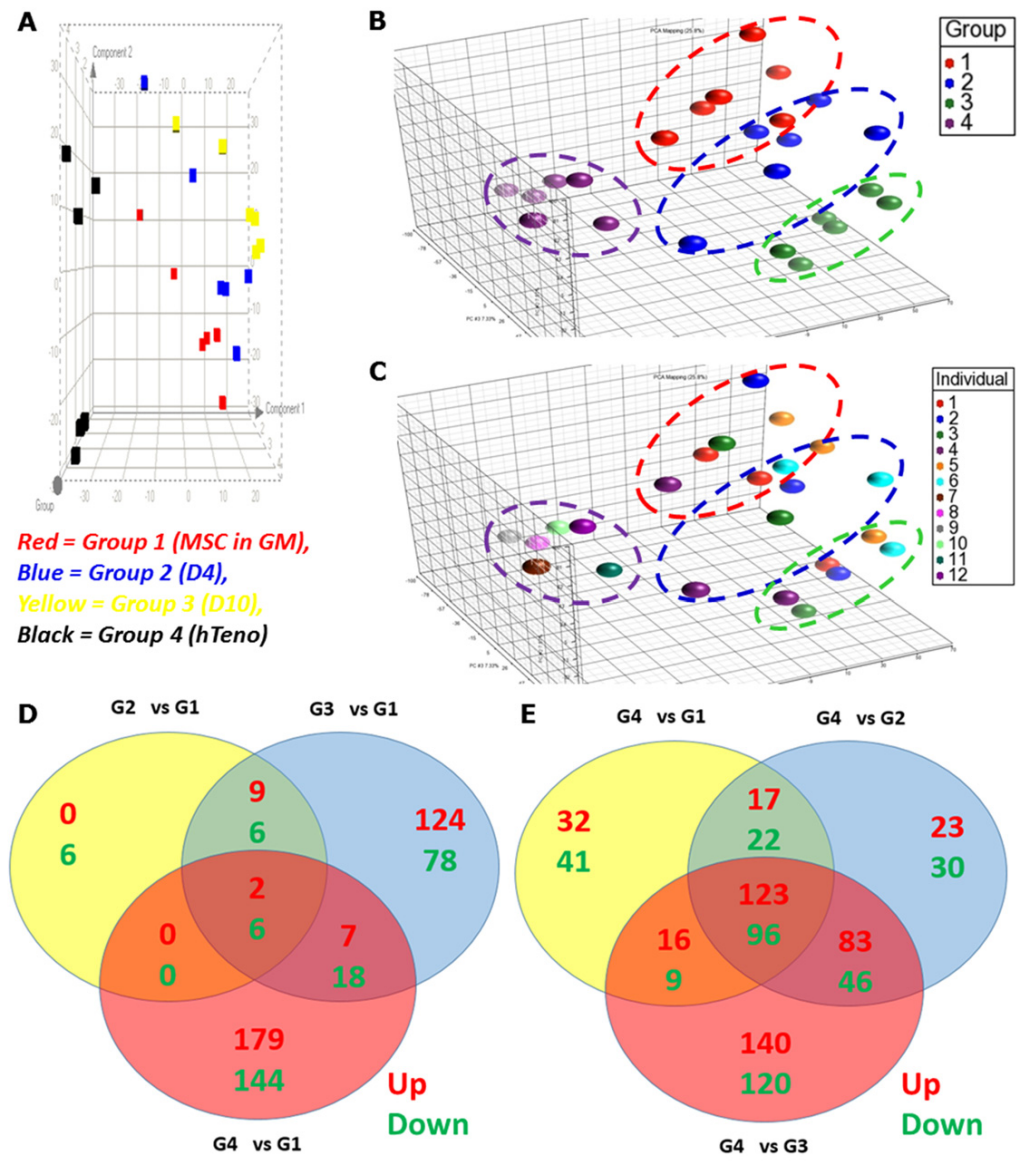
Overview of microarray analysis: principle component analysis (PCA) and Limma analysis. PCA analysis was performed on all samples and all probes to characterize the variability present in the data. The results showed a distinct separation between all the groups. The PCA was visualized in 2D view (A) and 3D view (B), with the different colour coded for different groups; and the 3D view (C) with the colour coded for different individual donor (In the legend, individual 1 to 6 were the bone marrow donors and individual 7 to 12 were the tendon donors). Image B and C showed that the arrays were grouped according to their experimental groups (treatment) but not according to the donor variation. (Group 1: Control hMSC, Group 2: Day-4 GDF5-induced hMSC, Group 3: Day-10 GDF5-induced hMSC, Group 4: tenocytes). The microarray experiments were designed to detect differential expression of transcripts with GDF5 treatment and were compared with Venn diagrams. The list of the significantly (corrected *p*-value) up- and down- regulated genes, were used to detect the altered candidate tenogenesis genes within the GDF5-treated groups (Group 2 and 3) as depicted in the intersections or uniqueness; between all comparisons with control hMSC (as depicted in D) and tenocytes compared to all the other groups (as depicted in E). The numbers in each section or intersections of the circles represented the total number of significantly differentially up- or down- regulated genes for the pairwise comparisons (as denoted above or below each circle). The numbers in green and red fonts indicated the significantly up- and down-regulated genes, respectively. (G1: Control hMSC; G2: Day-4 GDF5-induced hMSC; G3: Day-10 GDF5-induced hMSC; G4: tenocytes).

The heatmap of the RMA expression values showed the distance between all the arrays, and none of the arrays was detected as an outlier after normalization (S4 Fig). The dendrogram plots based on the genes those that were significant in at least one comparison (i.e. a set of 954 probe sets) showed that the arrays were clustered into different clades in the distance tree according to their tissue origin, one clade for bone marrows derived hMSC (either with or without GDF5 induction) and the other clade for tendon derived tenocytes (S4B and S4C Fig). Furthermore, the principle component analysis of all 24 arrays demonstrated that the hMSCs of all donors showed the same shift in accordance with GDF5 induction (Fig 2A–2C). This indicated that the discrimination of the arrays observed was not contributed by donor variations but the differences were due to the GDF5 supplementation and tissue origin of the cells (i.e. tenocytes and hMSC). Importantly, the Group 1 and 2 (control hMSCs and day-4 differentiated hMSCs) were most closely related to one another than the Group 3 and 4 (day 10 differentiated and tenocytes (mature cells) respectively). Following normalization, filtering and omitting the control probes, a total of 27, 216 probe sets was retained (S4 Table). These 27, 216 normalized intensity values of different groups were compared using the Limma package of Bioconductor [16] to detect the differential gene expression with the corrected *p*-values for multiple testing using Benjamini-Hochberg method [17].

### Confirmation by QuantiGene® Plex 2.0 assay

**Fig 3:**
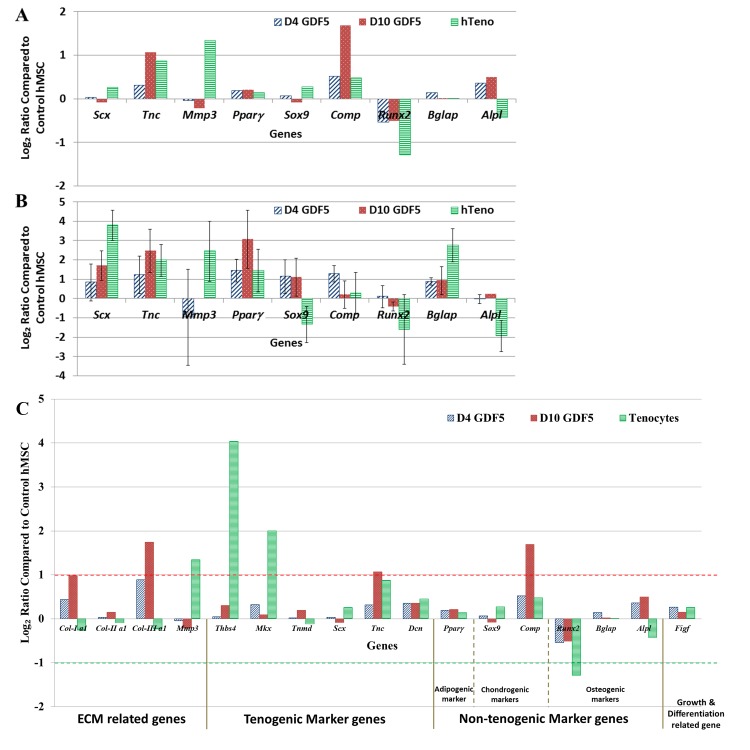
Expression levels of selected candidate tenogenic and non-tenogenic marker genes (total of nine genes) based on microarray and QuantiGene® Plex 2.0 Assay. (A) The graphical representation of genes expression patterns in hMSCs in response to GDF5 induction; with their respective log_2_ ratio based on microarray analysis. (B) Gene expression profiles independently validated using QuantiGene® Plex assay presented in log_2_ ratio. Expression variation for each gene was visualized with standard deviation. Considering that *Scx* and *Mmp3* were derived from three biological samples compared to that of microarray which was derived from six bioloigical samples, the variation in the expression profiles was not unexpected. Whereas the *Pparγ*, *Sox9*, *Comp* and *Bglap* were all weakly expressed genes (the non-tenogenic marker genes), thus the differences detected may result from the detected limit and sensitivity of the different platforms, which subsequently affected interplatform reproducibility of differentially expressed genes. (C) Expression levels of the ECM related, candidate tenogenic and non-tenogenic marker genes based on microarray analysis. The graphical representation of genes (n = 16) displaying changes in expression patterns in hMSC in response to GDF5 induction with their respective log_2_ ratio based on microarray analysis. The genes which showed at least fold change of 2 (log_2_ ratio = 1, red dotted line) and fold change of less than 0.5 (log_2_ ratio = -1, green dotted line) were regarded as significantly up- and down- regulated genes respectively.

To validate the data generated from cDNA microarray studies (Fig 3A), we performed QuantiGene® Plex assay on the same total RNA samples used in microarray studies. The average log ratio (log_2_ fold change) by QuantiGene® Plex assay was compared with average fold change by microarray detection. We selected genes indicative of different lineages, both candidate tenogenic and non-tenogenic markers, as shown in S3 Table: *ScxA*, *Tnc* and *Tnmd* as candidate tenogenic markers; *Pparγ* as adipogenic marker; *Sox9* and *Comp* as chondrogenic markers; *Runx2*, *Bglap* and *Alpl* as osteogenic markers. Among the 12 targets measured, three targets (*Col2a1*, *Figf* and *Tnmd*) were detected as absent calls in all the samples in the QuantiGene® Plex assay, hence were excluded from fold change analysis (Fig 3B). The rest of the other 9 targets were detected in all the samples (all the 6 samples in each group), except *Scx* and *Mmp3* were only detected in 3 samples among the 6 samples measured (Fig 3A and 3B).

Despite the fold change detected with QuantiGene® Plex assay was relatively higher compared to that of microarray analysis, the overall the gene expression profiles obtained were consistent in *Tnc*, *Mmp3*, *Runx2* and *Alpl*, but showed some differences in the expression profiles for *Scx*, *Pparγ*, *Sox9*, *Comp* and *Bglap* (Fig 3A and 3B). The genes found to be differentially expressed in the microarray analysis were confirmed to be differentially expressed by QuantiGene® assay (Fig 3A and 3B). However, the degree of increased or decreased expression differed for some genes, likely as a result of the difference in sensitivity of the two assays. Nevertheless, the result of this comparison gave us the confidence to proceed with data analysis, in particular analysis of biological pathways involved.

### Genes differentially regulated during tenogenic differentiation by GDF5 induction

The results of Limma package of Bioconductor analysis showed that the corrected *p*-value discovered a higher number of significant differentially expressed genes at *p*<0.05 than the uncorrected *p*-value at *p*<0.001 (Table 1; S5 Table), except for Group 2 vs 1. The corrected *p*-values provided a better control in the false discovery rate, thus the significant gene lists (of a total of 954 genes) obtained based on the corrected *p*-value were used for the subsequent analysis. The 954 genes were further compared to the gene list obtained from Liu at al. [14] and Mensen et al. [15] to exclude the genes previously reported as up-regulated in adipogenic, chongrogenic and osteogenic differentiation in hMSCs, to remove the non-specific genes or non-tenogenic related genes. Subsequently, we obtained a list of 873 genes, which was used for the following pathway analysis.

**Table 1:** A summary of the number of differentially expressed probe sets.

	Uncorrected *p*-value <0.001		Corrected *p*-value<0.05	
	Log-ratio < -1	Log-ratio > 1	Log-ratio < -1	Log-ratio > 1
**Group 4 vs Group 1**	159	168	182	204
**Group 4 vs Group 2**	185	211	212	268
**Group 4 vs Group 3**	264	324	291	400
**Group 3 vs Group 1**	98	139	119	152
**Group 3 vs Group 2**	8	50	8	50
**Group 2 vs Group 1**	22	19	19	12

Group 1: Control hMSC, Group 2: Day-4 GDF5-induced hMSC, Group 3: Day-10 GDF5-induced hMSC, Group 4: tenocytes.

The significantly up- and down- regulated genes were presented in the Venn diagrams to show the overlap between all the comparisons with: (1) control hMSC (Group 1; Fig 2D) and (2) tenocytes (Group 4; Fig 2D). The Venn diagrams showed 8 genes (as compared to control hMSC; Fig 2D) and 219 genes (as compared to tenocytes; Fig 2E) associated with tenogenic differentiation by GDF5 induction; of these 2 were up-regulated and 6 were down-regulated when compared to control hMSC (Fig 2D); and 123 were up-regulated and 96 were down-regulated when compared between tenocytes to hMSCs (Fig 2E). In addition, numerous genes associated with tenogenic differentiation which were modulated by GDF5 were identified in GDF5-induced hMSCs; both day 4 (17 up-regulated, 22 down-regulated) and day 10 (16 up-regulated, 9 down-regulated). The genes displayed the most significant changes in expression patterns in the GDF5-induced hMSCs and in tenocytes were listed in S6 Table with their respective log_2_ ratio (log_2_ fold change).

Among the significantly co-expressed genes by GDF5-induced hMSCs and tenocytes, the genes associated with actin cytoskeleton reorganization (stress fibers formation) or associated indirectly with collagen fibrillogenesis signalling i.e. chemokine (C-C motif) ligand 2 (CCL2) and rho GTPase activating protein 29 (ARHGAP29) were found to be among the top most up-regulated genes in day 4 GDF5-induced hMSCs (S6 Table). Besides, neurofilament medium polypeptide (NEFM) was among the most significantly down-regulated genes in day-4 and day-10 GDF5-induced hMSCs; although not appeared to be the top most down-regulated genes in tenocytes, it was significantly down-regulated in tenocytes. Further, the integrin alpha 8 (ITGA8), one of the subunit of the integrin α8β1 protein that mediate numerous cellular processes, including cytoskeleton rearrangement, was among the top most down-regulated gene in both GDF5-induced hMSCs and tenocytes. A complete list of genes significantly modulated during tenogenic differentiation by GDF5 induction can be found in S7 Table.

### Pathways associated with GDF5-induced tenogenic differentiation in hMSC

In this section, the signalling mechanisms involved in GDF5-induced hMSCs (day 4 and day 10) and in native tenocytes, as captured by pathway analysis are presented (S8 Table). The pathway analysis was based on the significantly up- or down- regulated gene lists where corrected-*p*<0.05 and at least fold change of 2 (or log_2_ ratio at least 1.0 for up-regulated genes) or fold change less than 0.5 (or log_2_ ratio less than -1.0 for down-regulated genes). A total of 8 pathways (*p*<0.001) were identified as associated with day 4 GDF5-induced hMSCs (cholesterol biosynthesis, glycolysis and gluconeogenesis p.3, TREM1 signalling pathway, IL-17 signalling pathways, VEGF signalling and insulin regulation of fatty acid metabolism).

At day 10 of GDF5 induction, a total of 21 pathways (*p*<0.001) were regulated, among which cell cycle related signalling pathways (i.e. the metaphase checkpoint signalling, chromosome condensation in prometaphase signalling, initiation of mitosis signalling as well as spindle assembly and chromosome separation signalling) were down-regulated and development related TGF-β-dependent induction of EMT via SMADs signalling, angiopoietin—Tie2 signalling, cytoskeleton remodelling keratin filaments signalling, arachidonic acid production signalling were activated.

The GDF5-induced hMSC (day 4 and 10) and tenocytes together showed regulation of 11 pathways (S8 Table). As an extension to determine the pathways associated with the late tenogenic differentiation or mature tenocytes, the significantly up- or down regulated gene lists obtained from comparing tenocytes to GDF5-induced hMSC were analyzed. In matured tenocytes, the activated pathways were: (i) development related TGF-β-dependent induction of EMT via SMADs signalling, TGF-β-dependent induction of EMT via RhoA, PI3K and ILK signalling, PEDF signalling, cross-talk between VEGF and angiopoietin 1 signalling, (ii) cell adhesion related ECM remodelling signalling, cell-matrix glycoconjugates signalling, Ephrin signalling, tight junctions signalling, cadherin-mediated cell adhesion signalling, PLAU signalling and (iii) cell cycle related (i.e. chromosome condensation in prometaphase signalling, role of APC in cell cycle regulation signalling, initiation of mitosis signalling, ATM/ATR regulation of G1/S checkpoint signalling, sister chromatid cohesion signalling and role of SCF complex in cell cycle regulation signalling) pathways. Whereas, the down-regulated pathways were muscle contraction delta-type opioid receptor in smooth muscle signalling, muscle contraction related GPCRs in the regulation of smooth muscle tone signalling, and development related S1P2 and S1P3 receptors in cell proliferation and differentiation signalling.

### Candidate Tenogenic and Non-Tenogenic Markers Expression Profiles

Apart from the most significantly up- or down- regulated genes and pathways, the changes in the expression profiles in ECM related genes as well as candidate tenogenic and non-tenogenic marker genes in GDF5-induced hMSCs were also analysed. Graphical representation of log_2_ ratios detected by microarray analysis is shown in Fig 3C. Among these genes, type-I collagen alpha 1 (*Col-I a1*), type-II collagen alpha 1 (*Col-II*), type-III collagen alpha 1 (*Col-III a1*) and matrix metallopeptidase 3 (*Mmp3*) were related to ECM; thrombospondin 4 (*Thbs4*), mohawk homeobox (*Mkx*), tenomodulin (*Tnmd*), scleraxis (*Scx*) and tenascin C (*Tnc*) were candidate tenogenic marker genes; peroxisome proliferator-activated receptor gamma (*Pparγ*), SRY (Sex determining region Y)-box 9 (*Sox9*), cartilage oligomeric matrix protein (*Comp*), Runt-related transcription factor 2 (*Runx2*), bone gamma-carboxyglutamate protein (*Bglap*) and alkaline phosphatase liver (*Alpl*) were the non-tenogenic marker genes; and c-fos induced growth factor (*Figf* or *Vegf-d*) were related to chondrogenesis [18], osteogenesis [19] and tenocyte proliferation [20].

The *Col-I* and *Col-III* were significantly up-regulated in the day-10 GDF5-induced hMSCs, among the ECM related genes. A down-regulation of *Col-I* in tenocytes indicated that the production of *Col-I* were reduced in mature tenocytes compared to the control hMSC and the early differentiating tenogenic hMSCs. Nevertheless, the *Mmp3*, which play a role in the normal maintenance and remodelling of tendon ECM, was up-regulated in the tenocytes, but weakly expressed in the GDF5-induced hMSCs. Among the tenogenic marker genes, only *Tnc* was detected as up-regulated in the GDF5-induced hMSCs. Whereas in the mature tenocytes, the *Thbs4* and *Mkx* was detected as highly up-regulated transcript compared to the other genes. The expression level of *Tnc* in tenocytes was higher than the control hMSCs despite not being above the cut-off level.

The non-tenogenic marker genes were generally weakly expressed among the GDF5-induced hMSC and tenocytes, except for *Comp* and *Runx2*. The expression levels of *Comp* was significantly up-regulated in day-10 GDF5-induced hMSC, while the *Runx2* was significantly down regulated in tenocytes. The rest of the other genes, including the *Figf*, were detected as weakly expressed and not significantly up- or down- regulated.

### Cytoskeletal reorganization in GDF5-induced hMSCs

**Fig 4:**
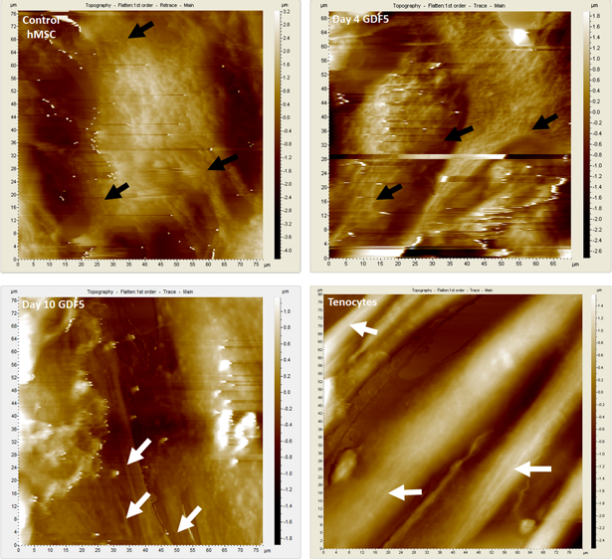
Cytoskeleton reorganization in hMSCs visualized by AFM. Representative AFM topography scanning of control hMSCs (left upper), hMSCs at day 4 of GDF5 induction (right upper), hMSCs at day 10 of GDF5 induction (left lower) and tenocyte. In the topography images, brighter colour indicated higher distance off of substrate (cover slip). The panel on the right side of each image indicated the height scale (z-scale) of the topography. There was a marked difference in the topography (cytoskeleton organization) of the control hMSCs compared to the hMSCs exposed to GDF5. The topography of control hMSCs had a larger z-scale; apparently possessed higher morphology. Both control hMSCs and day-4 GDF5-induced hMSCs showed detailed treelike web structure of presumably the actin network under the cell membrane especially at the leading edges which strongly attached on the cover slip (black arrowheads). The GDF5-induced hMSCs possessed more flatten morphology because they adhered more strongly via the stress fibers that could be visualized just under the surface of the cell membrane (white arrowheads). The detailed structure of presumably the actin cytoskeleton (actin bundles or stress fibers) could be observed in the day-10 GDF5-induced hMSCs and tenocytes.

To further evaluate the changes in the cytoskeleton reorganization in tenogenic differentiation in GDF5-induced hMSCs, as suggested by the co-expressed genes in GDF5-induced hMSCs and tenocytes microarray results, AFM live cell imaging was conducted. The control hMSCs, day4 GDF5-induced hMSCs, day-10 GDF5-induced hMSCs and tenocytes were cultured in their respective media when scanned with AFM. Upon differentiation, there were evident differences in the topography between the undifferentiated hMSCs and their GDF5-induced counterparts (Fig 4). Overall, the topography scan revealed a higher height scale in control hMSCs as compared to that of the GDF5 treated hMSCs and tenocytes, which may particularly related to their cytoskeleton organization.

In day-4 GDF5-induced hMSCs, the cell surface topography was similar to that of control hMSCs; with a treelike web structure of presumably actin network. There was a tremendous change in hMSCs cell surface topography at day 10 of GDF5 induction. The day-10 GDF5-induced hMSCs showed similar cell surface topography to that of tenocytes, which both revealed bundle structure of presumably the stress fibers at the leading edge. These findings suggested that the hMSCs underwent tenogenic differentiation with continued cytoskeleton reorganization which allowed them to adhere more strongly to the substrate (cover slip) and subsequently displayed a more flatten morphology compared to the control hMSCs. To further confirm that the structures visualized under AFM were actin filaments, fluorescence imaging was conducted.

**Fig 5:**
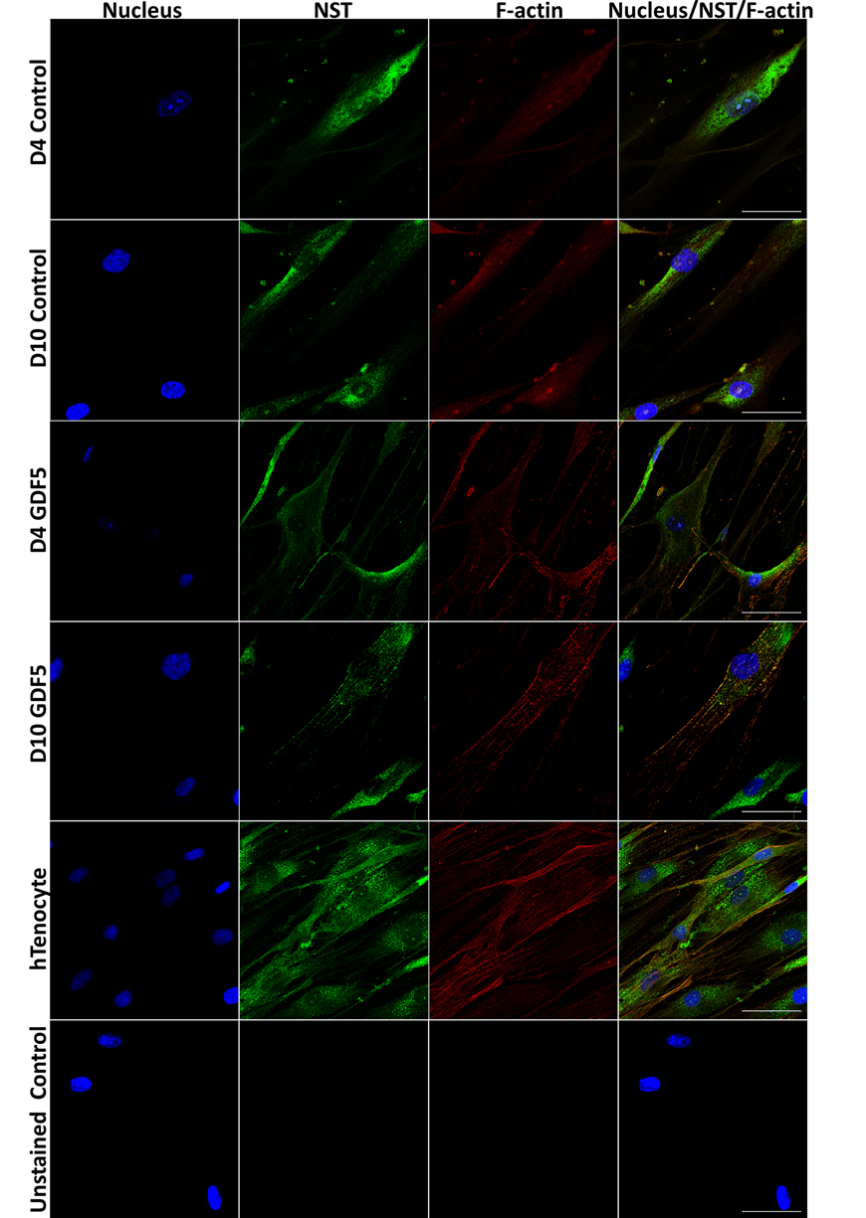
Actin cytoskeleton reorganization and nucleostemin (NST) expression in hMSCs upon GDF5 induction captured with confocal laser scanning microscope. Representative images of sequential scanning: nucleus stained with Hoescht 33342 (first panel on the left), nucleostemin (NST) (with indirect FITC stain; second panel) and actin fibres (direct staining which specifically stained cellular F-actin; third panel) and the merged image of all channels (last panel on the right). Scale bar = 50μm (at 100x objective).

The fluorescence images obtained by CLSM (Fig 5) shadowed those images obtained by AFM imaging (Fig 4). The CLSM analysis demonstrated that the GDF5-induced hMSCs possessed stress fiber arrays which localised primarily next to the cell attachment site (Fig 5). However the abundance of this stress fiber arrays appeared low in the control hMSCs, which displayed more cortical cytoplasmic actin (or actin filament meshwork). Following extended GDF5 induction on day 10, hMSCs displayed long, thin stress fibres, similar to that in tenocytes. These observations suggested that GDF5-induced a reorganization of actin structures which involved F-actin polymerization and restructuring of cortical actin elements to newly formed stress fibres.

The nucleostemin (NST) was used as a marker for proliferating stromal stem cells [21]. The NST expression was decreased in day-4 and day-10 GDF5-induced hMSCs (Fig 5). The control hMSCs persistently expressed NST at day 4 and day 10 but the GDF5-induced hMSCs showed a reduction in the NST expression, which is parallel with the cytoskeletal reorganization in hMSCs. This finding suggested that the proliferation of hMSCs reduced upon tenogenic differentiation.

## Discussion

Herein, the molecular signalling pathways regulated during GDF5-induced tenogenic differentiation were identified. Firstly, a common set of genes up-regulated in GDF5-induced hMSCs and tenocytes were identified, i.e. ARHGAP29 and CCL2; and the genes consistently down-regulated in all groups were ITGA8, NEFM, C7orf69, ACAN, STC1 and ENO2. Secondly, through the global gene expression profiles analysis, several pathways were identified as important pathways for tenogenic differentiation: (i) the glycolysis and gluconeogenesis signalling pathways were down regulated upon GDF5 induction in hMSC and in tenocytes; (ii) the cell cycle related signalling pathways were also down-regulated in the day-10 GDF5-induced hMSCs; (iii) the activated pathways which may be crucial in tenogenic differentiation were agiopoietin-Tie2 signalling, TGF-beta-dependent induction if EMT via SMADS signalling, PEDF signalling and VEGF signalling via VEGFR2; (iv)the cell adhesion and cytoskeleton remodelling signalling as well as EMT pathways were identified as important pathways at the late tenogenic differentiation stage or in mature tenocytes. Thirdly, among the candidate tenogenic marker genes, *Col-I*, *Col-III* and *Tnc* were up-regulated in the day-10 GDF5-induced hMSCs; while the *Runx2*, the non-tenogenic marker gene, was down-regulated. Fourthly, the AFM and fluorescence microscopy imaging evidenced the cytoskeletal remodelling events in the GDF5-induced hMSCs.

A previous study reported that *Thbs4*, *Tnmd*, *Dcn* and *Mkx* were among the top molecular markers of mature human tendon [22]. In consistent to that report [22], by Jelinsky and colleagues (2010), the results of this current experiment also found *Thbs4* and *Mkx* as the top most up-regulated transcripts in tenocytes. However, the *Thbs4* and *Mkx* were not up-regulated in the GDF5-induced hMSCs. It is reasoned that these markers are the late tenogenic markers, and therefore not expressed in the tenogenic hMSCs.

Amongst the pathways associated with day 4 GDF5-induced hMSC, of particular relevance is the activation of vascular endothelial growth factor (VEGF) signalling via VEGFR2 generic cascade. VEGF is expressed in tendon sheath fibroblasts and the expression of this growth factor increased in early tendon healing process [23]. It is one of the important regulators of gene activation in *Col-I* synthesis [24]. Activation of this pathway may thus potentially associate with early stage of tenogenic differentiation induced by GDF5. The down-regulation in the glycolysis and gluconeogenesis pathways found in GDF5-induced hMSCs could be explained by MSCs are more glycolytic than differentiated fibroblasts [25]. In addition, interestingly, lipid metabolism related pathway were also identified. This was consistent with previous study on osteogenic differentiation of porcine adipose tissue derived stem cells [26]. We therefore suggest that lipid metabolism may be an important event during early stem cell differentiation.

Some cell cycle related signalling pathways were down-regulated in day 10 GDF5-induced hMSCs (S8 Table). Extensive cell-cell contact or depletion of nutrients from the culture medium has been shown to induce transient/reversible growth arrest (or cell cycle arrest), however, a more physiological mechanism to regulate cell proliferation occurs in stem cells in association with their differentiation. The growth arrest in the G_1_ phase of the cell cycle has been reported to be associated with expression of the differentiated phenotype in many cell types [27–29]; and the stem cells must growth arrest (predifferentiation growth arrest) at a distinct cell cycle state prior to differentiation [28]. Thus, the down-regulation of cell cycle related pathways at day 10 of GDF5 induction was not unexpected. The activation of angiopoietin—Tie2 signalling together with the down regulation of cell cycle related pathway, in the day 10 GDF5-induced hMSCs, may suggest that the angiopoietin—Tie2 signalling play a protective role when the hMSC exit the cell cycle and undergo differentiation. Angiopoietin—Tie2 signalling pathway has been demonstrated to play a critical role in the maintenance of hematopoietic stem cells in a quiescent state in the bone marrow niche [30] and it also has a protective effect on MSC which is crucial in MSC survival [31].

The developmental related pathway identified in day 10 GDF5-induced hMSCs, EMT pathway, although plays crucial roles in the formation of body plan (a characteristic process of vertebrate gastrulation) [32] and in the differentiation process of multiple tissue and organs[33], its role for adult stem cells (ie. hMSC) to differentiate into tenogenic lineage remains unknown. The occurrence of EMT have been reported in initiation of human liver development [34] as well as in epicardiac cells in the adult human heart [35]. However, to date, no studies have reported on the role of EMT in tenogenesis or in the differentiation of mesenchymal stem cells into mesenchymal lineage.

An interesting observation in the day 10 GDF5-induced hMSCs is the activation of cytoskeleton remodelling keratin filaments signalling. The activation of this pathway may suggest a crucial role of keratin filament reorganization in hMSCs during early tenogenic differentiation. Rapid keratin-network adaptation has recently been reported to be crucial in migrating cells and for adaptation to varying environment conditions for example, during development or under mechanical stress in epithelia [36] and hepatocyte [37, 38]. However, the function of keratin filaments signalling in tenogenic differentiation is unclear.

The activation of arachidonic acid signalling in the GDF5-induced hMSCs may play an essential role in tenogenic differentiation. This alludes to the arachidonic acid is an initial molecule in a cascade that involved phospholipase A_2_ (PLA_2_) and produces prostaglandin-E_2_ (PGE_2_) [39]. This PGE_2_ has an effect in the proliferation and collagen production of human tendon fibroblast [40]. We therefore suggested that the arachidonic acid production signalling play an important role in tenocyte behaviour. Moreover, cytosolic PLA_2_ (cPLA_2_) and secretory PLA_2_ (sPLA_2_) are involved in the production of other inflammatory mediators, apart from the PGE_2_. Therefore, this could possibly explain the occurrence of the immune response pathways identified in this current experiment.

Based on the common pathways identified in GDF5-induced hMSCs (day 4 and 10) and tenocytes, the down-regulation of glycolysis signalling in the GDF5-induced hMSCs is thereby relevant to tenogenic differentiation; as the same down-regulation observed in the day-4 GDF5-induced hMSC was observed in tenocytes. This again explained that the hMSCs are more glycolytic than primary human tenocytes. Thus, upon GDF5 induction, the hMSCs underwent tenogenic differentiation, which subsequently exhibited lower glycolytic activities than the control hMSCs. In addition, of particular relevance was the activation of development related angiopoietin—Tie2 signalling, TGF-beta-dependent induction of EMT via SMADs, VEGF signalling via VEGFR2 and pigment epithelium-derived factor (PEDF) signalling. It is suggested that activation of these pathways would promotes tenogenic differentiation. Among these pathways, the PEDF is a new identified adipokine. At physiological concentration, this protein inhibits adipocyte differentiation, and down regulates the adipocyte markers [41]. Therefore, the activation of this PEDF signalling pathway was suggested to be crucial to promote tenogenic differentiation in GDF5-induced hMSCs. Furthermore, the cell adhesion signalling (e.g. tight junctions signalling) and cytoskeletal remodelling signalling (e.g. regulation of actin cytoskeleton by Rho GTPases signalling) were also activated in the GDF5-induced hMSCs and in native tenocytes (not shown in table); despite at a lower significance level (*p* = 0.01 and *p* = 0.05, respectively). These pathways were associated with tenocyte behaviour as cell adhesion and cytoskeletal remodelling are particularly important in the survival of the tendon cells which reside in the high tensional loading tissue.

By comparing the tenocytes to the GDF5-induced hMSC, the activated pathways which were development related were consistent to the pathways identified from the day-4 or day-10 GDF5-induced hMSCs, except the PEDF signalling. PEDF signalling was only up-regulated at in the tenocytes. This suggested that the PEDF signalling may be involved in the late tenogenic differentiation or in mature tenocytes phenotypes. However, in contrary to the effect of day 10 GDF5 induction (or early tenogenesis), the cell cycle related pathways were up-regulated in the mature tenocytes. Activation of cell cycle signalling in tenocytes suggested that active maintenance of cell-cycle as an important aspect of the differentiated tenocytes and there may be a temporal coupling between withdrawal from the cell cycle and tenogenic differentiation as previously described in stem cells differentiation events [28, 29]. It is therefore postulated that there is a mechanism for coordinating cell cycle and differentiation events during tenogenic differentiation, which remains to be discovered. Apart from the development and cell cycle related signalling, the activated pathways also involved ECM remodelling signalling and cell adhesion signalling which were two crucial phenotypes that the mature tenocytes must possess. These characteristics are crucial for the tenocytes to maintain its integrity and physical resistance to mechanical stress in their native tendon tissue. Hence, the conversion of biochemical signals (GDF5 induction) into the cytoskeletal remodelling is important for maturation of tenogenic hMSCs, especially in cytoskeletal-ECM linkage. The pathways related to ECM remodelling via direct or indirect connections between internal actin cytoskeleton and ECM in tenocytes were such as cell adhesion related integrin inside-out signalling, cytoskeleton remodelling signalling and regulation of actin cytoskeleton by Rho GTPases signalling, which involved significantly regulated transcripts, i.e. type-I collagen, alpha-2/beta-1 integrin, alpha-10/beta-1 integrin, actin and laminin 1. The regulation of actin cytoskeleton by Rho GTPases signalling has been implicated in lamellipodium and stress fiber formation in mammalian cells [42, 43]. Activation of this pathway may thus potentially be involved in the lamellipodium and stress fiber formation in the mature tenocytes. Other cell adhesion related pathways activated in the mature tenocytes (cell-matrix glycoconjugates, ephrin signalling, tight junctions, cadherin-mediated cell adhesion and PLAU signalling) also play an important role in cytoskeleton-ECM linkage in tenocytes. Down regulation of muscle contraction and development related signalling were consistent with mature tenocyte phenotype.

We therefore inferred based on the gene expression profile analysis that cytoskeletal remodelling signalling and cell adhesion signalling are essential signalling pathways for hMSCs tenogenic differentiation, particularly in the expression of the earliest tenogenic markers in hMSC. Development of the cellular cytoskeleton during the tenogenic differentiation has been shown by previous study in uniaxial-cyclic-stretched hMSCs, with observations of actin stress fibers in the stretched hMSCs [44]. This effect however, was observed in this current experiment in the GDF5-induced hMSCs. Therefore, it is suggested that the cytoskeleton remodelling is an essential event in tenogenic differentiation and for the tenocyte phenotypic expression.

In the event of tenogenic differentiation, the proliferation of hMSCs was reduced as suggested by the reduced in NST expression in hMSCs underwent tenogenic differentiation. This finding is relevant to the pathway analysis which demonstrated a down-regulation in the cell cycle related signalling pathways in the GDF5-induced hMSCs. The available evidence has been reported that growth arrest in G_1_ phase of the cell cycle is associated with expression of the differentiated phenotype in many cell types [27, 45]. Hence, it is suggested that a temporal coupling of cell cycle arrest and terminal differentiation occurs during the tenogenic differentiation in hMSCs. This study does not seek to provide an exhaustive elucidation of how the cell-cycle and stem cell differentiation events are coordinated in tenogenic differentiation, instead keeping more to analysing the gene expression profiles of hMSCs tenogenic differentiation. Therefore, no further analysis on cell cycle or cell proliferation analysis was conducted to evidence this speculation. Nevertheless, a more comprehensive study is required in order to demonstrate how the coordination of cell-cycle arrest and differentiation is achieved. This would subsequently contribute to the identification of known developmental regulators or pathways that direct link these two events, particularly in hMSCs tenogenic differentiation.

A possible limitation in this current experiment is that the assessment of cytoskeleton rearrangement by CLSM were not conducted on the same area or same sample scanned by AFM. Ideally, a better experimental approach to evidence the AFM topography results is to assess the same area scanned by AFM for CLSM imaging. However, due to the limitation in the equipment used in the current experiment, the assessment of cytoskeleton rearrangement on the same cell or same scanned area by the AFM was not possible. Nevertheless, the samples independently prepared for AFM and CLSM in the current experiment allowed an independent validation of AFM results by CLSM. Furthermore, the independent sample preparation for AFM and CLSM imaging allowed the advantages of minimally prepared cultured cells (i.e. without any staining) to be used for AFM live cell imaging, hence reflected closer to the physiological condition. A recent study reported on evaluation of tenogenic differentiation by AFM analysis were also conducted on samples independently prepared for AFM and CLSM [44]. In addition, due to the instrumentation constrain at the time that this experiment was conducted, the cells were gently treated with glutaraldehyde (0.5%) for 2 h at 37°C prior to AFM imaging. A previous study has reported that even 0.5% glutaradehyde treatment for 60s on cells is able to dramatically increase the elastic modulus measured by AFM, however, accompanied by an apparent improvement in imaging reproducibility while still allowing structural information to be obtained [46]. In the light of the glutaraldehyde treatment in this study was to improve the imaging quality and the quantitative elastic modulus of cells were not measured in this study, thereby the glutaraldehyde treatment is appropriate in this study. Nevertheless, further study is required in order to systematically assess the effect of fixation levels on AFM imaging or elastic modulus measurement in tenogenic MSC.

## Conclusions

In conclusion, this study shed light on the possible signalling pathways involved in GDF5-induced hMSC tenogenic differentiation and evidenced that the cytoskeleton remodelling occurring in the early tenogenic differentiation. The top most up- or down- regulated genes identified in early tenogenenic hMSCs or in late mature tenocytes are potentially to be used as molecular markers in future studies related to tenogenic differentiation. Nevertheless, a lot more remain to be explored about the tenogenic differentiation events in hMSCs, for instance, the cell adhesion force change during the MSC-to-tenocyte differentiation.

## Supporting Information

S1 Fig Microarray workflow from sample preparation to data analysis and validation. Total RNA were extracted from all the samples and pre-determined for their concentration and integrity before proceed to cDNA amplification and labelling. All the labelled cDNA samples were used for targets preparation. The prepared targets were subsequently hybridized to the arrays, followed by washed, stained and scanned to get the image files. The captured microarray image files were analysed via GCOS (Command Console and Expression Console; Affymetrix Inc, Santa Clara, CA, USA) to get the CEL intensity files. The CEL intensity files were then summarized via data pre-processing to get the Robust Multiarray Average (RMA) signals (expression values). The significantly differentially expressed genes were detected via Limma analysis (Smyth, 2004). Pathway analysis was conducted with Partek® Genomic Suite™ 6.6 beta and GeneGO Metacore™ Pathway Analysis software. The microarray data was validated with AFM and fluorescence imaging and QuantiGene gene expression analysis.
(PDF)

S2 Fig Closed cell incubation sample plate for atomic force microscopy imaging. The closed cell incubation sample plate was used to incubate the cells during the entire imaging process.
(PDF)

S3 Fig Pre-processing and quality control for microarray data. (A) Positive versus negative ratio of all arrays showed the efficiency and specificity of the hybridization in all arrays. Ideally, the value of positive versus negative control should be 1. The results showed that the efficiency and the specificity of the hybridization in all arrays were in the acceptable range (≥0.8). (B) Spike-in hybridization control plots showed similar intensity in all arrays. All arrays were able to detect the spike-in hybridization controls in accordance to their respective spike-in quantities (CreX, BioD, Bio C and Bio B), indicated that all arrays possessed comparable sensitivity in detecting the high and low abundant genes. (C) Histogram of perfect match for all arrays showed the overall higher or lower intensities in all the 24 arrays, with no saturation effects. These were the intensities of the probes, prior to normalization and not combined to the probe sets yet. The results showed a typical distribution of signal intensities; they were never normally distributed. As this is a whole genome array, a lot of cell-specific genes were not expressed, leading to a lot of probes that gave very low (or no) signal, so the distribution curves of the perfect match intensities were positively skewed. (D) Boxplots of log_2_ ratios for perfect match intensities of all arrays. Although some samples, e.g. “hyb02” and “hyb29” showed slightly thinker/longer tail than the other samples, all the arrays showed comparable distributions, and no sample was identified as outlier. (E) The bar chart of the percentages of detectable above background (DABG) scores for present calls in all the arrays. The percentages of DABG ranged within less than 10% difference showed that the hybridization in all arrays was of superior quality and DABG among all the arrays were comparable.
(PDF)

S4 Fig Heatmap and dendrogram of RMA expression values. (A) The heatmap of RMA values showed comparable level of expression of all the genes across all the 24 arrays. The tree diagram on the upper panel of the heatmap showed the distances between the samples. The colour of the heatmap indicated the between-array distances. A colour bar with scales for the heatmap is included, indicating that red corresponds to maximum distance and green to minimum distance. (B) The dendrogram plot indicates the Euclidean distance and complete linkage with all individual samples. (C) The dendrogram plot indicates the Euclidean distance and complete linkage with average of the four groups.
(PDF)

S1 Table Basic demographics and the origin of tissue samples for hMSCs cultures from the donors. (PDF)

S2 Table Reagents used for immunofluorescence staining for fluorescence imaging. (PDF)

S3 Table QuantiGene® Plex 2.0 Assay (311904–215 Human) Reagent System. (PDF)

S4 Table Summary of total number of probe sets or genes before and after data normalization and filtering. (PDF)

S5 Table A summary of the number of differentially expressed probe sets. (PDF)

S6 Table The most significantly altered genes in the GDF5-induced hMSC and tenocytes [LR: log2 ratio, p-value (corr): corrected p-value]. (PDF)

S7 Table List of Genes Modulated in hMSCs by GDF5 Treatment and Genes Modulated in Tenocytes (Total: 873 genes). (PDF)

S8 Table Pathways regulated by GDF5-induced tenogensis in hMSC. (PDF)

## Abbreviations

AFM: Atomic force microscopy
ARHGAP29: rho GTPase activating protein 29
BMP: bone morphogenetic protein
CCL2: chemokine (C-C motif) ligand
CLSM: Confocal laser scanning microscopy
COL-I: type-I collagen
COL-III: type-III collagen
CR: Cytoskeletal reorganization
DCN: decorin
DMEM: Dulbecco’s modified Eagle’s medium
FBS: fetal bovine serum
GAPDH: glyceraldehyde-3-phosphate dehydrogenase
GDF5: growth and differentiation factor 5
hMSC: human bone marrow stromal cell
NST: nucleostemin
RUNX2: runt-related transcription factor 2
SCX: scleraxis
S.D.: standard deviation
SOX9: SRY (sex determining region Y)-box 9
TNC: tenascin-C
