# Supplementary material for: Identification of Pathways Mediating Growth Differentiation Factor5-Induced Tenogenic Differentiation in Human Bone Marrow Stromal Cells
Source: PLoS One. 2015 Nov 3;10(11):e0140869. doi: 10.1371/journal.pone.0140869 (PMC4631504; doi:10.1371/journal.pone.0140869)
Supplement: S1 Fig — Total RNA were extracted from all the samples and pre-determined for their concentration and integrity before proceed to cDNA amplification and labelling. All the labelled cDNA samples were used for targets preparation. The prepared targets were subsequently hybridized to the arrays, followed by washed, stained and scanned to get the image files. The captured microarray image files were analysed via GCOS (Command Console and Expression Console; Affymetrix Inc, Santa Clara, CA, USA) to get the CEL intensity files. The CEL intensity files were then summarized via data pre-processing to get the Robust Multiarray Average (RMA) signals (expression values). The significantly differentially expressed genes were detected via Limma analysis (Smyth, 2004). Pathway analysis was conducted with Partek® Genomic Suite™ 6.6 beta and GeneGO Metacore™ Pathway Analysis software. The microarray data was validated with AFM and fluorescence imaging and QuantiGene gene expression analysis. (PDF) [file pone.0140869.s001.pdf]

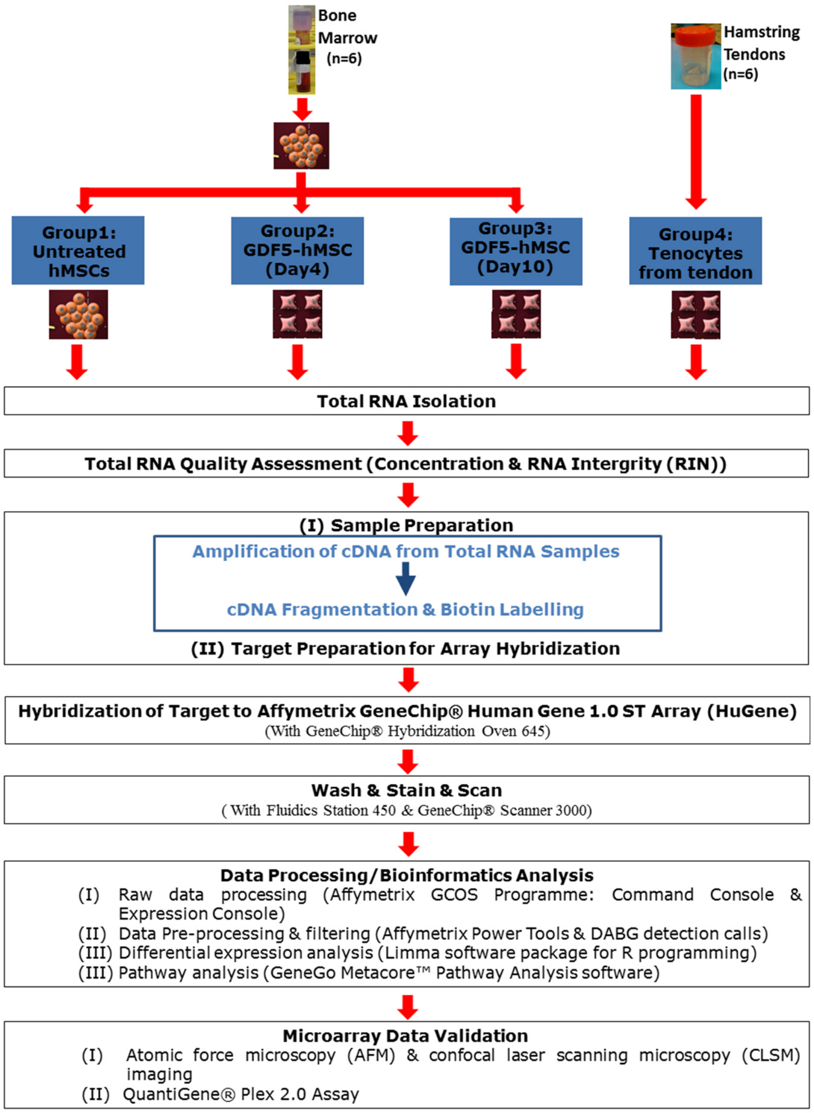

S1 Fig. Microarray workflow from sample preparation to data analysis and validation. Total RNA were extracted from all the samples and pre-determined for their concentration and integrity before proceed to cDNA amplification and labelling. All the labelled cDNA samples were used for targets preparation. The prepared targets were subsequently hybridized to the arrays, followed by washed, stained and scanned to get the image files. The captured microarray image files were analysed via GCOS (Command Console and Expression Console; Affymetrix Inc, Santa Clara, CA, USA) to get the CEL intensity files. The CEL intensity files were then summarized via data pre-processing to get the Robust Multi-array Average (RMA) signals (expression values). The significantly differentially expressed genes were detected via Limma analysis (Smyth, 2004). Pathway analysis was conducted with Partek ® Genomic Suite™ 6.6 beta and GeneGO Metacore™ Pathway Analysis software. The microarray data was validated with AFM and fluorescence imaging and QuantiGene gene expression analysis.
