# Supplementary material for: Identification of Pathways Mediating Growth Differentiation Factor5-Induced Tenogenic Differentiation in Human Bone Marrow Stromal Cells
Source: PLoS One. 2015 Nov 3;10(11):e0140869. doi: 10.1371/journal.pone.0140869 (PMC4631504; doi:10.1371/journal.pone.0140869)
Supplement: S2 Fig — The closed cell incubation sample plate was used to incubate the cells during the entire imaging process. (PDF) [file pone.0140869.s002.pdf]

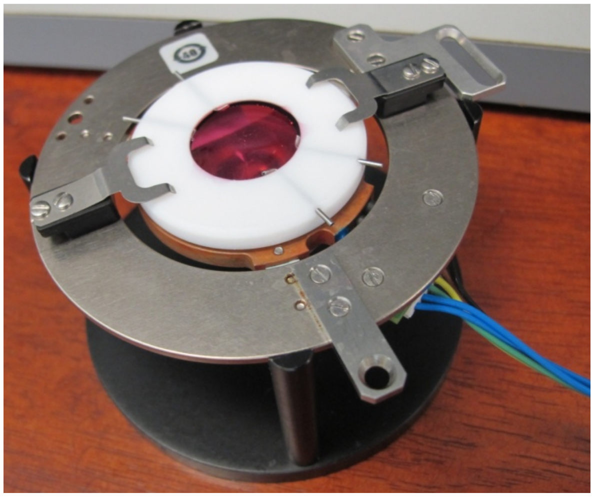

S2 Fig. Closed cell incubation sample plate for atomic force microscopy imaging. The closed cell incubation sample plate was used to incubate the cells during the entire imaging process.
