# Supplementary material for: Identification of Pathways Mediating Growth Differentiation Factor5-Induced Tenogenic Differentiation in Human Bone Marrow Stromal Cells
Source: PLoS One. 2015 Nov 3;10(11):e0140869. doi: 10.1371/journal.pone.0140869 (PMC4631504; doi:10.1371/journal.pone.0140869)
Supplement: S3 Fig — (A) Positive versus negative ratio of all arrays showed the efficiency and specificity of the hybridization in all arrays. Ideally, the value of positive versus negative control should be 1. The results showed that the efficiency and the specificity of the hybridization in all arrays were in the acceptable range (≥0.8). (B) Spike-in hybridization control plots showed similar intensity in all arrays. All arrays were able to detect the spike-in hybridization controls in accordance to their respective spike-in quantities (CreX, BioD, Bio C and Bio B), indicated that all arrays possessed comparable sensitivity in detecting the high and low abundant genes. (C) Histogram of perfect match for all arrays showed the overall higher or lower intensities in all the 24 arrays, with no saturation effects. These were the intensities of the probes, prior to normalization and not combined to the probe sets yet. The results showed a typical distribution of signal intensities; they were never normally distributed. As this is a whole genome array, a lot of cell-specific genes were not expressed, leading to a lot of probes that gave very low (or no) signal, so the distribution curves of the perfect match intensities were positively skewed. (D) Boxplots of log2 ratios for perfect match intensities of all arrays. Although some samples, e.g. “hyb02” and “hyb29” showed slightly thinker/longer tail than the other samples, all the arrays showed comparable distributions, and no sample was identified as outlier. (E) The bar chart of the percentages of detectable above background (DABG) scores for present calls in all the arrays. The percentages of DABG ranged within less than 10% difference showed that the hybridization in all arrays was of superior quality and DABG among all the arrays were comparable. (PDF) [file pone.0140869.s003.pdf]

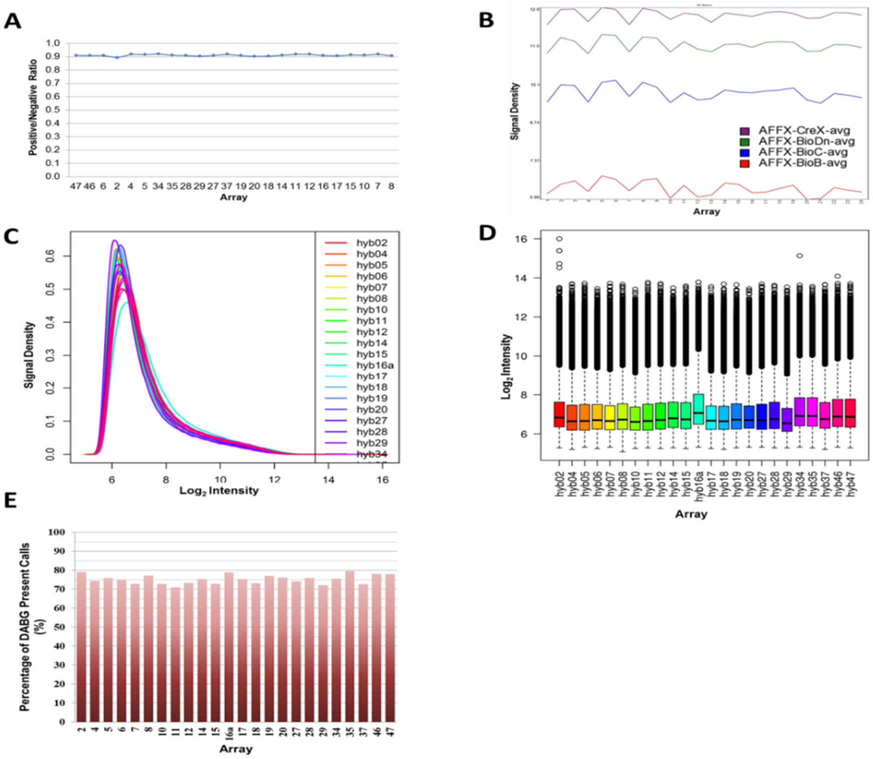


**S3 Fig. Pre-processing and quality control for microarray data.** (A) Positive versus negative ratio of all arrays showed the efficiency and specificity of the hybridization in all arrays. Ideally, the value of positive versus negative control should be 1. The results showed that the efficiency and the specificity of the hybridization in all arrays were in the acceptable range (≥0.8). (B) Spike-in hybridization control plots showed similar intensity in all arrays. All arrays were able to detect the spike-in hybridization controls in accordance to their respective spike-in quantities (CreX, BioD, Bio C and Bio B), indicated that all arrays possessed comparable sensitivity in detecting the high and low abundant genes. (C) Histogram of perfect match for all arrays showed the overall higher or lower intensities in all the 24 arrays, with no saturation effects. These were the intensities of the probes, prior to normalization and not combined to the probe sets yet. The results showed a typical distribution of signal intensities; they were never normally distributed. As this is a whole genome array, a lot of cell-specific genes were not expressed, leading to a lot of probes that gave very low (or no) signal, so the distribution curves of the perfect match intensities were positively skewed. (D) Boxplots of $\log_2$ ratios for perfect match intensities of all arrays. Although some samples, e.g. "hyb02" and "hyb29" showed slightly thinker/longer tail than the other samples, all the arrays showed comparable distributions, and no sample was identified as outlier. (E) The bar chart of the percentages of detectable above background (DABG) scores for present calls in all the arrays. The percentages of DABG ranged within less than 10% difference showed that the hybridization in all arrays was of superior quality and DABG among all the arrays were comparable.
