# Supplementary material for: Identification of Pathways Mediating Growth Differentiation Factor5-Induced Tenogenic Differentiation in Human Bone Marrow Stromal Cells
Source: PLoS One. 2015 Nov 3;10(11):e0140869. doi: 10.1371/journal.pone.0140869 (PMC4631504; doi:10.1371/journal.pone.0140869)
Supplement: S4 Fig — (A) The heatmap of RMA values showed comparable level of expression of all the genes across all the 24 arrays. The tree diagram on the upper panel of the heatmap showed the distances between the samples. The colour of the heatmap indicated the between-array distances. A colour bar with scales for the heatmap is included, indicating that red corresponds to maximum distance and green to minimum distance. (B) The dendrogram plot indicates the Euclidean distance and complete linkage with all individual samples. (C) The dendrogram plot indicates the Euclidean distance and complete linkage with average of the four groups. (PDF) [file pone.0140869.s004.pdf]

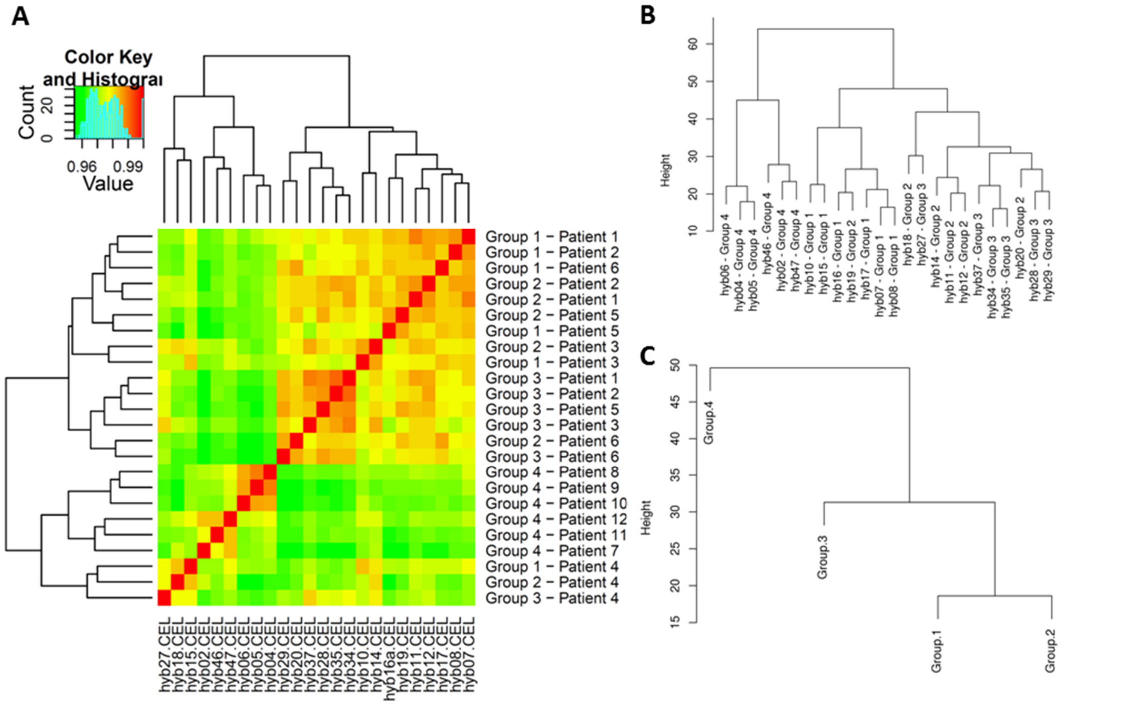

S4 Fig. Heatmap and dendrogram of RMA expression values. (A) The heatmap of RMA values showed comparable level of expression of all the genes across all the 24 arrays. The tree diagram on the upper panel of the heatmap showed the distances between the samples. The colour of the heatmap indicated the between-array distances. A colour bar with scales for the heatmap is included, indicating that red corresponds to maximum distance and green to minimum distance. (B) The dendrogram plot indicates the Euclidean distance and complete linkage with all individual samples. (C) The dendrogram plot indicates the Euclidean distance and complete linkage with average of the four groups.
