# Supplementary material for: Identification of Pathways Mediating Growth Differentiation Factor5-Induced Tenogenic Differentiation in Human Bone Marrow Stromal Cells
Source: PLoS One. 2015 Nov 3;10(11):e0140869. doi: 10.1371/journal.pone.0140869 (PMC4631504; doi:10.1371/journal.pone.0140869)
Supplement: S1 Table — (PDF) [file pone.0140869.s005.pdf]

S1 Table. Basic demographics and the origin of tissue samples for hMSCs cultures from the donors.

Donor	Age (year)	Gender	Sampling Site	Experiments
10 (h78)	63	Female	Bone marrow from femur	For *in vitro* tenogenic differentiation with GDF-5 induction in hMSC (microarray analysis) and imaging analysis.
11 (h79)	53	Female	Bone marrow from tibia	
12 (h74)	75	Female	Bone marrow from tibia	
13 (h76)	62	Female	Bone marrow from tibia	
14 (h83)	85	Female	Bone marrow from femur	
15 (h90)	65	Female	Bone marrow from tibia	
16 (02h23)	21	Male	Hamstring tendon	As positive control for *in vitro* tenogenic differentiation of hMSC (microarray analysis) and imaging analysis.
17 (02h16)	20	Male	Hamstring tendon	
18 (02h27)	29	Female	Hamstring tendon	
19 (02h33)	26	Male	Hamstring tendon	
20 (02h07)	23	Male	Hamstring tendon	
21 (02h17)	28	Male	Hamstring tendon	
