# Supplementary material for: Identification of Pathways Mediating Growth Differentiation Factor5-Induced Tenogenic Differentiation in Human Bone Marrow Stromal Cells
Source: PLoS One. 2015 Nov 3;10(11):e0140869. doi: 10.1371/journal.pone.0140869 (PMC4631504; doi:10.1371/journal.pone.0140869)
Supplement: S2 Table — (PDF) [file pone.0140869.s006.pdf]

S2 Table. Reagents used for immunofluorescence staining for fluorescence imaging.

No	Antibody	Dilution	Catalogue No.	Manufacturer
1	Type-I collagen mouse mAb	1:200	GTX26308	GeneTex, Inc, Irvine, CA
2	Type-II collagen mouse mAb	1:200	CP18	Calbiochem, Darmstadt, Germany
3	Type-III collagen mouse mAb	1:200	CP19	Calbiochem, Darmstadt, Germany
4	Nucleostamin (9D5.3) mouse mAb	1:200	ab78129	Abcam, UK
5	Tenascin C (EB2) mouse mAb	1:200	ab88280	Abcam, UK
6	Scleraxis (D-14) goat polyclonal antibody	1:200	sc-87425	Santa Cruz Biotechnology, California
7	Tenomodulin (C-20) goat polyclonal antibody	1:200	sc-49324	Santa Cruz Biotechnology, California
8	Fluorescein isothiocyanate (FITC)-conjugated rat anti-mouse IgG (A85-1)	1:200	553443	BD Biosciences, US
9	Texas red-conjugated donkey anti-goat IgG	1:200	sc-2783	Santa Cruz Biotechnology, California
10	Alexa Fluor® 546 phalloidin	1:40	A22283	Invitrogen, Carlsbad, California, USA
11	Hoechst 33342, trihydrochloride, trihydrate (10 mg/ml)	5 µg/ml	H3570	Invitrogen, Carlsbad, California, USA
12	FluoroGel mounting medium	-	GTX28214	GeneTex, Inc, Irvine, CA
