# Supplementary material for: Identification of Pathways Mediating Growth Differentiation Factor5-Induced Tenogenic Differentiation in Human Bone Marrow Stromal Cells
Source: PLoS One. 2015 Nov 3;10(11):e0140869. doi: 10.1371/journal.pone.0140869 (PMC4631504; doi:10.1371/journal.pone.0140869)
Supplement: S3 Table — (PDF) [file pone.0140869.s007.pdf]

S3 Table. QuantiGene® Plex 2.0 Assay (311904-215 Human) Reagent System

Bead Number	Gene Symbol	Genbank Accession No	Seq Length	Probe set region	Type	Target Name
13	RUNX2	NM_004348	5720	648-1082	Target	runt-related transcription factor 2
14	MMP3	NM_002422	1828	1119-1775	Target	Matrix metallopeptidase 3
20	SCXA	NM_001008271	606	251-574	Target	Homo sapiens scleraxis homolog A (mouse) (SCXA)
21	COMP	NM_000095	2471	1420-1842	Target	Homo sapiens cartilage oligomeric matrix protein (COMP), mRNA
25	PGK1	NM_000291	2439	1609-2233	HKG	phosphoglycerate kinase 1 (Medium High abundant HKG)
27	COL2A1	NM_001844	5087	1788-2258	Target	Type-II alpha 1 (primary osteoarthritis, spomdyloepiphyseal dysplasia, congenital)
30	FIGF	NM_004469	2128	835-1476	Target	c-fos induced growth factor (vascular endothelial growth factor D)
34	HPRT1	NM_000194	1435	102-646	HKG	Hypoxanthine phophoribosyltransferase 1 (Medium abundant HKG)
43	ALPL	NM_000478	2596	1075-1617	Target	alkaline phosphatase, liver/bone/kidney
44	BGLAP	NM_199173	552	113-477	Target	bone gamma-carboxyglutamate (gla) protein
45	PPARG	NM_005037	1818	567-1044	Target	peroxisome proliferative activated receptor, gamma
46	TNC	NM_002160	8605	2687-3165	Target	tenascin C

53	SOX9	NM_000346	3963	1607-2274	Target	SRY(Sex determining region Y)-box 9 (campomelic dysplasia, autosomal sex-reversal)
56	TNMD	NM_022144	1360	301-1020	Target	Homo sapiens tenomodulin (TNMD)
57	TBP	NM_003194	1921	277-822	HKG	TATA box binding protein (Low abundant HKG)
