# Supplementary material for: Identification of Pathways Mediating Growth Differentiation Factor5-Induced Tenogenic Differentiation in Human Bone Marrow Stromal Cells
Source: PLoS One. 2015 Nov 3;10(11):e0140869. doi: 10.1371/journal.pone.0140869 (PMC4631504; doi:10.1371/journal.pone.0140869)
Supplement: S4 Table — (PDF) [file pone.0140869.s008.pdf]

S4 Table. Summary of total number of probe sets or genes before and after data normalization and filtering.

Probe Sets	Total
1. Estimated number of genes on array	28,869
2. Total distinct probe sets on the array	764,885
3. Positive control probe sets on the array	1,195
4. Negative control probe sets on the array	2,904
**5. Total number of probe sets detected on chip**	**33,297**
6. Total number of probe sets detected on chip but do not have a present DABG detection call	1,320
7. Total number of probe sets detected on chip after omitted control probe sets and probe sets without a present DABG detection call	27,878
8. Number of probe sets omitted after data filtering	662
**9. Total number of probe sets used to assess differentially expressed genes**	**27,216**
