# Supplementary material for: Identification of Pathways Mediating Growth Differentiation Factor5-Induced Tenogenic Differentiation in Human Bone Marrow Stromal Cells
Source: PLoS One. 2015 Nov 3;10(11):e0140869. doi: 10.1371/journal.pone.0140869 (PMC4631504; doi:10.1371/journal.pone.0140869)
Supplement: S5 Table — (PDF) [file pone.0140869.s009.pdf]

S5 Table. A summary of the number of differentially expressed probe sets.

	**Uncorrected *p*-value <0.001**		**Corrected *p*-value<0.05**	
	Log-ratio < -1	Log-ratio > 1	Log-ratio < -1	Log-ratio > 1
**Group 1 vs 4**	168	159	204	182
**Group 2 vs 4**	211	185	268	212
**Group 3 vs 4**	324	264	400	291
**Group 1 vs 3**	139	98	152	119
**Group 2 vs 3**	50	8	50	8
**Group 1 vs 2**	19	22	12	19

(Group 1: Control hMSC, Group 2: Day-4 GDF5-induced hMSC, Group 3: Day-10 GDF5-induced hMSC, Group 4: tenocytes)
