# Supplementary material for: Identification of Pathways Mediating Growth Differentiation Factor5-Induced Tenogenic Differentiation in Human Bone Marrow Stromal Cells
Source: PLoS One. 2015 Nov 3;10(11):e0140869. doi: 10.1371/journal.pone.0140869 (PMC4631504; doi:10.1371/journal.pone.0140869)
Supplement: S6 Table — (PDF) [file pone.0140869.s010.pdf]

S6 Table. The most significantly altered genes in the GDF5-induced hMSC and tenocytes [LR: log2 ratio, p-value (corr): corrected p-value].

Gene Symbol	Description	Day 4 GDF5-induction (G2 vs G1)		Day 10 GDF5-induction (G3 vs G1)		Tenocytes (G4 vs G1)		Day 10 vs Day 4 GDF5-induction (G3 vs G2)		Tenocytes vs Day 4 GDF5-Induction (G4 vs G2)		Tenocytes vs Day 10 GDF5-Induction (G4 vs G3)	
		LR	p-value (corr)	LR	p-value (corr)	LR	p-value (corr)	LR	p-value (corr)	LR	p-value (corr)	LR	p-value (corr)
**Most up-regulated in day 4 GDF5-induced hMSCs**													
CMKLR1	chemokine-like receptor 1	**1.99**	**1.08E-02**	2.49	1.12E-04	-0.39	6.87E-01	0.50	6.21E-01	-2.38	3.77E-04	-2.88	2.59E-05
FADS2	fatty acid desaturase 2	**1.71**	**2.38E-02**	1.75	2.40E-03	0.83	2.37E-01	0.05	9.79E-01	-0.88	1.91E-01	-0.93	1.36E-01
ACAT2	acetyl-CoA acetyltransferase 2	**1.37**	**2.41E-02**	1.16	1.16E-02	0.82	1.19E-01	-0.21	8.23E-01	-0.55	3.58E-01	-0.33	6.28E-01
**CCL2**	**chemokine (C-C motif) ligand 2**	**1.35**	**4.35E-03**	2.07	3.34E-06	1.82	3.89E-05	0.73	1.02E-01	0.48	2.94E-01	-0.25	6.52E-01
PIK3R3	phosphoinositide-3-kinase, regulatory subunit 3 (gamma)	**1.33**	**5.42E-03**	1.43	2.63E-04	0.74	7.20E-02	0.09	9.20E-01	-0.59	1.68E-01	-0.68	7.84E-02
SC4MOL	sterol-C4-methyl oxidase-like	**1.30**	**1.28E-02**	1.19	3.10E-03	0.36	5.41E-01	-0.11	9.13E-01	-0.94	3.01E-02	-0.83	4.46E-02
FDPS	farnesyl diphosphate synthase	**1.24**	**3.76E-02**	1.02	2.11E-02	0.06	9.54E-01	-0.22	8.08E-01	-1.17	1.42E-02	-0.96	3.66E-02
TIPARP	TCDD-inducible poly(ADP-ribose) polymerase	**1.11**	**3.40E-03**	1.09	4.00E-04	0.23	6.00E-01	-0.02	9.82E-01	-0.87	5.99E-03	-0.85	5.03E-03
SQLE	squalene epoxidase	**1.10**	**1.02E-02**	1.42	7.32E-05	0.55	1.49E-01	0.32	5.51E-01	-0.55	1.36E-01	-0.87	9.22E-03
**ARHGAP29**	**Rho GTPase activating protein 29**	**1.10**	**1.30E-02**	1.03	2.66E-03	1.94	6.10E-06	-0.07	9.36E-01	0.85	2.10E-02	0.92	8.74E-03
**Most up-regulated in day 10 GDF5-induced hMSCs**													
MX1	myxovirus (influenza virus) resistance 1, interferon-inducible protein p78	0.18	9.58E-01	**3.35**	**5.01E-04**	0.22	9.19E-01	3.17	3.50E-03	0.04	9.88E-01	-3.14	1.29E-03
HERC6	hect domain and RLD 6	0.15	9.49E-01	**3.02**	**3.10E-05**	0.09	9.55E-01	2.87	3.81E-04	-0.06	9.65E-01	-2.93	5.22E-05
OAS1	2',5'-oligoadenylate synthetase 1, 40/46kDa	0.39	8.69E-01	**2.99**	**4.91E-04**	1.68	6.76E-02	2.60	7.05E-03	1.29	1.82E-01	-1.31	1.48E-01
CCL26	chemokine (C-C motif) ligand 26	-0.09	9.60E-01	**2.89**	**2.22E-06**	-0.14	8.94E-01	2.98	1.36E-05	-0.05	9.64E-01	-3.03	1.06E-06
KRT14	keratin 14	0.90	4.67E-01	**2.87**	**1.31E-04**	0.47	6.74E-01	1.97	1.39E-02	-0.43	7.09E-01	-2.40	1.08E-03
TNFAIP6	tumor necrosis factor, alpha-induced protein 6	0.19	9.24E-01	**2.68**	**5.39E-05**	1.69	9.61E-03	2.49	6.98E-04	1.50	2.01E-02	-0.99	1.23E-01
IFIT1	interferon-induced protein with tetratricopeptide repeats 1	-0.04	9.93E-01	**2.58**	**7.82E-03**	0.28	8.96E-01	2.62	2.00E-02	0.32	8.67E-01	-2.30	2.16E-02
HTR2A	5-hydroxytryptamine (serotonin) receptor 2A	0.43	8.43E-01	**2.58**	**1.73E-03**	-1.31	1.69E-01	2.14	2.21E-02	-1.75	4.85E-02	-3.89	2.91E-05
C1QTNF3	C1q and tumor necrosis factor related protein 3	0.93	2.39E-01	**2.58**	**1.66E-05**	0.37	6.50E-01	1.64	6.03E-03	-0.56	4.09E-01	-2.21	1.07E-04
OAS2	2'-5'-oligoadenylate synthetase 2, 69/71kDa	0.50	6.06E-01	**2.57**	**8.22E-06**	-0.35	6.42E-01	2.07	5.90E-04	-0.86	1.20E-01	-2.92	1.78E-06
**Most up-regulated in tenocytes**													
RPS4Y1	ribosomal protein S4, Y-linked 1	-0.09	9.79E-01	-0.04	9.83E-01	**4.64**	**2.26E-05**	0.05	9.86E-01	4.73	1.23E-05	4.68	9.54E-06
EIF1AY	eukaryotic translation initiation factor 1A, Y-linked	0.02	9.97E-01	0.02	9.92E-01	**4.49**	**2.58E-05**	0.00	1.00E+00	4.47	1.98E-05	4.47	1.32E-05

DDX3Y	DEAD (Asp-Glu-Ala-Asp) box polypeptide 3, Y-linked	0.14	9.62E-01	0.12	9.39E-01	**4.24**	**3.45E-05**	-0.02	9.94E-01	4.10	4.14E-05	4.12	2.34E-05
RBP4	retinol binding protein 4, plasma	0.21	8.40E-01	0.37	4.26E-01	**4.23**	**1.95E-10**	0.16	8.42E-01	4.03	3.89E-10	3.86	9.62E-10
THBS4	thrombospondin 4	0.05	9.89E-01	0.30	8.11E-01	**4.04**	**2.16E-05**	0.26	8.85E-01	3.99	1.65E-05	3.73	2.54E-05
FGL2	fibrinogen-like 2	-0.49	9.01E-01	2.42	5.03E-02	**3.96**	**3.59E-03**	2.91	4.20E-02	4.45	1.03E-03	1.54	2.89E-01
PRG4	proteoglycan 4	0.25	9.62E-01	-0.89	6.73E-01	**3.89**	**1.47E-02**	-1.14	6.56E-01	3.64	2.06E-02	4.78	1.69E-03
OGN	osteoglycin	1.56	4.92E-01	1.36	3.47E-01	**3.57**	**8.85E-03**	-0.20	9.56E-01	2.01	1.64E-01	2.21	9.50E-02
MEOX2	mesenchyme homeobox 2	0.97	4.17E-01	0.54	5.63E-01	**3.51**	**3.30E-05**	-0.44	7.39E-01	2.54	9.87E-04	2.98	1.16E-04
CLGN	calmegin	-0.14	9.51E-01	-0.70	3.19E-01	**3.47**	**6.42E-06**	-0.56	5.81E-01	3.61	3.27E-06	4.17	2.39E-07
**Most down-regulated in day 4 GDF5-induced hMSCs**													
**NEFM**	**neurofilament, medium polypeptide**	**-2.23**	**3.53E-02**	-2.55	1.45E-03	-2.44	5.04E-03	-0.32	8.51E-01	-0.22	8.94E-01	0.11	9.52E-01
**ITGA8**	**integrin, alpha 8**	**-1.86**	**5.42E-03**	-2.99	2.63E-06	-3.06	3.72E-06	-1.14	6.34E-02	-1.20	3.13E-02	-0.06	9.57E-01
HMOX1	heme oxygenase (decycling) 1	**-1.73**	**1.89E-02**	-2.21	2.12E-04	0.57	4.61E-01	-0.48	6.10E-01	2.30	2.73E-04	2.78	1.84E-05
**PODXL**	**podocalyxin-like**	**-1.69**	**1.26E-02**	-1.92	4.07E-04	2.01	6.39E-04	-0.23	8.37E-01	3.70	1.91E-07	3.93	5.75E-08
ID3	inhibitor of DNA binding 3, dominant negative helix-loop-helix protein	**-1.57**	**9.01E-05**	-1.38	5.62E-05	0.07	9.21E-01	0.19	7.42E-01	1.64	1.22E-05	1.45	3.74E-05
**STC1**	**stanniocalcin 1**	**-1.49**	**1.17E-02**	-1.45	1.58E-03	-1.07	3.12E-02	0.04	9.75E-01	0.42	5.09E-01	0.38	5.43E-01
**ACAN**	**aggrecan**	**-1.46**	**1.03E-02**	-2.22	1.32E-05	-1.37	4.24E-03	-0.76	1.65E-01	0.09	9.24E-01	0.85	6.15E-02
TFRC	transferrin receptor (p90, CD71)	**-1.45**	**1.22E-03**	-0.76	2.96E-02	-0.49	2.52E-01	0.69	1.10E-01	0.96	1.17E-02	0.27	5.95E-01
MT2A	metallothionein 2A	**-1.34**	**2.05E-05**	-0.79	8.36E-04	0.37	1.81E-01	0.55	4.21E-02	1.71	1.38E-07	1.16	1.71E-05
**C7orf69**	**chromosome 7 open reading frame 69**	**-1.32**	**3.08E-02**	-1.26	5.29E-03	-1.57	2.02E-03	0.05	9.69E-01	-0.26	7.39E-01	-0.31	6.57E-01
**Most down-regulated in day 10 GDF5-induced hMSCs**													
SLC14A1	solute carrier family 14 (urea transporter), member 1 (Kidd blood group)	-0.03	9.95E-01	**-3.03**	**4.19E-04**	-1.38	1.50E-01	-3.00	2.34E-03	-1.35	1.53E-01	1.65	5.37E-02
**ITGA8**	**integrin, alpha 8**	-1.86	5.42E-03	**-2.99**	**2.63E-06**	-3.06	3.72E-06	-1.14	6.34E-02	-1.20	3.13E-02	-0.06	9.57E-01
DLGAP5	discs, large (Drosophila) homolog-associated protein 5	-1.80	1.56E-01	**-2.99**	**8.20E-04**	-0.02	9.94E-01	-1.19	3.23E-01	1.78	6.28E-02	2.97	1.18E-03
IL7R	interleukin 7 receptor	-2.08	8.20E-02	**-2.74**	**1.51E-03**	-2.08	2.62E-02	-0.66	6.58E-01	0.00	9.99E-01	0.67	5.87E-01
ANLN	anillin, actin binding protein	-1.61	2.29E-01	**-2.68**	**2.37E-03**	0.17	9.35E-01	-1.07	4.01E-01	1.78	6.39E-02	2.84	1.81E-03
**NEFM**	**neurofilament, medium polypeptide**	-2.23	3.53E-02	**-2.55**	**1.45E-03**	-2.44	5.04E-03	-0.32	8.51E-01	-0.22	8.94E-01	0.11	9.52E-01
MKI67	antigen identified by monoclonal antibody Ki-67	-1.80	1.29E-01	**-2.55**	**2.05E-03**	0.49	7.22E-01	-0.75	5.79E-01	2.29	9.78E-03	3.04	5.19E-04
KIF20A	kinesin family member 20A	-1.44	2.41E-01	**-2.55**	**1.60E-03**	0.09	9.65E-01	-1.11	3.09E-01	1.52	8.52E-02	2.63	1.57E-03
TOP2A	topoisomerase (DNA) II alpha 170kDa	-1.32	2.65E-01	**-2.42**	**1.61E-03**	0.38	7.82E-01	-1.10	2.80E-01	1.70	3.82E-02	2.80	5.47E-04
ASPM	asp (abnormal spindle) homolog, microcephaly associated (Drosophila)	-1.19	3.42E-01	**-2.37**	**2.26E-03**	0.77	4.74E-01	-1.18	2.42E-01	1.96	1.83E-02	3.14	1.93E-04
**Most down-regulated in tenocytes**													
SRGN	serglycin	-0.12	9.64E-01	-0.40	7.22E-01	**-4.41**	**3.72E-06**	-0.28	8.64E-01	-4.29	4.22E-06	-4.01	6.81E-06

VCAM1	vascular cell adhesion molecule 1	0.54	7.83E-01	0.83	3.85E-01	**-3.92**	**5.05E-05**	0.29	8.74E-01	-4.46	7.02E-06	-4.75	2.30E-06
ANK3	ankyrin 3, node of Ranvier (ankyrin G)	0.42	6.51E-01	0.46	3.99E-01	**-3.59**	**4.09E-08**	0.04	9.80E-01	-4.01	4.57E-09	-4.05	3.80E-09
ANKRD1	ankyrin repeat domain 1 (cardiac muscle)	-1.02	6.25E-01	0.03	9.90E-01	**-3.41**	**2.81E-03**	1.05	5.19E-01	-2.39	3.12E-02	-3.44	1.37E-03
DEPDC6	DEP domain containing 6	0.15	9.62E-01	0.04	9.84E-01	**-3.35**	**7.16E-04**	-0.11	9.65E-01	-3.49	3.80E-04	-3.38	3.44E-04
PDE5A	phosphodiesterase 5A, cGMP-specific	-0.24	8.85E-01	-1.44	8.82E-03	**-3.31**	**3.72E-06**	-1.19	7.04E-02	-3.07	7.02E-06	-1.87	1.26E-03
**ITGA8**	**integrin, alpha 8**	-1.86	5.42E-03	-2.99	2.63E-06	**-3.06**	**3.72E-06**	-1.14	6.34E-02	-1.20	3.13E-02	-0.06	9.57E-01
TSPAN18	tetraspanin 18	-0.05	9.87E-01	-1.49	5.82E-02	**-3.05**	**5.65E-04**	-1.43	1.35E-01	-3.00	5.37E-04	-1.57	5.16E-02
SERPINB2	serpin peptidase inhibitor, clade B (ovalbumin), member 2	-0.01	9.98E-01	1.32	2.39E-01	**-2.95**	**8.21E-03**	1.34	3.51E-01	-2.94	7.69E-03	-4.28	1.42E-04
RAB27B	RAB27B, member RAS oncogene family	-0.83	6.60E-01	-1.14	2.61E-01	**-2.84**	**4.80E-03**	-0.31	8.83E-01	-2.00	4.30E-02	-1.70	7.62E-02
**Co-expressed genes (Up- or down-regulated in all day 4, 10 GDF5-induced hMSCs and tenocytes)**													
ITGA8	integrin, alpha 8	-1.86	5.42E-03	-2.99	2.63E-06	-3.06	3.72E-06	-1.14	6.34E-02	-1.20	3.13E-02	-0.06	9.57E-01
NEFM	neurofilament, medium polypeptide	-2.23	3.53E-02	-2.55	1.45E-03	-2.44	5.04E-03	-0.32	8.51E-01	-0.22	8.94E-01	0.11	9.52E-01
C7orf69	chromosome 7 open reading frame 69	-1.32	3.08E-02	-1.26	5.29E-03	-1.57	2.02E-03	0.05	9.69E-01	-0.26	7.39E-01	-0.31	6.57E-01
ACAN	aggrecan	-1.46	1.03E-02	-2.22	1.32E-05	-1.37	4.24E-03	-0.76	1.65E-01	0.09	9.24E-01	0.85	6.15E-02
STC1	stanniocalcin 1	-1.49	1.17E-02	-1.45	1.58E-03	-1.07	3.12E-02	0.04	9.75E-01	0.42	5.09E-01	0.38	5.43E-01
ENO2	enolase 2 (gamma, neuronal)	-1.30	2.22E-03	-1.69	1.32E-05	-1.02	5.41E-03	-0.39	4.17E-01	0.28	5.63E-01	0.68	4.84E-02
CCL2	chemokine (C-C motif) ligand 2	1.35	4.35E-03	2.07	3.34E-06	1.82	3.89E-05	0.73	1.02E-01	0.48	2.94E-01	-0.25	6.52E-01
ARHGAP29	Rho GTPase activating protein 29	1.10	1.30E-02	1.03	2.66E-03	1.94	6.10E-06	-0.07	9.36E-01	0.85	2.10E-02	0.92	8.74E-03
PODXL	podocalyxin-like	-1.69	1.26E-02	-1.92	4.07E-04	2.01	6.39E-04	-0.23	8.37E-01	3.70	1.91E-07	3.93	5.75E-08
