# Supplementary material for: Identification of Pathways Mediating Growth Differentiation Factor5-Induced Tenogenic Differentiation in Human Bone Marrow Stromal Cells
Source: PLoS One. 2015 Nov 3;10(11):e0140869. doi: 10.1371/journal.pone.0140869 (PMC4631504; doi:10.1371/journal.pone.0140869)
Supplement: S7 Table — (PDF) [file pone.0140869.s011.pdf]

S7 Table. List of Genes Modulated in hMSCs by GDF5 Treatment and Genes Modulated in Tenocytes (Total: 873 genes)

Gene Symbol	Day 4 GDF5-induction (G2 vs G1)		Day 10 GDF5-induction (G3 vs G1)		Tenocytes (G4 vs G1)		Day 10 vs Day 4 GDF5-induction (G3 vs G2)		Tenocytes vs Day 4 GDF5-Induction (G4 vs G2)		Tenocytes vs Day 10 GDF5-Induction (G4 vs G3)	
	LR	p-value (corr)	LR	p-value (corr)	LR	p-value (corr)	LR	p-value (corr)	LR	p-value (corr)	LR	p-value (corr)
ABCA8	-0.28	8.16E-01	-0.56	2.92E-01	-2.02	2.28E-04	-0.28	7.53E-01	-1.74	8.87E-04	-1.46	2.94E-03
ABCC9	-0.75	4.16E-01	-1.24	1.95E-02	-1.10	6.60E-02	-0.50	5.77E-01	-0.35	6.88E-01	0.15	8.88E-01
ACAN	-1.46	1.03E-02	-2.22	1.32E-05	-1.37	4.24E-03	-0.76	1.65E-01	0.09	9.24E-01	0.85	6.15E-02
ACAT2	1.37	2.41E-02	1.16	1.16E-02	0.82	1.19E-01	-0.21	8.23E-01	-0.55	3.58E-01	-0.33	6.28E-01
ACSS3	-0.08	9.58E-01	-0.65	1.18E-01	-1.87	8.53E-05	-0.58	2.88E-01	-1.79	1.11E-04	-1.22	3.43E-03
ACTA2	0.23	7.63E-01	0.00	9.98E-01	-2.07	9.38E-07	-0.23	6.96E-01	-2.30	1.64E-07	-2.07	5.28E-07
ACTA2	0.40	6.14E-01	-0.12	8.63E-01	-1.01	1.90E-02	-0.52	3.43E-01	-1.41	1.18E-03	-0.89	2.80E-02
ACTC1	-0.69	7.10E-01	-0.81	4.32E-01	1.41	1.48E-01	-0.12	9.60E-01	2.10	2.04E-02	2.22	9.97E-03
ACVR2A	0.95	2.51E-01	1.15	3.03E-02	-2.16	4.06E-04	0.20	8.65E-01	-3.11	3.65E-06	-3.31	1.07E-06
ADAM23	0.36	8.14E-01	0.69	3.18E-01	2.33	6.20E-04	0.33	7.82E-01	1.98	2.56E-03	1.65	7.72E-03
ADAMTS12	0.51	6.57E-01	1.51	5.95E-03	-0.17	8.80E-01	1.00	1.45E-01	-0.68	3.32E-01	-1.68	3.33E-03
ADAMTS2	-0.02	9.89E-01	0.22	6.73E-01	-0.93	1.98E-02	0.25	7.13E-01	-0.91	2.12E-02	-1.15	2.40E-03
ADAMTS9	0.67	8.75E-03	0.99	1.40E-05	-0.48	2.38E-02	0.32	1.98E-01	-1.15	3.27E-06	-1.48	5.75E-08
ADAMTSL1	-0.28	6.63E-01	-0.75	1.24E-02	0.61	7.02E-02	-0.48	2.26E-01	0.89	6.89E-03	1.37	6.15E-05
ADAMTSL1	-0.19	8.51E-01	-1.02	5.53E-03	0.74	7.38E-02	-0.83	5.91E-02	0.93	2.00E-02	1.75	3.21E-05
ADAMTSL3	-0.01	9.93E-01	0.03	9.42E-01	1.88	4.09E-08	0.04	9.45E-01	1.89	3.05E-08	1.84	3.67E-08
AGPAT9	0.08	9.24E-01	-0.03	9.55E-01	1.02	7.16E-04	-0.11	8.43E-01	0.94	1.44E-03	1.05	2.83E-04
AHNAK2	0.12	9.67E-01	-0.31	8.08E-01	1.89	2.65E-02	-0.43	7.79E-01	1.77	3.53E-02	2.21	6.03E-03
AHR	0.33	3.21E-01	0.24	3.12E-01	-0.70	2.38E-03	-0.09	8.39E-01	-1.03	2.78E-05	-0.94	5.57E-05
AIM1	0.25	7.80E-01	1.03	5.65E-03	2.23	3.72E-06	0.78	8.41E-02	1.98	1.31E-05	1.20	2.14E-03
AK5	-0.41	7.95E-01	-0.75	3.09E-01	0.83	3.11E-01	-0.34	7.94E-01	1.24	8.25E-02	1.58	1.69E-02
AKAP6	-0.50	5.68E-01	-0.61	2.08E-01	0.97	5.14E-02	-0.11	9.19E-01	1.47	2.69E-03	1.58	8.78E-04
ALDH18A1	0.18	7.48E-01	0.65	6.91E-03	-0.37	1.97E-01	0.47	1.12E-01	-0.55	3.54E-02	-1.02	1.34E-04
ALDH1B1	0.38	6.29E-01	0.23	6.74E-01	-0.79	6.34E-02	-0.14	8.68E-01	-1.17	4.85E-03	-1.02	9.23E-03
ALDOC	-0.52	1.89E-01	-1.04	2.24E-04	-0.80	6.40E-03	-0.52	1.05E-01	-0.28	4.46E-01	0.25	4.81E-01
ALPK2	0.10	9.56E-01	0.26	7.06E-01	-1.19	1.85E-02	0.16	8.77E-01	-1.29	1.02E-02	-1.45	2.54E-03
AMIGO2	-1.13	1.16E-01	-0.32	6.52E-01	-1.50	6.40E-03	0.81	2.12E-01	-0.37	6.36E-01	-1.18	2.20E-02

ANGPTL1	0.94	2.96E-01	2.39	9.68E-05	0.22	8.46E-01	1.45	2.61E-02	-0.72	3.10E-01	-2.17	3.61E-04
ANGPTL2	0.08	9.76E-01	-0.35	7.14E-01	1.45	3.98E-02	-0.43	7.18E-01	1.37	4.90E-02	1.80	6.40E-03
ANGPTL5	-0.03	9.94E-01	-0.23	8.51E-01	2.85	3.26E-04	-0.20	9.04E-01	2.88	2.29E-04	3.08	6.63E-05
ANGPTL7	0.06	9.90E-01	-0.09	9.60E-01	2.45	1.74E-02	-0.15	9.56E-01	2.40	1.86E-02	2.54	8.58E-03
ANK3	0.42	6.51E-01	0.46	3.99E-01	-3.59	4.09E-08	0.04	9.80E-01	-4.01	4.57E-09	-4.05	3.80E-09
ANKFN1	-0.03	9.88E-01	-0.34	5.49E-01	-1.04	2.61E-02	-0.31	6.88E-01	-1.02	2.79E-02	-0.71	1.26E-01
ANKRD1	-1.02	6.25E-01	0.03	9.90E-01	-3.41	2.81E-03	1.05	5.19E-01	-2.39	3.12E-02	-3.44	1.37E-03
ANKRD37	-0.79	2.18E-01	-0.70	1.10E-01	-1.27	5.98E-03	0.09	9.34E-01	-0.48	3.80E-01	-0.57	2.37E-01
ANKRD5	0.07	9.62E-01	-0.30	5.77E-01	-0.94	3.36E-02	-0.37	5.82E-01	-1.01	2.06E-02	-0.64	1.48E-01
ANLN	-1.61	2.29E-01	-2.68	2.37E-03	0.17	9.35E-01	-1.07	4.01E-01	1.78	6.39E-02	2.84	1.81E-03
ANXA3	0.04	9.78E-01	0.13	8.50E-01	1.26	4.30E-03	0.09	9.32E-01	1.22	4.89E-03	1.13	6.18E-03
AOX1	-0.42	7.90E-01	-0.97	1.62E-01	0.73	4.05E-01	-0.55	6.18E-01	1.15	1.18E-01	1.70	1.10E-02
AP1S2	-0.30	6.10E-01	-0.46	1.37E-01	1.24	2.55E-04	-0.16	7.83E-01	1.54	1.35E-05	1.70	2.89E-06
AP3M2	-0.29	6.78E-01	-0.40	2.69E-01	-1.05	3.76E-03	-0.12	8.78E-01	-0.76	3.16E-02	-0.64	5.81E-02
APOBEC3B	-0.57	3.33E-01	-1.11	2.36E-03	-0.43	3.60E-01	-0.54	2.58E-01	0.13	8.46E-01	0.67	7.51E-02
APOL1	-0.14	9.24E-01	1.37	2.43E-03	-2.03	1.18E-04	1.51	3.73E-03	-1.89	2.12E-04	-3.40	5.75E-08
ARHGAP11A	-0.68	5.28E-01	-1.69	3.27E-03	0.82	2.42E-01	-1.01	1.59E-01	1.50	1.53E-02	2.51	8.76E-05
ARHGAP11B	-0.72	2.76E-01	-1.04	1.19E-02	0.32	6.13E-01	-0.32	6.60E-01	1.04	2.06E-02	1.36	1.74E-03
ARHGAP28	1.36	1.42E-01	1.22	5.77E-02	-0.43	6.72E-01	-0.14	9.32E-01	-1.80	9.33E-03	-1.66	1.11E-02
ARHGAP29	1.10	1.30E-02	1.03	2.66E-03	1.94	6.10E-06	-0.07	9.36E-01	0.85	2.10E-02	0.92	8.74E-03
ARL15	0.24	7.34E-01	0.58	7.01E-02	-0.55	1.31E-01	0.34	4.93E-01	-0.79	2.00E-02	-1.13	7.57E-04
ARL4C	-0.07	9.74E-01	0.58	3.71E-01	-1.15	5.78E-02	0.64	4.22E-01	-1.08	7.30E-02	-1.72	2.40E-03
ARMC4	-0.15	9.22E-01	-0.04	9.65E-01	1.36	5.80E-03	0.11	9.26E-01	1.51	2.12E-03	1.40	2.63E-03
ARRDC3	0.06	9.59E-01	-0.48	1.67E-01	0.82	2.10E-02	-0.54	1.94E-01	0.76	3.07E-02	1.30	2.43E-04
ARRDC4	0.44	3.04E-01	0.40	1.62E-01	-1.27	7.96E-05	-0.04	9.60E-01	-1.72	1.30E-06	-1.68	1.18E-06
AS3MT	-0.41	4.16E-01	-1.07	4.05E-04	-0.48	1.53E-01	-0.67	5.07E-02	-0.08	8.95E-01	0.59	5.07E-02
ASPM	-1.19	3.42E-01	-2.37	2.26E-03	0.77	4.74E-01	-1.18	2.42E-01	1.96	1.83E-02	3.14	1.93E-04
ATAD2	-0.27	8.28E-01	-0.66	1.93E-01	0.36	6.20E-01	-0.39	6.20E-01	0.63	2.79E-01	1.02	3.52E-02
ATAD5	-0.24	8.69E-01	-0.35	6.19E-01	0.96	9.49E-02	-0.11	9.34E-01	1.20	2.91E-02	1.31	1.21E-02
ATP13A3	-0.42	3.10E-01	-0.30	3.06E-01	-1.17	1.29E-04	0.12	8.31E-01	-0.75	7.63E-03	-0.87	1.45E-03
ATP1B1	-0.01	9.98E-01	0.41	5.23E-01	-0.60	3.42E-01	0.41	6.16E-01	-0.60	3.40E-01	-1.01	4.63E-02
AURKA	-1.13	4.19E-01	-1.82	2.47E-02	0.09	9.64E-01	-0.69	6.13E-01	1.22	2.05E-01	1.91	2.20E-02
B3GALT2	0.16	9.24E-01	-0.88	9.83E-02	-1.40	1.24E-02	-1.04	9.22E-02	-1.56	4.85E-03	-0.52	4.26E-01
B4GALT5	0.13	9.09E-01	0.12	8.38E-01	1.15	2.28E-03	-0.01	9.94E-01	1.03	5.11E-03	1.03	3.25E-03

BARD1	-0.28	7.09E-01	-0.95	5.05E-03	0.66	8.54E-02	-0.67	1.06E-01	0.94	1.10E-02	1.60	3.47E-05
BAZ1A	-0.12	8.20E-01	-0.02	9.62E-01	0.97	1.12E-04	0.10	8.12E-01	1.09	1.81E-05	0.99	4.08E-05
BDKRB1	-0.85	4.91E-01	-1.75	9.28E-03	1.29	9.07E-02	-0.90	3.43E-01	2.14	3.58E-03	3.04	6.46E-05
BDKRB2	0.07	9.71E-01	-0.63	2.65E-01	0.51	4.71E-01	-0.70	3.16E-01	0.44	5.52E-01	1.15	2.87E-02
BDNF	-0.60	1.39E-01	-0.99	5.67E-04	-1.03	1.02E-03	-0.39	3.01E-01	-0.43	1.86E-01	-0.04	9.50E-01
BEND6	0.83	1.42E-01	0.66	1.02E-01	1.36	1.75E-03	-0.17	8.36E-01	0.53	2.62E-01	0.70	8.63E-02
BEX1	0.31	9.24E-01	0.44	7.72E-01	-2.47	1.74E-02	0.13	9.60E-01	-2.78	6.87E-03	-2.91	3.16E-03
BHMT2	0.57	5.42E-01	0.90	6.80E-02	-1.36	1.07E-02	0.34	7.05E-01	-1.92	4.25E-04	-2.26	4.19E-05
BIRC3	0.44	5.70E-01	0.13	8.54E-01	1.24	4.47E-03	-0.31	6.57E-01	0.80	6.41E-02	1.11	6.36E-03
BIRC5	-1.28	3.75E-01	-2.09	1.45E-02	0.05	9.81E-01	-0.82	5.65E-01	1.33	1.89E-01	2.15	1.53E-02
BNC1	-0.05	9.68E-01	0.48	1.72E-01	1.20	1.27E-03	0.53	2.12E-01	1.25	6.75E-04	0.72	3.52E-02
BPGM	0.26	5.75E-01	0.63	7.62E-03	-0.61	1.82E-02	0.37	2.34E-01	-0.87	9.21E-04	-1.24	1.01E-05
BRCA2	-0.36	8.18E-01	0.20	8.54E-01	1.08	1.26E-01	0.56	5.82E-01	1.45	3.02E-02	0.88	2.05E-01
BST2	0.02	9.93E-01	1.00	2.60E-03	-0.20	7.27E-01	0.99	9.42E-03	-0.21	6.95E-01	-1.20	6.78E-04
BTN3A3	0.30	7.02E-01	0.74	3.73E-02	-0.30	5.64E-01	0.44	3.86E-01	-0.60	1.40E-01	-1.04	4.50E-03
BUB1B	-1.37	3.39E-01	-1.99	2.28E-02	0.40	8.05E-01	-0.62	6.96E-01	1.77	6.70E-02	2.39	8.10E-03
C10orf116	-0.15	7.74E-01	-0.37	1.11E-01	0.74	2.80E-03	-0.21	5.54E-01	0.89	3.54E-04	1.11	1.76E-05
C11orf87	0.45	5.47E-01	0.37	4.46E-01	-0.98	2.34E-02	-0.08	9.33E-01	-1.43	1.03E-03	-1.35	1.15E-03
C13orf33	0.79	1.33E-01	0.46	2.56E-01	1.22	2.51E-03	-0.34	5.80E-01	0.43	3.57E-01	0.77	4.11E-02
C14orf106	-0.34	4.84E-01	-0.29	3.50E-01	0.87	3.13E-03	0.05	9.47E-01	1.21	8.71E-05	1.16	8.89E-05
C14orf145	-0.18	9.03E-01	-0.47	4.04E-01	0.75	1.73E-01	-0.29	7.45E-01	0.93	7.04E-02	1.22	1.09E-02
C16orf87	0.32	7.02E-01	0.76	4.94E-02	-0.44	3.91E-01	0.44	4.42E-01	-0.76	7.30E-02	-1.20	2.64E-03
C18orf54	0.32	7.51E-01	-0.16	8.26E-01	0.87	7.15E-02	-0.48	4.68E-01	0.55	3.12E-01	1.03	2.04E-02
C1orf21	-0.02	9.88E-01	-0.78	1.27E-02	0.55	1.23E-01	-0.76	3.90E-02	0.58	1.03E-01	1.33	1.29E-04
C1orf54	-0.14	9.00E-01	-1.28	4.53E-04	-2.33	6.61E-07	-1.14	5.58E-03	-2.19	1.52E-06	-1.05	3.78E-03
C1QTNF3	0.93	2.39E-01	2.58	1.66E-05	0.37	6.50E-01	1.64	6.03E-03	-0.56	4.09E-01	-2.21	1.07E-04
C21orf7	0.52	5.59E-01	0.33	6.06E-01	-1.31	9.01E-03	-0.19	8.46E-01	-1.83	3.85E-04	-1.64	7.89E-04
C3	0.98	5.49E-01	0.78	4.65E-01	2.20	1.83E-02	-0.20	9.26E-01	1.22	2.33E-01	1.43	1.21E-01
C4orf31	0.54	7.83E-01	0.01	9.98E-01	-2.66	3.17E-03	-0.54	7.18E-01	-3.20	4.25E-04	-2.66	1.67E-03
C5	0.20	8.15E-01	-0.65	5.81E-02	0.82	2.80E-02	-0.86	3.00E-02	0.62	1.07E-01	1.47	1.01E-04
C5orf13	0.36	6.93E-01	0.35	5.27E-01	-0.73	1.36E-01	-0.01	9.91E-01	-1.09	1.71E-02	-1.08	1.32E-02
C5orf30	0.11	9.38E-01	0.02	9.81E-01	1.24	4.41E-03	-0.09	9.32E-01	1.14	7.91E-03	1.22	2.89E-03
C5orf36	0.66	9.56E-02	0.93	1.08E-03	-0.11	8.48E-01	0.28	5.42E-01	-0.76	1.15E-02	-1.04	5.30E-04
C5orf36	0.62	1.51E-01	1.01	8.69E-04	-0.10	8.72E-01	0.39	3.51E-01	-0.72	2.43E-02	-1.10	4.92E-04

C6orf138	1.05	1.46E-01	0.56	3.24E-01	-1.25	2.08E-02	-0.49	5.42E-01	-2.30	6.99E-05	-1.82	6.06E-04
C7orf58	-0.13	9.68E-01	-0.32	8.40E-01	1.58	1.36E-01	-0.19	9.37E-01	1.71	9.47E-02	1.90	4.53E-02
C7orf69	-1.32	3.08E-02	-1.26	5.29E-03	-1.57	2.02E-03	0.05	9.69E-01	-0.26	7.39E-01	-0.31	6.57E-01
C8orf84	0.00	9.99E-01	-0.17	8.81E-01	-1.52	2.34E-02	-0.17	9.11E-01	-1.52	2.12E-02	-1.35	3.18E-02
C9orf21	0.03	9.78E-01	0.50	5.73E-02	1.09	2.75E-04	0.47	1.44E-01	1.06	2.90E-04	0.59	2.63E-02
CAB39L	0.81	2.29E-01	1.06	1.43E-02	0.49	4.04E-01	0.26	7.58E-01	-0.32	6.41E-01	-0.57	2.59E-01
CACHD1	-0.25	8.02E-01	-0.07	9.30E-01	1.07	1.53E-02	0.19	8.25E-01	1.33	2.64E-03	1.14	6.38E-03
CACNB2	0.19	8.67E-01	-0.05	9.51E-01	1.25	3.24E-03	-0.23	7.50E-01	1.07	1.01E-02	1.30	1.24E-03
CACNB4	0.44	7.29E-01	1.32	1.73E-02	1.13	6.92E-02	0.88	2.23E-01	0.69	3.28E-01	-0.19	8.52E-01
CADM1	0.14	9.26E-01	1.03	1.86E-02	-0.11	9.07E-01	0.89	9.28E-02	-0.25	7.35E-01	-1.14	1.13E-02
CALCRL	0.56	5.43E-01	0.53	3.45E-01	-1.20	2.24E-02	-0.03	9.84E-01	-1.76	9.42E-04	-1.73	7.23E-04
CAMK2N1	0.57	5.53E-01	0.46	4.70E-01	1.42	1.03E-02	-0.12	9.27E-01	0.84	1.45E-01	0.96	6.92E-02
CAPG	-0.35	7.30E-01	-0.64	1.84E-01	1.20	1.50E-02	-0.29	7.27E-01	1.55	1.72E-03	1.84	1.94E-04
CASC5	-1.40	3.04E-01	-2.24	8.75E-03	0.45	7.67E-01	-0.84	5.49E-01	1.85	4.85E-02	2.69	2.63E-03
CBLB	-0.65	3.13E-02	-1.00	6.63E-05	-0.46	6.95E-02	-0.35	2.50E-01	0.19	5.61E-01	0.54	2.05E-02
CCBE1	-0.63	4.65E-01	-0.85	9.06E-02	0.73	2.07E-01	-0.22	8.35E-01	1.37	9.50E-03	1.58	1.82E-03
CCDC18	-0.27	6.29E-01	-0.22	5.55E-01	1.30	7.46E-05	0.06	9.37E-01	1.57	4.83E-06	1.52	5.46E-06
CCDC81	0.80	1.73E-01	0.81	4.27E-02	-0.44	4.14E-01	0.01	9.92E-01	-1.23	4.09E-03	-1.24	2.56E-03
CCL11	0.22	9.15E-01	2.00	1.65E-03	0.07	9.66E-01	1.78	1.36E-02	-0.15	9.08E-01	-1.93	2.94E-03
CCL2	1.35	4.35E-03	2.07	3.34E-06	1.82	3.89E-05	0.73	1.02E-01	0.48	2.94E-01	-0.25	6.52E-01
CCL26	-0.09	9.60E-01	2.89	2.22E-06	-0.14	8.94E-01	2.98	1.36E-05	-0.05	9.64E-01	-3.03	1.06E-06
CCNA2	-1.21	3.36E-01	-1.76	2.29E-02	0.05	9.78E-01	-0.54	7.02E-01	1.27	1.57E-01	1.81	2.30E-02
CCND2	0.45	8.30E-01	0.82	3.93E-01	-1.32	1.61E-01	0.37	8.27E-01	-1.77	4.28E-02	-2.14	9.49E-03
CD109	0.18	8.23E-01	0.05	9.31E-01	1.09	1.54E-03	-0.13	8.47E-01	0.91	5.93E-03	1.04	1.24E-03
CD200	0.40	6.76E-01	0.27	6.84E-01	-0.70	1.96E-01	-0.13	9.02E-01	-1.10	2.45E-02	-0.97	3.86E-02
CD248	0.38	2.51E-01	0.49	2.22E-02	-1.54	2.29E-07	0.11	8.08E-01	-1.92	4.57E-09	-2.02	2.28E-09
CD36	0.63	7.72E-01	0.74	5.26E-01	-2.54	9.75E-03	0.11	9.65E-01	-3.16	1.37E-03	-3.28	6.33E-04
CD55	-0.01	9.98E-01	0.02	9.89E-01	1.40	3.94E-02	0.02	9.91E-01	1.41	3.54E-02	1.39	2.93E-02
CD68	-0.48	4.82E-01	-1.29	9.02E-04	0.04	9.62E-01	-0.81	7.62E-02	0.53	2.52E-01	1.33	8.97E-04
CD9	0.19	8.89E-01	0.35	5.50E-01	1.01	3.85E-02	0.16	8.77E-01	0.82	9.93E-02	0.66	1.80E-01
CDC42EP3	-0.08	9.54E-01	-0.33	4.55E-01	-1.44	3.87E-04	-0.25	6.95E-01	-1.36	5.37E-04	-1.11	2.46E-03
CDC45	-0.46	7.03E-01	-0.55	4.07E-01	0.79	2.41E-01	-0.09	9.53E-01	1.25	3.66E-02	1.34	1.77E-02
CDC6	0.24	8.69E-01	1.03	4.35E-02	0.35	6.70E-01	0.79	2.36E-01	0.11	9.23E-01	-0.68	2.46E-01
CDCA2	-0.49	7.04E-01	-1.02	9.15E-02	0.86	2.26E-01	-0.53	5.85E-01	1.35	3.36E-02	1.88	2.11E-03

CDCA7L	-0.44	5.56E-01	-0.01	9.87E-01	0.79	6.90E-02	0.43	4.75E-01	1.23	3.59E-03	0.80	4.62E-02
CDCP1	-0.66	5.25E-01	-0.95	9.59E-02	0.20	8.54E-01	-0.29	7.92E-01	0.86	1.81E-01	1.15	4.31E-02
CDH1	0.41	5.74E-01	1.12	2.81E-03	-0.26	6.57E-01	0.71	1.20E-01	-0.67	1.10E-01	-1.38	5.37E-04
CDH6	-0.14	9.58E-01	-0.04	9.79E-01	-2.20	5.95E-03	0.10	9.60E-01	-2.06	9.07E-03	-2.16	4.09E-03
CDK1	-0.26	8.70E-01	-0.43	5.47E-01	0.83	2.06E-01	-0.17	8.93E-01	1.09	7.14E-02	1.26	2.45E-02
CDK6	0.42	5.22E-01	1.07	2.69E-03	-0.07	9.31E-01	0.65	1.42E-01	-0.50	2.48E-01	-1.14	2.06E-03
CDKN3	-1.17	2.96E-01	-2.23	1.80E-03	0.23	8.76E-01	-1.06	2.58E-01	1.40	7.29E-02	2.46	9.75E-04
CDRT1	-0.29	7.60E-01	1.00	1.29E-02	-0.66	1.69E-01	1.29	6.36E-03	-0.37	5.31E-01	-1.66	1.94E-04
CENPE	-0.64	6.38E-01	-0.91	2.05E-01	1.11	1.52E-01	-0.27	8.52E-01	1.75	1.46E-02	2.02	3.34E-03
CENPF	-1.73	1.16E-01	-2.21	3.96E-03	0.53	6.64E-01	-0.48	7.37E-01	2.26	6.37E-03	2.74	7.64E-04
CENPJ	-0.05	9.70E-01	0.49	2.68E-01	1.02	1.88E-02	0.54	3.18E-01	1.07	1.21E-02	0.53	2.50E-01
CENPK	-0.32	8.68E-01	-0.59	4.83E-01	1.34	7.12E-02	-0.27	8.51E-01	1.66	2.12E-02	1.93	5.08E-03
CENPW	-0.51	5.85E-01	-0.75	1.34E-01	0.32	6.72E-01	-0.24	8.03E-01	0.83	1.22E-01	1.07	2.80E-02
CEP135	-0.36	3.57E-01	-0.24	4.07E-01	0.77	3.84E-03	0.12	7.95E-01	1.13	6.05E-05	1.01	1.41E-04
CEP55	-1.12	3.98E-01	-1.83	1.84E-02	0.64	5.87E-01	-0.71	5.82E-01	1.75	3.84E-02	2.47	2.40E-03
CERCAM	0.09	9.03E-01	0.52	3.01E-02	-0.93	6.09E-04	0.43	1.54E-01	-1.02	1.80E-04	-1.45	1.56E-06
CFH	0.46	3.14E-01	0.61	3.41E-02	-0.44	1.99E-01	0.15	7.99E-01	-0.90	3.74E-03	-1.05	6.23E-04
CH25H	0.69	4.13E-01	0.30	6.77E-01	1.77	1.33E-03	-0.39	6.43E-01	1.08	4.26E-02	1.47	3.73E-03
CHAF1B	-0.43	5.39E-01	-0.61	1.13E-01	0.48	3.15E-01	-0.18	8.14E-01	0.91	2.33E-02	1.09	4.56E-03
CHI3L1	0.21	9.73E-01	-1.45	4.87E-01	2.53	1.99E-01	-1.67	5.28E-01	2.32	2.45E-01	3.99	1.85E-02
CHMP1B	-0.43	4.44E-01	-0.66	3.95E-02	0.60	1.01E-01	-0.23	6.85E-01	1.02	3.28E-03	1.26	2.96E-04
CHN1	0.21	9.01E-01	0.43	5.43E-01	-1.30	2.57E-02	0.21	8.56E-01	-1.52	8.67E-03	-1.73	1.90E-03
CHODL	-0.05	9.34E-01	0.05	8.87E-01	1.36	2.96E-07	0.10	7.86E-01	1.42	1.38E-07	1.31	2.52E-07
CHRDL1	0.52	5.97E-01	1.38	4.97E-03	3.33	5.50E-07	0.86	1.63E-01	2.82	4.83E-06	1.96	2.66E-04
CIT	-0.11	9.62E-01	-1.17	8.95E-02	0.82	3.48E-01	-1.06	2.23E-01	0.93	2.52E-01	1.99	4.09E-03
CKAP2	-0.30	7.31E-01	-0.66	1.01E-01	0.37	4.89E-01	-0.35	5.80E-01	0.68	1.22E-01	1.03	9.28E-03
CKAP2L	-0.86	6.10E-01	-1.49	8.53E-02	0.89	4.45E-01	-0.63	6.76E-01	1.75	5.79E-02	2.38	6.28E-03
CKS2	-0.61	6.34E-01	-1.24	4.94E-02	-0.05	9.73E-01	-0.63	5.30E-01	0.56	5.36E-01	1.19	7.03E-02
CLDN11	0.09	9.78E-01	-0.16	9.18E-01	1.99	2.72E-02	-0.25	8.98E-01	1.90	3.26E-02	2.15	1.08E-02
CLGN	-0.14	9.51E-01	-0.70	3.19E-01	3.47	6.42E-06	-0.56	5.81E-01	3.61	3.27E-06	4.17	2.39E-07
CLSPN	-0.93	5.74E-01	-1.26	1.64E-01	0.65	6.15E-01	-0.33	8.61E-01	1.58	9.42E-02	1.91	2.79E-02
CLU	0.55	8.09E-01	-0.07	9.69E-01	3.14	1.92E-03	-0.62	7.07E-01	2.59	8.34E-03	3.21	8.22E-04
CMAH	0.91	1.11E-01	0.73	7.33E-02	-0.24	7.21E-01	-0.18	8.31E-01	-1.15	7.72E-03	-0.97	1.77E-02
CMKLR1	1.99	1.08E-02	2.49	1.12E-04	-0.39	6.87E-01	0.50	6.21E-01	-2.38	3.77E-04	-2.88	2.59E-05

CNKSR2	-0.21	8.31E-01	-0.02	9.75E-01	2.70	1.94E-07	0.18	8.08E-01	2.91	4.36E-08	2.72	8.47E-08
CNN1	1.12	1.33E-01	0.81	1.34E-01	-0.96	9.97E-02	-0.31	7.47E-01	-2.08	3.31E-04	-1.77	1.10E-03
CNTN3	-0.10	9.54E-01	-0.04	9.68E-01	1.58	3.13E-03	0.06	9.62E-01	1.68	1.46E-03	1.62	1.33E-03
COBLL1	0.33	3.44E-01	1.22	2.68E-06	0.53	2.19E-02	0.89	5.90E-04	0.20	4.92E-01	-0.69	1.71E-03
COG6	0.16	7.44E-01	0.73	1.06E-03	-0.41	9.76E-02	0.56	2.52E-02	-0.57	1.51E-02	-1.13	1.07E-05
COL11A1	0.45	7.55E-01	0.65	3.72E-01	-1.08	1.27E-01	0.20	8.90E-01	-1.53	2.16E-02	-1.73	6.54E-03
COL14A1	0.91	7.57E-02	1.37	3.70E-04	0.94	1.90E-02	0.46	3.83E-01	0.03	9.72E-01	-0.43	3.36E-01
COL15A1	0.41	7.89E-01	1.31	3.69E-02	0.41	6.93E-01	0.90	2.88E-01	0.00	9.99E-01	-0.90	2.00E-01
COL1A1	0.43	5.28E-01	0.98	6.96E-03	-0.26	6.56E-01	0.55	2.62E-01	-0.69	8.91E-02	-1.24	1.25E-03
COL3A1	0.88	1.54E-02	1.74	1.14E-06	-0.23	5.74E-01	0.85	8.38E-03	-1.12	3.66E-04	-1.97	1.38E-07
COL4A1	-0.18	9.41E-01	0.18	8.88E-01	-2.11	7.12E-03	0.37	7.98E-01	-1.93	1.25E-02	-2.30	2.10E-03
COL4A2	-0.19	9.18E-01	0.01	9.93E-01	-1.46	1.48E-02	0.20	8.74E-01	-1.27	3.17E-02	-1.47	9.01E-03
COL5A2	0.51	1.77E-01	1.29	1.66E-05	-0.32	3.40E-01	0.78	9.30E-03	-0.83	3.06E-03	-1.61	1.06E-06
COL6A1	0.45	3.88E-01	1.32	9.34E-05	0.26	5.72E-01	0.87	1.52E-02	-0.19	7.10E-01	-1.06	1.17E-03
COLEC12	0.39	7.93E-01	0.13	9.05E-01	2.11	1.78E-03	-0.26	8.44E-01	1.72	8.50E-03	1.98	1.70E-03
COMP	0.52	7.65E-01	1.69	2.06E-02	0.48	6.93E-01	1.17	2.20E-01	-0.05	9.79E-01	-1.21	1.26E-01
COPZ2	0.27	5.40E-01	0.35	1.64E-01	-0.72	5.34E-03	0.07	8.93E-01	-0.99	1.99E-04	-1.06	5.39E-05
CPA4	-0.94	3.30E-01	-1.42	1.62E-02	-0.91	2.03E-01	-0.48	6.36E-01	0.03	9.83E-01	0.51	5.29E-01
CPE	0.67	5.93E-01	1.62	9.76E-03	-0.62	4.78E-01	0.95	2.58E-01	-1.29	6.12E-02	-2.24	8.11E-04
CPXM2	0.03	9.93E-01	0.11	9.26E-01	2.43	1.16E-03	0.09	9.64E-01	2.41	1.06E-03	2.32	9.50E-04
CRABP2	1.43	1.89E-01	2.37	1.39E-03	1.27	1.29E-01	0.95	3.59E-01	-0.15	9.23E-01	-1.10	1.75E-01
CRISPLD1	0.29	9.07E-01	0.83	3.62E-01	-0.95	3.45E-01	0.53	7.05E-01	-1.24	1.67E-01	-1.77	2.52E-02
CSGALNACT1	0.13	9.37E-01	-0.33	6.05E-01	0.68	2.34E-01	-0.46	5.46E-01	0.55	3.62E-01	1.01	3.63E-02
CSRP1	0.30	8.05E-01	0.23	7.59E-01	-1.11	3.44E-02	-0.07	9.60E-01	-1.41	6.53E-03	-1.34	6.60E-03
CSRP2	0.37	7.19E-01	1.38	3.08E-03	-0.87	9.68E-02	1.01	7.02E-02	-1.25	1.30E-02	-2.25	2.56E-05
CTSC	-0.10	9.32E-01	0.06	9.26E-01	3.28	1.52E-09	0.16	8.25E-01	3.38	8.08E-10	3.22	2.22E-09
CTSD	-0.47	5.54E-01	-0.58	1.91E-01	-1.51	1.14E-03	-0.11	9.12E-01	-1.05	1.89E-02	-0.93	2.75E-02
CXCL1	0.11	9.12E-01	0.50	1.14E-01	1.52	4.11E-05	0.39	3.64E-01	1.41	7.82E-05	1.01	1.58E-03
CXCL12	-0.12	9.31E-01	-0.17	7.97E-01	-1.09	1.27E-02	-0.05	9.65E-01	-0.97	2.44E-02	-0.92	2.49E-02
CXCL16	-0.47	4.90E-01	-0.24	6.56E-01	-1.26	2.84E-03	0.23	7.42E-01	-0.78	5.90E-02	-1.01	8.97E-03
CXCL2	-0.03	9.88E-01	-0.15	8.70E-01	1.99	6.38E-04	-0.12	9.29E-01	2.02	4.49E-04	2.14	1.55E-04
CYB5R1	-0.06	9.54E-01	-0.01	9.89E-01	-1.00	1.90E-03	0.05	9.44E-01	-0.94	2.75E-03	-1.00	1.08E-03
CYGB	-0.99	5.17E-02	-0.44	2.96E-01	-1.62	1.93E-04	0.54	2.74E-01	-0.63	1.36E-01	-1.18	2.34E-03
CYorf15A	-0.07	9.53E-01	-0.07	9.06E-01	1.31	4.02E-04	0.00	9.98E-01	1.39	1.80E-04	1.38	1.15E-04

CYP19A1	-0.12	9.33E-01	-0.16	8.15E-01	1.04	2.39E-02	-0.04	9.73E-01	1.16	1.08E-02	1.20	5.58E-03
DAAM1	0.73	1.30E-04	0.95	1.14E-06	-0.12	5.80E-01	0.21	3.08E-01	-0.86	5.06E-06	-1.07	1.38E-07
DACT1	-0.05	9.87E-01	0.15	8.98E-01	-1.75	1.42E-02	0.20	8.98E-01	-1.70	1.56E-02	-1.90	4.84E-03
DAPK1	0.31	7.86E-01	-0.32	6.30E-01	-1.73	1.16E-03	-0.63	3.39E-01	-2.05	1.65E-04	-1.42	3.88E-03
DCBLD1	-0.26	7.16E-01	0.44	2.02E-01	-0.82	1.89E-02	0.70	6.65E-02	-0.56	1.21E-01	-1.26	2.81E-04
DCUN1D3	0.52	9.79E-02	0.70	2.08E-03	-0.51	3.86E-02	0.18	6.55E-01	-1.03	7.52E-05	-1.21	6.81E-06
DDIT4	-0.68	2.95E-01	-1.12	5.67E-03	-0.52	3.22E-01	-0.44	4.83E-01	0.16	8.31E-01	0.60	1.84E-01
DDIT4L	0.14	8.63E-01	-0.19	6.24E-01	1.36	6.27E-05	-0.33	4.28E-01	1.22	1.76E-04	1.56	5.59E-06
DDX3Y	0.14	9.62E-01	0.12	9.39E-01	4.24	3.45E-05	-0.02	9.94E-01	4.10	4.14E-05	4.12	2.34E-05
DDX43	0.17	8.84E-01	0.15	8.07E-01	1.06	1.08E-02	-0.01	9.91E-01	0.89	3.05E-02	0.91	2.04E-02
DDX58	0.67	4.18E-01	1.99	1.40E-04	0.46	5.05E-01	1.32	1.88E-02	-0.22	8.07E-01	-1.53	2.44E-03
DDX60	0.26	8.29E-01	1.54	1.10E-03	0.29	7.13E-01	1.28	1.66E-02	0.02	9.83E-01	-1.26	8.37E-03
DDX60L	0.01	9.98E-01	1.14	1.31E-02	0.41	5.50E-01	1.13	3.53E-02	0.40	5.59E-01	-0.73	1.48E-01
DEPDC6	0.15	9.62E-01	0.04	9.84E-01	-3.35	7.16E-04	-0.11	9.65E-01	-3.49	3.80E-04	-3.38	3.44E-04
DGKH	-0.22	6.69E-01	-0.20	5.11E-01	1.05	1.95E-04	0.01	9.87E-01	1.26	1.36E-05	1.25	1.10E-05
DGKI	-0.11	9.34E-01	0.54	2.24E-01	0.89	4.95E-02	0.66	2.12E-01	1.00	2.33E-02	0.34	5.45E-01
DHFR	-0.75	3.86E-01	-1.22	1.72E-02	-0.11	9.28E-01	-0.47	5.83E-01	0.64	3.25E-01	1.11	3.61E-02
DHRS3	-0.79	1.96E-01	-1.70	1.21E-04	0.08	9.33E-01	-0.91	6.24E-02	0.87	5.07E-02	1.79	9.46E-05
DKK1	-1.22	3.81E-01	-1.73	3.87E-02	-1.06	3.25E-01	-0.51	7.45E-01	0.17	9.28E-01	0.68	5.66E-01
DLEU2	-0.07	9.56E-01	-0.30	4.97E-01	1.39	5.19E-04	-0.23	7.23E-01	1.46	2.30E-04	1.69	2.59E-05
DLGAP5	-1.80	1.56E-01	-2.99	8.20E-04	-0.02	9.94E-01	-1.19	3.23E-01	1.78	6.28E-02	2.97	1.18E-03
DLX3	-1.06	2.20E-01	-1.26	2.66E-02	-1.14	7.33E-02	-0.19	8.83E-01	-0.07	9.55E-01	0.12	9.18E-01
DLX5	-0.64	6.51E-01	-0.39	7.00E-01	-1.55	4.08E-02	0.25	8.75E-01	-0.91	2.83E-01	-1.15	1.17E-01
DMD	0.32	7.79E-01	0.11	8.98E-01	-1.03	4.91E-02	-0.21	8.32E-01	-1.35	8.40E-03	-1.14	1.89E-02
DNM1	-0.12	8.94E-01	-0.05	9.19E-01	0.89	4.32E-03	0.06	9.30E-01	1.00	1.21E-03	0.94	1.42E-03
DOCK11	-0.47	3.00E-01	-0.69	1.45E-02	0.78	1.23E-02	-0.22	6.56E-01	1.24	1.41E-04	1.47	1.17E-05
DOK6	0.26	6.37E-01	0.66	1.40E-02	-0.43	1.74E-01	0.39	2.74E-01	-0.69	1.71E-02	-1.09	2.17E-04
DSG2	-0.42	5.99E-01	-0.40	4.20E-01	1.96	5.36E-05	0.03	9.84E-01	2.38	3.29E-06	2.36	2.65E-06
DTX3L	0.22	8.36E-01	1.16	3.94E-03	0.40	4.79E-01	0.94	4.52E-02	0.18	8.08E-01	-0.76	7.05E-02
DUSP10	-0.63	2.83E-01	-1.14	2.29E-03	-2.50	5.50E-07	-0.51	3.14E-01	-1.88	2.22E-05	-1.36	5.87E-04
DYNC2H1	-0.53	3.54E-01	-1.04	2.69E-03	-0.43	3.36E-01	-0.51	2.64E-01	0.10	8.87E-01	0.61	9.43E-02
E2F8	-0.22	8.77E-01	-0.36	5.81E-01	0.78	1.65E-01	-0.14	9.08E-01	1.00	5.63E-02	1.14	2.08E-02
EBP	0.79	2.08E-01	0.53	2.43E-01	-0.35	5.71E-01	-0.25	7.45E-01	-1.13	1.12E-02	-0.88	3.89E-02
EDN1	-0.12	9.52E-01	-1.49	4.17E-03	-1.16	4.32E-02	-1.37	2.22E-02	-1.04	6.89E-02	0.33	6.83E-01

EDNRA	0.23	9.22E-01	0.38	7.12E-01	-1.53	4.46E-02	0.16	9.30E-01	-1.76	1.87E-02	-1.91	7.22E-03
EFHD1	0.03	9.89E-01	0.44	5.16E-01	-0.92	1.30E-01	0.41	6.58E-01	-0.95	1.09E-01	-1.36	1.17E-02
EFTUD1	-0.55	4.15E-01	-0.09	8.96E-01	0.48	3.46E-01	0.46	4.35E-01	1.04	1.51E-02	0.58	1.91E-01
EGR2	1.52	9.61E-02	1.37	3.50E-02	-0.92	2.46E-01	-0.15	9.27E-01	-2.44	7.05E-04	-2.28	8.59E-04
EIF1AY	0.02	9.97E-01	0.02	9.92E-01	4.49	2.58E-05	0.00	1.00E+00	4.47	1.98E-05	4.47	1.32E-05
ELK3	-0.19	8.11E-01	-0.07	9.05E-01	0.82	1.56E-02	0.12	8.62E-01	1.01	2.84E-03	0.89	5.58E-03
ELMO1	0.36	6.63E-01	0.06	9.32E-01	1.08	1.20E-02	-0.29	6.73E-01	0.72	1.01E-01	1.01	1.16E-02
ELTD1	0.00	9.99E-01	0.14	9.04E-01	-1.66	2.01E-02	0.15	9.30E-01	-1.66	1.88E-02	-1.81	7.26E-03
EMB	0.01	9.98E-01	0.36	2.84E-01	-1.26	2.36E-04	0.35	4.18E-01	-1.27	1.81E-04	-1.62	5.59E-06
EMP2	-0.40	2.92E-01	-1.00	9.68E-05	0.63	1.53E-02	-0.61	2.71E-02	1.03	1.67E-04	1.63	2.18E-07
ENO2	-1.30	2.22E-03	-1.69	1.32E-05	-1.02	5.41E-03	-0.39	4.17E-01	0.28	5.63E-01	0.68	4.84E-02
ENOX1	0.59	5.27E-01	1.05	3.28E-02	0.68	2.65E-01	0.47	5.67E-01	0.09	9.32E-01	-0.37	6.04E-01
ENPP4	-0.04	9.78E-01	0.20	6.74E-01	-0.86	1.81E-02	0.24	6.88E-01	-0.83	2.18E-02	-1.07	2.16E-03
ENTPD1	0.46	6.38E-01	0.42	4.78E-01	-0.56	3.72E-01	-0.04	9.81E-01	-1.01	4.90E-02	-0.98	4.52E-02
ENTPD7	0.23	7.95E-01	1.31	5.17E-04	0.10	8.94E-01	1.08	1.02E-02	-0.13	8.53E-01	-1.21	1.56E-03
EPB41L4A	0.23	8.20E-01	-0.01	9.86E-01	1.57	6.09E-04	-0.25	7.42E-01	1.34	2.42E-03	1.58	2.92E-04
EPGN	0.47	7.87E-01	2.32	1.39E-03	2.88	4.26E-04	1.84	2.52E-02	2.41	2.05E-03	0.56	5.81E-01
EPHA3	-0.07	9.81E-01	0.91	2.24E-01	-1.67	2.57E-02	0.97	2.93E-01	-1.60	2.98E-02	-2.58	4.47E-04
EPHB1	-0.33	5.74E-01	-0.28	4.38E-01	1.16	6.09E-04	0.04	9.60E-01	1.49	2.64E-05	1.44	2.48E-05
EPSTI1	0.20	8.64E-01	1.82	5.39E-05	0.28	6.62E-01	1.63	1.07E-03	0.08	9.23E-01	-1.54	4.10E-04
ERCC6	0.67	1.79E-01	1.51	5.91E-05	-1.03	5.98E-03	0.84	3.38E-02	-1.70	2.62E-05	-2.53	6.68E-08
ERG	-0.39	8.08E-01	-0.55	4.99E-01	1.66	1.77E-02	-0.16	9.19E-01	2.05	3.17E-03	2.21	1.04E-03
ERMN	0.56	5.42E-01	1.90	1.86E-04	-0.65	2.72E-01	1.35	1.39E-02	-1.21	1.92E-02	-2.55	7.17E-06
ERRFI1	-0.42	4.60E-01	-1.39	7.32E-05	-0.36	4.04E-01	-0.97	8.85E-03	0.06	9.32E-01	1.03	1.94E-03
ESCO2	-0.25	9.12E-01	-0.28	7.98E-01	1.18	1.31E-01	-0.04	9.86E-01	1.42	5.48E-02	1.46	3.61E-02
ESM1	-0.35	8.24E-01	-1.11	7.82E-02	1.42	3.51E-02	-0.76	4.00E-01	1.78	7.63E-03	2.53	1.77E-04
ETS1	-0.47	2.63E-01	-0.68	1.14E-02	0.42	1.94E-01	-0.20	6.68E-01	0.89	2.49E-03	1.09	2.09E-04
ETS2	-0.13	9.34E-01	-0.36	5.45E-01	1.74	6.35E-04	-0.23	7.99E-01	1.87	2.45E-04	2.10	3.94E-05
ETV1	-0.05	9.87E-01	-0.20	8.70E-01	1.75	2.20E-02	-0.16	9.31E-01	1.80	1.71E-02	1.96	6.64E-03
EXOSC8	-0.06	9.70E-01	0.26	6.52E-01	0.97	2.74E-02	0.31	6.56E-01	1.03	1.79E-02	0.72	9.40E-02
EYA1	0.16	9.25E-01	0.01	9.95E-01	-1.31	1.74E-02	-0.15	8.98E-01	-1.47	6.99E-03	-1.32	1.07E-02
EZH2	-0.62	3.45E-01	-0.51	2.60E-01	0.69	1.37E-01	0.12	9.03E-01	1.32	2.74E-03	1.20	4.09E-03
F2RL2	-0.94	2.43E-01	-0.89	9.74E-02	0.95	1.06E-01	0.05	9.77E-01	1.89	1.02E-03	1.84	8.19E-04
FADS1	1.11	1.11E-01	1.28	8.09E-03	0.86	1.23E-01	0.17	8.81E-01	-0.25	7.68E-01	-0.42	5.33E-01

FADS2	1.71	2.38E-02	1.75	2.40E-03	0.83	2.37E-01	0.05	9.79E-01	-0.88	1.91E-01	-0.93	1.36E-01
FAM111B	-1.26	3.61E-01	-1.53	7.28E-02	0.61	6.42E-01	-0.27	8.93E-01	1.87	3.89E-02	2.14	1.25E-02
FAM129A	-0.38	8.17E-01	0.03	9.84E-01	1.71	1.41E-02	0.41	7.34E-01	2.09	2.64E-03	1.68	1.01E-02
FAM151B	0.38	4.52E-01	0.91	1.80E-03	-0.69	2.79E-02	0.53	1.39E-01	-1.07	7.66E-04	-1.60	4.64E-06
FAM180A	-1.15	3.40E-03	-0.81	7.43E-03	0.59	8.39E-02	0.34	4.65E-01	1.74	4.83E-06	1.40	4.90E-05
FAM198B	0.49	5.58E-01	0.73	1.07E-01	-0.77	1.23E-01	0.24	7.80E-01	-1.26	7.54E-03	-1.50	1.08E-03
FAM20C	-0.18	6.41E-01	-0.23	2.81E-01	0.85	1.29E-04	-0.05	9.17E-01	1.04	8.62E-06	1.08	3.50E-06
FAM26E	0.35	6.10E-01	0.59	9.20E-02	-1.23	1.28E-03	0.24	6.94E-01	-1.58	6.99E-05	-1.82	7.17E-06
FAM49A	-0.07	9.75E-01	-0.14	8.96E-01	1.76	5.23E-03	-0.07	9.69E-01	1.83	3.19E-03	1.89	1.52E-03
FAM54A	-0.84	5.11E-01	-1.07	1.42E-01	1.00	2.39E-01	-0.23	8.84E-01	1.84	1.42E-02	2.07	3.92E-03
FAM69A	0.25	6.38E-01	0.25	4.28E-01	-1.06	3.59E-04	0.00	1.00E+00	-1.31	2.16E-05	-1.31	1.45E-05
FAM72D	-0.48	6.45E-01	-0.76	1.60E-01	0.60	3.68E-01	-0.28	7.81E-01	1.08	5.07E-02	1.36	9.15E-03
FAM72D	-0.50	6.46E-01	-0.79	1.67E-01	0.62	3.79E-01	-0.28	7.88E-01	1.12	5.41E-02	1.41	1.01E-02
FAM72D	-0.55	5.99E-01	-0.79	1.62E-01	0.63	3.66E-01	-0.23	8.37E-01	1.18	3.92E-02	1.41	9.21E-03
FAM72D	-0.63	6.28E-01	-0.81	2.52E-01	0.87	2.72E-01	-0.18	9.09E-01	1.50	2.94E-02	1.68	1.02E-02
FANCD2	-0.26	8.48E-01	-0.27	7.23E-01	0.74	2.14E-01	-0.01	9.96E-01	1.00	6.70E-02	1.01	4.97E-02
FANK1	-0.04	9.67E-01	-0.12	7.96E-01	-1.47	1.69E-05	-0.07	9.13E-01	-1.43	1.53E-05	-1.35	2.04E-05
FBLN1	-0.23	8.85E-01	-0.58	3.50E-01	0.98	1.08E-01	-0.35	7.26E-01	1.21	3.67E-02	1.56	4.84E-03
FBLN2	-0.01	9.95E-01	-0.13	8.18E-01	0.88	1.46E-02	-0.11	8.79E-01	0.89	1.21E-02	1.01	3.24E-03
FBLN5	0.45	4.39E-01	0.74	2.89E-02	-0.39	3.88E-01	0.28	6.19E-01	-0.84	2.08E-02	-1.12	1.46E-03
FBXL5	1.00	2.05E-05	0.92	9.65E-06	-0.18	4.68E-01	-0.08	8.32E-01	-1.18	5.42E-07	-1.10	1.06E-06
FBXO16	0.25	8.19E-01	0.74	8.62E-02	-1.13	1.43E-02	0.50	4.25E-01	-1.37	2.72E-03	-1.87	7.34E-05
FBXO5	-0.39	7.37E-01	-0.74	1.79E-01	1.13	4.43E-02	-0.34	7.14E-01	1.52	5.81E-03	1.87	6.12E-04
FDPS	1.24	3.76E-02	1.02	2.11E-02	0.06	9.54E-01	-0.22	8.08E-01	-1.17	1.42E-02	-0.96	3.66E-02
FERMT1	-0.02	9.88E-01	0.17	6.11E-01	1.01	2.28E-04	0.18	6.63E-01	1.03	1.57E-04	0.85	7.55E-04
FGD6	-0.17	9.23E-01	-0.40	5.65E-01	1.38	1.64E-02	-0.23	8.37E-01	1.55	6.34E-03	1.78	1.25E-03
FGF7	0.07	9.38E-01	-0.21	5.39E-01	-1.13	2.40E-04	-0.28	4.92E-01	-1.20	9.56E-05	-0.91	1.06E-03
FGL2	-0.49	9.01E-01	2.42	5.03E-02	3.96	3.59E-03	2.91	4.20E-02	4.45	1.03E-03	1.54	2.89E-01
FIBIN	-0.03	9.87E-01	-0.39	4.49E-01	0.72	1.38E-01	-0.36	6.07E-01	0.75	1.14E-01	1.11	9.84E-03
FIGN	0.13	8.87E-01	0.14	7.63E-01	-1.43	7.59E-05	0.01	9.91E-01	-1.56	1.81E-05	-1.57	1.12E-05
FILIP1	0.17	8.18E-01	1.22	1.02E-04	-0.10	8.66E-01	1.05	2.38E-03	-0.27	5.11E-01	-1.32	5.34E-05
FKBP14	0.51	5.22E-02	1.07	4.48E-06	-0.15	6.23E-01	0.55	1.30E-02	-0.66	1.89E-03	-1.22	1.06E-06
FLG	0.73	7.29E-01	1.70	7.49E-02	-1.58	1.46E-01	0.97	5.11E-01	-2.31	2.19E-02	-3.28	8.99E-04
FLJ39632	-0.35	5.94E-01	0.05	9.30E-01	-1.17	1.57E-03	0.40	4.02E-01	-0.82	2.09E-02	-1.22	5.30E-04

FLJ39632	-0.33	6.19E-01	0.08	8.98E-01	-1.08	2.98E-03	0.40	3.92E-01	-0.75	3.36E-02	-1.15	8.34E-04
FLNB	-0.37	6.38E-01	-1.27	1.06E-03	-1.04	1.24E-02	-0.90	4.20E-02	-0.67	1.19E-01	0.23	6.98E-01
FLRT2	-0.06	9.80E-01	0.18	8.67E-01	1.25	5.47E-02	0.23	8.56E-01	1.31	3.96E-02	1.08	8.15E-02
FMN1	-0.08	9.67E-01	0.36	6.07E-01	1.57	4.78E-03	0.43	6.10E-01	1.65	2.68E-03	1.22	1.86E-02
FMNL2	-0.16	8.00E-01	-0.02	9.74E-01	1.30	2.22E-05	0.14	7.72E-01	1.46	3.65E-06	1.32	8.46E-06
FNIP2	-0.30	8.47E-01	0.39	6.23E-01	-1.20	5.75E-02	0.69	4.07E-01	-0.90	1.74E-01	-1.58	6.78E-03
FOSL1	-0.15	7.72E-01	-0.39	7.96E-02	0.61	1.06E-02	-0.24	4.72E-01	0.77	1.51E-03	1.01	5.04E-05
FOXM1	-0.80	3.50E-01	-1.10	3.52E-02	0.81	1.95E-01	-0.30	7.68E-01	1.61	4.43E-03	1.91	6.27E-04
FOXN3	-0.06	9.57E-01	-0.53	1.08E-01	0.89	9.41E-03	-0.46	2.72E-01	0.96	4.91E-03	1.42	5.96E-05
FRMD4A	0.36	4.15E-01	0.41	1.32E-01	-0.88	2.46E-03	0.05	9.45E-01	-1.24	5.22E-05	-1.29	1.92E-05
FRMD4B	0.06	9.77E-01	-0.11	9.18E-01	2.00	1.09E-03	-0.17	8.98E-01	1.94	1.21E-03	2.11	3.35E-04
FRMPD3	-0.03	9.85E-01	-0.12	8.61E-01	0.94	2.77E-02	-0.09	9.28E-01	0.97	2.09E-02	1.06	8.15E-03
FRRS1	-0.06	9.63E-01	-0.51	2.27E-01	-1.29	2.66E-03	-0.45	4.34E-01	-1.23	3.33E-03	-0.78	5.06E-02
FRZB	0.22	8.70E-01	-0.43	4.51E-01	-0.82	1.21E-01	-0.65	3.04E-01	-1.04	3.81E-02	-0.39	5.43E-01
GABBR2	0.12	9.18E-01	-0.04	9.57E-01	1.34	6.09E-04	-0.15	8.34E-01	1.22	1.24E-03	1.38	2.41E-04
GALNT13	-0.02	9.93E-01	0.04	9.62E-01	1.80	5.70E-05	0.05	9.60E-01	1.82	3.92E-05	1.76	3.35E-05
GAP43	1.07	1.07E-01	1.23	7.89E-03	-0.14	8.88E-01	0.16	8.84E-01	-1.21	1.62E-02	-1.37	4.60E-03
GCH1	0.15	9.54E-01	0.68	4.04E-01	1.51	4.39E-02	0.54	6.51E-01	1.37	6.88E-02	0.83	3.11E-01
GCLC	-0.06	9.57E-01	0.16	7.30E-01	1.24	3.94E-04	0.22	6.85E-01	1.30	1.86E-04	1.08	8.29E-04
GCLM	0.83	8.17E-02	1.38	1.49E-04	1.18	1.92E-03	0.55	2.10E-01	0.34	4.59E-01	-0.20	7.03E-01
GCNT4	0.53	5.00E-01	-0.45	3.68E-01	-1.65	6.46E-04	-0.98	5.06E-02	-2.17	2.07E-05	-1.19	6.58E-03
GCOM1	0.03	9.87E-01	-0.04	9.63E-01	1.17	5.48E-03	-0.06	9.54E-01	1.14	5.74E-03	1.20	2.46E-03
GDF15	-0.52	4.17E-01	-1.03	5.25E-03	-0.48	3.15E-01	-0.51	3.15E-01	0.04	9.60E-01	0.55	1.79E-01
GFPT2	0.07	9.78E-01	-0.33	7.51E-01	1.64	2.74E-02	-0.41	7.51E-01	1.56	3.27E-02	1.97	4.86E-03
GFRA1	0.37	7.74E-01	0.65	2.78E-01	2.29	2.28E-04	0.28	7.90E-01	1.92	1.14E-03	1.64	3.16E-03
GK	0.12	9.30E-01	0.12	8.70E-01	1.03	2.58E-02	0.00	9.99E-01	0.90	4.89E-02	0.90	3.68E-02
GLIPR2	0.73	1.61E-02	0.70	2.50E-03	-0.60	1.78E-02	-0.03	9.66E-01	-1.33	4.25E-06	-1.30	4.01E-06
GLIS3	-0.04	9.84E-01	0.23	7.67E-01	1.33	1.28E-02	0.27	7.77E-01	1.37	9.61E-03	1.10	2.83E-02
GLRB	0.53	3.04E-01	1.04	1.66E-03	0.24	6.38E-01	0.51	2.33E-01	-0.29	5.35E-01	-0.80	1.68E-02
GLT8D2	0.59	4.09E-01	0.62	1.64E-01	1.07	1.89E-02	0.03	9.82E-01	0.48	3.73E-01	0.45	3.87E-01
GLUL	-0.09	8.96E-01	-0.54	1.60E-02	0.62	1.23E-02	-0.45	1.04E-01	0.71	3.63E-03	1.16	1.30E-05
GLUL	-0.21	7.86E-01	-0.82	1.01E-02	0.97	5.98E-03	-0.61	1.24E-01	1.18	8.84E-04	1.79	4.64E-06
GLYATL2	-0.12	8.95E-01	-0.37	2.52E-01	0.85	7.47E-03	-0.25	6.10E-01	0.97	2.17E-03	1.22	1.39E-04
GMFG	-0.90	1.54E-01	-1.65	2.99E-04	-2.07	6.27E-05	-0.75	1.70E-01	-1.17	1.21E-02	-0.42	4.52E-01

GNPDA1	-0.76	1.69E-01	-1.04	5.71E-03	-0.13	8.62E-01	-0.28	6.85E-01	0.63	1.48E-01	0.90	1.98E-02
GPR126	-0.10	9.59E-01	0.04	9.69E-01	2.10	4.41E-04	0.14	9.13E-01	2.20	2.12E-04	2.06	2.79E-04
GPR137C	-0.15	9.09E-01	-0.12	8.67E-01	0.99	2.37E-02	0.03	9.82E-01	1.14	8.66E-03	1.11	7.10E-03
GPR155	0.22	8.55E-01	1.07	1.35E-02	-0.23	7.67E-01	0.85	1.12E-01	-0.45	4.50E-01	-1.30	4.03E-03
GPRC5A	-0.28	8.33E-01	-1.84	4.69E-04	0.55	4.24E-01	-1.56	8.28E-03	0.83	1.55E-01	2.38	3.47E-05
GPX7	0.48	3.88E-01	0.74	2.30E-02	-0.42	3.18E-01	0.27	6.35E-01	-0.89	1.17E-02	-1.16	8.45E-04
GRB14	0.50	5.00E-01	1.38	7.73E-04	0.56	2.56E-01	0.89	6.22E-02	0.07	9.41E-01	-0.82	4.69E-02
GREM2	-0.57	4.16E-01	-0.72	8.68E-02	1.01	2.40E-02	-0.15	8.70E-01	1.57	5.37E-04	1.72	1.31E-04
GRIA3	-0.02	9.87E-01	0.51	8.52E-02	-0.69	2.72E-02	0.53	1.37E-01	-0.67	2.96E-02	-1.20	1.44E-04
GRIK2	0.01	9.98E-01	0.38	7.31E-01	-1.52	5.51E-02	0.36	8.01E-01	-1.53	4.91E-02	-1.90	9.76E-03
GSTM3	0.52	2.13E-01	0.27	4.09E-01	-1.11	4.06E-04	-0.24	5.91E-01	-1.62	3.22E-06	-1.38	1.52E-05
GSTP1	-0.11	9.14E-01	-0.12	8.14E-01	-1.01	4.00E-03	-0.01	9.91E-01	-0.90	9.05E-03	-0.88	6.78E-03
GUCY1B3	1.18	2.13E-01	1.90	2.43E-03	-0.82	2.95E-01	0.72	4.33E-01	-2.00	3.14E-03	-2.72	8.89E-05
GXYLT2	0.77	1.97E-01	0.87	2.82E-02	-0.76	8.82E-02	0.10	9.17E-01	-1.52	5.58E-04	-1.63	1.82E-04
GYPC	-0.12	9.22E-01	-0.76	4.27E-02	0.58	1.92E-01	-0.63	1.77E-01	0.70	9.18E-02	1.33	7.48E-04
HELLS	-0.91	3.85E-01	-0.79	2.51E-01	0.78	3.28E-01	0.11	9.45E-01	1.69	1.20E-02	1.57	1.36E-02
HERC6	0.15	9.49E-01	3.02	3.10E-05	0.09	9.55E-01	2.87	3.81E-04	-0.06	9.65E-01	-2.93	5.22E-05
HEY2	0.53	5.85E-01	0.78	1.38E-01	-0.38	6.23E-01	0.24	8.11E-01	-0.91	1.03E-01	-1.15	2.36E-02
HGF	0.60	6.94E-01	0.22	8.57E-01	-1.59	3.89E-02	-0.37	7.87E-01	-2.18	3.87E-03	-1.81	1.14E-02
HHIP	-0.40	7.78E-01	-0.43	5.94E-01	1.23	6.29E-02	-0.03	9.89E-01	1.63	1.10E-02	1.66	6.74E-03
HIST1H1A	-0.57	7.04E-01	-0.94	2.08E-01	0.48	6.67E-01	-0.37	7.88E-01	1.05	1.96E-01	1.42	4.67E-02
HIST1H1D	-0.49	6.23E-01	-0.83	1.03E-01	1.20	2.67E-02	-0.34	7.04E-01	1.69	1.79E-03	2.03	1.73E-04
HIST1H1E	-0.68	2.18E-01	-0.96	7.56E-03	0.33	5.19E-01	-0.28	6.60E-01	1.01	9.76E-03	1.29	7.70E-04
HIST1H2BH	-0.36	7.88E-01	-0.60	3.41E-01	0.80	2.31E-01	-0.24	8.34E-01	1.16	5.32E-02	1.40	1.25E-02
HIST1H3B	-0.46	8.06E-01	-0.63	5.17E-01	1.17	1.86E-01	-0.16	9.32E-01	1.64	4.48E-02	1.80	1.90E-02
HIST1H3I	-1.11	1.27E-01	-1.87	3.79E-04	0.42	5.70E-01	-0.76	2.52E-01	1.53	4.81E-03	2.29	5.27E-05
HIST1H4C	-0.62	4.99E-01	-1.16	1.98E-02	0.27	7.55E-01	-0.54	4.89E-01	0.89	1.14E-01	1.43	5.58E-03
HIST2H2AB	-0.15	8.83E-01	-0.51	1.49E-01	0.56	1.54E-01	-0.36	4.87E-01	0.71	5.34E-02	1.07	2.20E-03
HJURP	-0.88	3.57E-01	-1.14	5.06E-02	0.31	7.62E-01	-0.26	8.32E-01	1.19	6.06E-02	1.44	1.46E-02
HLA-DMA	0.13	8.46E-01	-0.39	1.40E-01	-0.93	9.22E-04	-0.52	8.22E-02	-1.06	1.86E-04	-0.54	3.40E-02
HLA-DMA	0.13	8.46E-01	-0.39	1.40E-01	-0.93	9.22E-04	-0.52	8.22E-02	-1.06	1.86E-04	-0.54	3.40E-02
HLA-DPB1	-0.49	5.70E-01	-0.65	1.73E-01	-1.02	3.66E-02	-0.16	8.77E-01	-0.53	3.54E-01	-0.37	5.47E-01
HMGB2	-0.57	4.30E-01	-1.60	2.57E-04	0.45	4.20E-01	-1.04	3.12E-02	1.02	2.28E-02	2.06	1.92E-05
HMGCR	1.27	9.04E-02	1.41	7.44E-03	0.38	6.51E-01	0.14	9.17E-01	-0.89	1.43E-01	-1.03	6.32E-02

HMGCS1	1.67	5.38E-02	1.96	2.24E-03	0.63	4.75E-01	0.29	8.26E-01	-1.04	1.56E-01	-1.33	4.22E-02
HMMR	-1.54	2.26E-01	-2.22	7.41E-03	0.45	7.55E-01	-0.67	6.40E-01	2.00	2.64E-02	2.67	2.14E-03
HMOX1	-1.73	1.89E-02	-2.21	2.12E-04	0.57	4.61E-01	-0.48	6.10E-01	2.30	2.73E-04	2.78	1.84E-05
HNMT	0.03	9.85E-01	-0.44	1.87E-01	-1.34	1.97E-04	-0.46	2.57E-01	-1.37	1.24E-04	-0.90	4.68E-03
HSD17B6	1.08	1.77E-01	0.86	1.32E-01	-2.35	2.00E-04	-0.22	8.54E-01	-3.43	1.30E-06	-3.21	2.27E-06
HSPC159	0.66	2.02E-01	1.04	2.59E-03	0.41	3.63E-01	0.38	4.65E-01	-0.25	6.40E-01	-0.63	8.05E-02
HTR2A	0.43	8.43E-01	2.58	1.73E-03	-1.31	1.69E-01	2.14	2.21E-02	-1.75	4.85E-02	-3.89	2.91E-05
ICAM1	-0.16	9.21E-01	-0.25	7.27E-01	0.82	1.32E-01	-0.10	9.37E-01	0.98	5.96E-02	1.07	2.75E-02
ID2	-1.05	5.06E-04	-1.51	1.14E-06	0.41	1.40E-01	-0.47	1.08E-01	1.45	2.20E-06	1.92	2.41E-08
ID3	-1.57	9.01E-05	-1.38	5.62E-05	0.07	9.21E-01	0.19	7.42E-01	1.64	1.22E-05	1.45	3.74E-05
IDI1	0.94	7.07E-02	1.02	6.15E-03	0.43	3.90E-01	0.07	9.39E-01	-0.52	2.56E-01	-0.59	1.50E-01
IER3	1.05	1.67E-02	1.04	1.96E-03	0.05	9.51E-01	-0.01	9.96E-01	-1.00	5.56E-03	-0.99	3.92E-03
IFI27	-0.32	7.93E-01	1.30	8.84E-03	-0.96	8.59E-02	1.62	5.58E-03	-0.64	3.06E-01	-2.26	5.79E-05
IFI30	-0.79	4.31E-01	-1.20	3.83E-02	-1.88	3.37E-03	-0.41	6.94E-01	-1.09	8.95E-02	-0.68	3.32E-01
IFI44	0.32	7.87E-01	2.45	1.66E-05	0.65	2.95E-01	2.13	5.79E-04	0.32	6.82E-01	-1.80	6.09E-04
IFI44L	0.24	9.25E-01	2.54	1.23E-03	0.58	6.30E-01	2.29	9.53E-03	0.34	8.11E-01	-1.96	1.30E-02
IFI6	0.49	3.37E-01	2.21	3.11E-07	-0.24	6.28E-01	1.72	3.08E-05	-0.73	3.24E-02	-2.44	3.89E-08
IFIH1	0.10	9.61E-01	1.65	4.65E-03	-0.02	9.89E-01	1.54	2.19E-02	-0.12	9.22E-01	-1.67	5.55E-03
IFIT1	-0.04	9.93E-01	2.58	7.82E-03	0.28	8.96E-01	2.62	2.00E-02	0.32	8.67E-01	-2.30	2.16E-02
IFIT3	0.20	9.41E-01	1.94	1.03E-02	0.51	6.82E-01	1.74	5.20E-02	0.31	8.25E-01	-1.43	7.31E-02
IFITM1	-0.50	7.03E-01	1.63	6.40E-03	-0.93	1.95E-01	2.14	2.92E-03	-0.43	6.51E-01	-2.56	1.23E-04
IGFBP6	-0.17	7.36E-01	-1.08	2.31E-05	0.21	5.02E-01	-0.91	8.99E-04	0.38	1.30E-01	1.29	2.89E-06
IGJ	-0.09	9.64E-01	-0.54	4.03E-01	1.22	3.87E-02	-0.45	6.14E-01	1.30	2.41E-02	1.76	1.69E-03
IGSF10	-0.07	9.73E-01	-0.10	9.23E-01	1.23	3.09E-02	-0.03	9.87E-01	1.29	2.08E-02	1.32	1.27E-02
IL1R1	-0.19	9.15E-01	-0.51	4.39E-01	1.04	8.28E-02	-0.32	7.51E-01	1.22	3.40E-02	1.54	5.07E-03
IL1RAP	0.61	3.00E-01	1.39	3.54E-04	0.57	2.01E-01	0.78	7.94E-02	-0.05	9.56E-01	-0.82	3.03E-02
IL7R	-2.08	8.20E-02	-2.74	1.51E-03	-2.08	2.62E-02	-0.66	6.58E-01	0.00	9.99E-01	0.67	5.87E-01
IL8	0.05	9.78E-01	0.30	6.66E-01	1.14	2.90E-02	0.25	7.97E-01	1.09	3.47E-02	0.85	9.53E-02
INA	0.83	1.55E-01	0.88	2.88E-02	-0.78	8.25E-02	0.04	9.71E-01	-1.62	3.54E-04	-1.66	1.69E-04
INSIG2	-0.10	9.01E-01	0.32	2.13E-01	-1.72	2.29E-07	0.42	1.61E-01	-1.62	4.41E-07	-2.05	7.82E-09
ITGA10	-0.26	8.12E-01	-0.48	3.37E-01	0.73	1.48E-01	-0.22	7.99E-01	0.99	3.48E-02	1.22	6.68E-03
ITGA2	0.31	7.67E-01	0.70	1.28E-01	2.02	9.65E-05	0.39	5.92E-01	1.71	4.76E-04	1.32	3.56E-03
ITGA4	0.49	4.29E-01	0.46	2.46E-01	-2.09	5.17E-06	-0.03	9.78E-01	-2.58	1.87E-07	-2.55	1.38E-07
ITGA6	0.17	8.67E-01	-0.25	5.93E-01	1.27	1.25E-03	-0.42	4.06E-01	1.10	3.63E-03	1.52	9.24E-05

ITGA8	-1.86	5.42E-03	-2.99	2.63E-06	-3.06	3.72E-06	-1.14	6.34E-02	-1.20	3.13E-02	-0.06	9.57E-01
ITIH5	0.70	4.34E-01	1.33	9.73E-03	-0.68	2.97E-01	0.63	4.07E-01	-1.39	1.33E-02	-2.02	3.32E-04
JAG1	0.46	6.41E-01	0.80	1.14E-01	-1.54	4.12E-03	0.34	7.05E-01	-2.00	2.67E-04	-2.34	2.68E-05
JAM2	0.54	6.45E-01	0.26	7.85E-01	-0.91	1.84E-01	-0.29	8.07E-01	-1.46	1.98E-02	-1.17	5.17E-02
KAL1	0.71	3.75E-01	2.17	5.39E-05	-2.28	7.12E-05	1.46	9.30E-03	-2.99	1.62E-06	-4.45	3.04E-09
KCNA1	-0.06	9.63E-01	-0.11	8.59E-01	1.27	1.02E-03	-0.05	9.59E-01	1.32	5.26E-04	1.37	2.32E-04
KCND2	-0.50	5.61E-01	-0.64	1.82E-01	-1.17	1.74E-02	-0.14	8.95E-01	-0.66	2.13E-01	-0.52	3.38E-01
KCNE4	-0.01	9.98E-01	0.44	3.30E-01	-1.18	5.90E-03	0.45	4.41E-01	-1.17	5.41E-03	-1.62	1.57E-04
KCNH1	-0.91	2.40E-01	-0.94	6.66E-02	-2.22	1.61E-04	-0.03	9.87E-01	-1.31	1.59E-02	-1.28	1.28E-02
KCNJ2	-0.34	6.45E-01	-0.57	1.34E-01	1.20	2.62E-03	-0.23	7.35E-01	1.54	1.75E-04	1.77	2.04E-05
KCNMB1	0.63	3.91E-01	0.38	5.00E-01	-1.12	1.94E-02	-0.25	7.62E-01	-1.76	3.80E-04	-1.50	1.16E-03
KGFLP1	0.17	8.53E-01	-0.13	8.14E-01	-1.03	4.80E-03	-0.30	5.90E-01	-1.20	1.04E-03	-0.90	8.10E-03
KGFLP1	0.17	8.53E-01	-0.13	8.14E-01	-1.03	4.80E-03	-0.30	5.90E-01	-1.20	1.04E-03	-0.90	8.10E-03
KGFLP1	0.17	8.53E-01	-0.13	8.14E-01	-1.03	4.80E-03	-0.30	5.90E-01	-1.20	1.04E-03	-0.90	8.10E-03
KGFLP1	0.19	7.60E-01	-0.08	8.60E-01	-0.91	1.92E-03	-0.27	5.15E-01	-1.10	2.29E-04	-0.83	2.46E-03
KHDRBS3	0.24	8.43E-01	0.10	9.04E-01	-1.13	2.14E-02	-0.14	8.95E-01	-1.36	4.81E-03	-1.22	7.76E-03
KIAA0101	-1.48	1.77E-01	-2.04	5.88E-03	-0.10	9.54E-01	-0.56	6.74E-01	1.38	9.86E-02	1.94	1.13E-02
KIAA1524	-0.35	7.25E-01	-0.45	3.96E-01	1.07	2.84E-02	-0.10	9.30E-01	1.42	3.28E-03	1.52	1.15E-03
KIF11	-0.93	4.24E-01	-1.24	6.89E-02	0.72	4.32E-01	-0.32	8.19E-01	1.64	2.34E-02	1.96	4.73E-03
KIF14	-0.70	4.91E-01	-0.89	1.32E-01	1.04	1.03E-01	-0.18	8.89E-01	1.74	4.12E-03	1.92	1.11E-03
KIF15	-0.48	7.58E-01	-0.61	4.70E-01	0.87	3.03E-01	-0.12	9.45E-01	1.36	6.55E-02	1.48	3.18E-02
KIF18A	-0.93	4.34E-01	-1.12	1.20E-01	0.48	6.61E-01	-0.18	9.15E-01	1.41	6.02E-02	1.60	2.34E-02
KIF20A	-1.44	2.41E-01	-2.55	1.60E-03	0.09	9.65E-01	-1.11	3.09E-01	1.52	8.52E-02	2.63	1.57E-03
KIF20B	-0.52	6.89E-01	-0.82	2.12E-01	0.98	1.69E-01	-0.30	8.09E-01	1.50	2.15E-02	1.80	4.03E-03
KIF4A	-1.27	1.55E-01	-1.53	1.17E-02	0.23	8.52E-01	-0.26	8.44E-01	1.50	2.35E-02	1.76	5.40E-03
KITLG	-0.30	8.30E-01	0.20	8.27E-01	1.18	4.61E-02	0.50	5.75E-01	1.48	1.07E-02	0.98	8.15E-02
KLF12	-0.20	7.16E-01	-0.38	1.34E-01	-1.17	5.94E-05	-0.19	6.63E-01	-0.97	3.77E-04	-0.79	1.95E-03
KLHL13	-0.81	2.12E-02	-1.09	1.44E-04	-1.31	4.11E-05	-0.28	5.07E-01	-0.51	8.66E-02	-0.23	5.29E-01
KLHL24	-0.58	4.55E-01	-1.09	1.26E-02	-1.01	3.84E-02	-0.51	4.38E-01	-0.42	4.96E-01	0.09	9.27E-01
KNTC1	-0.27	8.18E-01	-0.29	6.59E-01	0.79	1.42E-01	-0.02	9.88E-01	1.06	3.44E-02	1.09	2.28E-02
KRT14	0.90	4.67E-01	2.87	1.31E-04	0.47	6.74E-01	1.97	1.39E-02	-0.43	7.09E-01	-2.40	1.08E-03
KRT16	1.20	2.75E-01	2.52	4.75E-04	-0.29	8.24E-01	1.32	1.20E-01	-1.49	4.85E-02	-2.81	2.09E-04
KRT16P3	0.55	3.32E-01	1.29	4.23E-04	0.16	8.02E-01	0.74	7.96E-02	-0.39	4.06E-01	-1.12	2.15E-03
KRT17	0.30	6.86E-01	1.06	2.43E-03	-0.04	9.62E-01	0.76	6.68E-02	-0.34	4.79E-01	-1.10	2.33E-03

KRT18	-0.08	9.64E-01	0.75	1.43E-01	-0.56	3.88E-01	0.83	1.77E-01	-0.48	4.75E-01	-1.31	8.72E-03
KRTAP1-1	-0.96	1.73E-01	-1.42	3.11E-03	-1.65	2.03E-03	-0.46	5.58E-01	-0.69	2.31E-01	-0.23	7.76E-01
KRTAP1-1	-0.65	1.56E-01	-0.73	1.95E-02	-1.11	1.58E-03	-0.08	9.19E-01	-0.46	2.16E-01	-0.38	3.10E-01
KRTAP1-1	-0.65	1.56E-01	-0.73	1.95E-02	-1.11	1.58E-03	-0.08	9.19E-01	-0.46	2.16E-01	-0.38	3.10E-01
KRTAP1-5	-1.05	3.12E-01	-1.58	1.50E-02	-2.02	5.01E-03	-0.52	6.41E-01	-0.97	2.10E-01	-0.45	6.51E-01
KRTAP1-5	-1.05	3.12E-01	-1.58	1.50E-02	-2.02	5.01E-03	-0.52	6.41E-01	-0.97	2.10E-01	-0.45	6.51E-01
LAMA1	-0.56	4.31E-01	-0.32	5.54E-01	1.19	8.21E-03	0.24	7.60E-01	1.74	1.86E-04	1.51	5.78E-04
LAMA2	0.57	5.81E-01	0.88	1.11E-01	1.50	9.44E-03	0.31	7.62E-01	0.93	1.20E-01	0.62	3.32E-01
LAMC2	0.02	9.95E-01	-0.03	9.83E-01	2.87	2.63E-04	-0.05	9.81E-01	2.85	2.29E-04	2.90	1.19E-04
LAPTM5	-0.81	5.78E-01	-1.56	3.87E-02	0.18	9.13E-01	-0.74	5.39E-01	1.00	2.81E-01	1.74	2.37E-02
LBH	-0.75	1.52E-01	-0.65	7.63E-02	-1.08	6.33E-03	0.10	9.09E-01	-0.33	5.28E-01	-0.43	3.29E-01
LEPR	0.03	9.85E-01	-0.40	2.70E-01	-1.53	6.61E-05	-0.42	3.49E-01	-1.56	4.29E-05	-1.13	8.60E-04
LEPREL1	-0.39	7.33E-01	-0.99	4.81E-02	1.18	3.00E-02	-0.60	4.05E-01	1.57	3.57E-03	2.17	8.90E-05
LGALS3BP	0.00	9.98E-01	0.73	5.78E-03	-1.40	2.22E-05	0.74	1.67E-02	-1.40	1.53E-05	-2.14	2.73E-08
LGI2	0.07	9.68E-01	-0.03	9.72E-01	1.68	1.33E-03	-0.10	9.32E-01	1.61	1.63E-03	1.72	5.78E-04
LGR4	0.94	9.54E-03	1.63	2.53E-06	0.80	8.19E-03	0.69	3.20E-02	-0.14	7.72E-01	-0.83	3.86E-03
LHFPL2	-0.22	8.73E-01	0.27	6.96E-01	1.10	3.39E-02	0.49	5.08E-01	1.32	9.94E-03	0.83	1.00E-01
LIMCH1	0.13	9.26E-01	0.39	4.81E-01	-0.97	4.25E-02	0.25	7.62E-01	-1.11	1.86E-02	-1.36	2.63E-03
LMNB1	-1.12	2.40E-01	-1.20	5.50E-02	0.72	3.84E-01	-0.08	9.65E-01	1.84	6.08E-03	1.91	2.88E-03
LMOD1	0.27	8.26E-01	-0.36	5.65E-01	-2.45	2.04E-05	-0.62	3.39E-01	-2.71	3.65E-06	-2.09	6.10E-05
LOC401097	0.79	6.73E-01	1.03	3.08E-01	-0.81	5.30E-01	0.24	9.13E-01	-1.60	1.10E-01	-1.84	4.49E-02
LOC402778	0.15	9.38E-01	-0.03	9.79E-01	-1.27	4.42E-02	-0.19	8.90E-01	-1.42	2.12E-02	-1.24	3.57E-02
LOC554202	-0.10	9.36E-01	-0.28	5.57E-01	1.16	3.58E-03	-0.18	8.03E-01	1.26	1.44E-03	1.44	2.34E-04
LOH3CR2A	-0.42	7.84E-01	0.05	9.68E-01	-1.33	5.62E-02	0.47	6.69E-01	-0.91	2.27E-01	-1.38	3.24E-02
LOXL3	-0.33	7.28E-01	0.81	5.50E-02	0.39	5.17E-01	1.14	1.83E-02	0.72	1.32E-01	-0.42	4.34E-01
LPAR1	0.02	9.78E-01	-0.33	9.49E-02	0.76	5.19E-04	-0.35	1.40E-01	0.74	5.37E-04	1.09	3.64E-06
LPPR4	1.11	2.75E-01	1.87	3.70E-03	-0.80	3.32E-01	0.76	4.21E-01	-1.91	5.99E-03	-2.68	1.57E-04
LPXN	-0.24	7.59E-01	0.18	7.30E-01	1.19	1.46E-03	0.41	3.90E-01	1.43	1.77E-04	1.02	3.28E-03
LRCH2	0.41	6.53E-01	0.08	9.24E-01	1.09	2.38E-02	-0.33	6.71E-01	0.68	1.88E-01	1.01	2.56E-02
LRRC32	-0.50	2.83E-01	0.50	1.02E-01	-1.33	1.18E-04	1.00	3.66E-03	-0.83	8.84E-03	-1.83	1.06E-06
LRRC49	0.37	7.23E-01	-0.29	6.66E-01	1.17	2.20E-02	-0.66	2.96E-01	0.80	1.34E-01	1.46	2.60E-03
LRRCC1	0.18	8.27E-01	-0.07	9.09E-01	1.06	2.67E-03	-0.25	6.57E-01	0.88	1.03E-02	1.13	8.01E-04
LRRFIP1	-0.16	9.20E-01	0.97	4.08E-02	0.98	6.33E-02	1.13	4.13E-02	1.14	2.64E-02	0.01	9.96E-01
LRRFIP1	0.18	9.30E-01	1.61	6.44E-03	1.26	5.61E-02	1.44	3.83E-02	1.09	1.05E-01	-0.35	7.12E-01

LRRFIP1	0.16	9.41E-01	1.39	2.98E-02	1.27	7.50E-02	1.22	1.19E-01	1.11	1.28E-01	-0.12	9.32E-01
LRRN3	0.20	9.13E-01	1.57	5.85E-03	-0.02	9.89E-01	1.36	4.17E-02	-0.23	8.33E-01	-1.59	7.09E-03
LSAMP	0.44	5.18E-01	-0.37	3.90E-01	-1.74	6.27E-05	-0.81	6.04E-02	-2.18	2.84E-06	-1.37	4.61E-04
LY6E	0.11	9.31E-01	1.28	8.51E-04	-0.26	6.71E-01	1.17	7.24E-03	-0.37	4.85E-01	-1.54	1.84E-04
LY75	0.03	9.85E-01	-0.09	9.08E-01	1.00	2.45E-02	-0.12	9.02E-01	0.96	2.77E-02	1.08	9.24E-03
LYN	-0.35	7.50E-01	-0.01	9.91E-01	-1.17	2.34E-02	0.34	6.92E-01	-0.82	1.24E-01	-1.16	1.66E-02
LYPD6B	-0.23	8.52E-01	-0.92	4.19E-02	-1.82	4.04E-04	-0.68	2.48E-01	-1.59	1.30E-03	-0.90	5.17E-02
MAFB	-0.32	8.64E-01	-0.42	6.55E-01	1.32	7.33E-02	-0.10	9.59E-01	1.64	2.12E-02	1.74	1.02E-02
MAGI1	-0.67	5.38E-02	-0.87	9.03E-04	-1.20	8.07E-05	-0.20	6.64E-01	-0.52	6.21E-02	-0.33	2.74E-01
MALL	-0.45	7.20E-01	-1.08	5.42E-02	1.32	3.10E-02	-0.64	4.45E-01	1.76	3.48E-03	2.40	1.05E-04
MAP3K5	-0.36	5.70E-01	-0.90	5.25E-03	0.19	7.31E-01	-0.54	1.90E-01	0.55	1.39E-01	1.09	1.36E-03
MAPK10	0.44	6.12E-01	0.13	8.67E-01	-0.65	2.12E-01	-0.31	6.88E-01	-1.10	1.95E-02	-0.78	8.81E-02
MAT2A	-0.01	9.93E-01	-0.66	9.57E-03	0.37	2.34E-01	-0.65	2.90E-02	0.38	2.08E-01	1.03	2.32E-04
MBOAT1	-0.18	8.91E-01	-0.17	7.99E-01	1.35	3.13E-03	0.00	9.97E-01	1.53	8.26E-04	1.53	5.43E-04
MBOAT2	-0.05	9.58E-01	-0.75	4.10E-03	0.78	6.76E-03	-0.70	2.05E-02	0.83	3.47E-03	1.53	2.63E-06
MCM4	-0.29	7.59E-01	-0.09	9.02E-01	0.83	6.06E-02	0.20	8.09E-01	1.12	9.76E-03	0.92	2.43E-02
MCM7	-0.86	2.96E-01	-0.91	8.57E-02	0.44	5.60E-01	-0.05	9.71E-01	1.30	1.97E-02	1.36	1.04E-02
MCM8	-0.36	7.84E-01	-0.06	9.55E-01	0.80	2.19E-01	0.30	7.74E-01	1.16	4.80E-02	0.86	1.43E-01
MEOX2	0.97	4.17E-01	0.54	5.63E-01	3.51	3.30E-05	-0.44	7.39E-01	2.54	9.87E-04	2.98	1.16E-04
MEST	-0.02	9.92E-01	0.06	9.38E-01	-1.23	3.61E-03	0.08	9.38E-01	-1.21	3.43E-03	-1.28	1.30E-03
MFAP4	1.25	1.46E-01	2.08	6.28E-04	0.86	2.30E-01	0.83	3.03E-01	-0.39	6.78E-01	-1.22	4.48E-02
MIR622	-0.21	8.88E-01	0.49	3.93E-01	-0.90	9.80E-02	0.70	2.79E-01	-0.69	2.32E-01	-1.39	5.14E-03
MKI67	-1.80	1.29E-01	-2.55	2.05E-03	0.49	7.22E-01	-0.75	5.79E-01	2.29	9.78E-03	3.04	5.19E-04
MKX	0.32	7.63E-01	0.09	9.14E-01	2.00	1.35E-04	-0.23	7.99E-01	1.68	6.86E-04	1.91	1.12E-04
MME	-0.99	6.46E-01	0.02	9.92E-01	2.45	3.53E-02	1.02	5.58E-01	3.44	2.81E-03	2.43	2.57E-02
MMP19	0.31	3.32E-01	0.46	2.03E-02	-1.33	6.40E-07	0.15	6.81E-01	-1.64	1.85E-08	-1.79	3.80E-09
MMP3	-0.04	9.87E-01	-0.21	8.48E-01	1.34	5.81E-02	-0.17	9.14E-01	1.38	4.65E-02	1.55	1.75E-02
MND1	-0.76	3.73E-01	-0.46	4.74E-01	0.65	3.24E-01	0.30	7.59E-01	1.41	1.11E-02	1.11	3.59E-02
MOXD1	0.02	9.96E-01	-0.04	9.68E-01	3.07	5.04E-06	-0.06	9.70E-01	3.06	4.22E-06	3.12	2.38E-06
MPP7	0.37	6.69E-01	0.23	7.08E-01	1.19	7.87E-03	-0.14	8.83E-01	0.82	6.60E-02	0.96	2.12E-02
MRVI1	0.23	7.46E-01	-0.15	7.63E-01	-0.78	2.09E-02	-0.38	4.01E-01	-1.00	2.64E-03	-0.63	4.73E-02
MSC	0.05	9.82E-01	1.10	4.35E-02	-0.61	4.03E-01	1.05	1.14E-01	-0.66	3.42E-01	-1.70	2.49E-03
MSX2	0.08	9.70E-01	0.79	2.21E-01	-0.59	4.74E-01	0.71	4.12E-01	-0.67	3.87E-01	-1.38	2.28E-02
MT1E	-0.57	2.85E-02	-0.92	3.48E-05	0.08	8.34E-01	-0.35	1.61E-01	0.65	2.40E-03	1.00	1.45E-05

MT1X	-1.00	3.63E-02	-0.65	7.27E-02	0.60	1.49E-01	0.35	5.50E-01	1.60	1.06E-04	1.25	8.99E-04
MT2A	-1.34	2.05E-05	-0.75	1.35E-03	0.34	2.27E-01	0.59	2.54E-02	1.67	1.38E-07	1.08	3.42E-05
MT2A	-1.34	2.05E-05	-0.79	8.36E-04	0.37	1.81E-01	0.55	4.21E-02	1.71	1.38E-07	1.16	1.71E-05
MT2A	-1.27	2.13E-05	-0.74	1.34E-03	0.40	1.31E-01	0.53	4.80E-02	1.67	1.38E-07	1.14	1.72E-05
MTSS1	-1.22	1.11E-01	-1.99	4.00E-04	-0.32	7.30E-01	-0.77	2.94E-01	0.91	1.41E-01	1.67	2.74E-03
MURC	0.04	9.79E-01	1.01	5.96E-03	0.12	8.70E-01	0.97	2.27E-02	0.08	9.15E-01	-0.88	1.95E-02
MX1	0.18	9.58E-01	3.35	5.01E-04	0.22	9.19E-01	3.17	3.50E-03	0.04	9.88E-01	-3.14	1.29E-03
MX2	0.28	9.20E-01	2.40	3.94E-03	-0.55	6.81E-01	2.12	2.75E-02	-0.83	4.71E-01	-2.95	8.19E-04
MXRA5	1.18	5.06E-01	2.23	2.03E-02	1.88	8.40E-02	1.05	4.83E-01	0.69	6.46E-01	-0.36	8.38E-01
MYEF2	-0.05	9.75E-01	-0.70	6.36E-02	0.31	5.70E-01	-0.65	1.59E-01	0.36	4.91E-01	1.01	8.11E-03
MYH10	0.02	9.86E-01	-0.38	1.84E-01	0.78	8.03E-03	-0.40	2.58E-01	0.76	8.97E-03	1.17	9.84E-05
MYH11	1.53	1.78E-01	2.36	2.39E-03	-0.05	9.79E-01	0.83	4.84E-01	-1.58	6.22E-02	-2.41	2.61E-03
MYL9	-0.01	9.92E-01	-0.15	7.19E-01	-1.76	1.15E-06	-0.13	8.12E-01	-1.74	1.18E-06	-1.61	2.38E-06
MYPN	-0.81	5.48E-01	-1.41	4.67E-02	-0.72	4.59E-01	-0.60	6.12E-01	0.09	9.55E-01	0.69	4.53E-01
N4BP2	-0.01	9.94E-01	-0.13	8.33E-01	0.93	1.57E-02	-0.11	8.93E-01	0.94	1.30E-02	1.06	3.81E-03
NALCN	-0.07	9.61E-01	-0.32	5.70E-01	-1.22	7.91E-03	-0.24	7.63E-01	-1.14	1.13E-02	-0.90	3.60E-02
NAMPT	-0.35	6.37E-01	-0.36	4.21E-01	0.93	1.90E-02	0.00	9.97E-01	1.29	1.34E-03	1.29	8.30E-04
NAMPT	-0.26	7.44E-01	-0.31	4.79E-01	0.94	1.36E-02	-0.05	9.62E-01	1.21	1.67E-03	1.26	7.49E-04
NAP1L3	0.61	2.41E-01	1.01	2.79E-03	-0.19	7.43E-01	0.40	4.14E-01	-0.80	2.82E-02	-1.20	7.81E-04
NASP	-0.36	6.02E-01	-0.13	8.24E-01	0.77	4.74E-02	0.23	7.19E-01	1.13	3.00E-03	0.90	1.23E-02
NAV2	-1.14	1.22E-02	-1.30	3.89E-04	0.80	3.92E-02	-0.16	8.29E-01	1.94	7.02E-06	2.10	1.90E-06
NCALD	0.07	9.24E-01	0.10	7.92E-01	1.40	2.72E-06	0.03	9.70E-01	1.33	4.19E-06	1.30	3.65E-06
NCAM1	0.44	7.79E-01	-0.14	9.08E-01	1.47	3.93E-02	-0.58	5.92E-01	1.03	1.71E-01	1.61	1.56E-02
NCAM2	-0.20	9.08E-01	0.41	5.48E-01	-2.46	7.75E-05	0.61	4.32E-01	-2.26	1.65E-04	-2.87	5.54E-06
NCAPG	-1.20	3.20E-01	-2.12	4.89E-03	0.58	6.15E-01	-0.92	4.06E-01	1.79	2.86E-02	2.70	7.64E-04
NCAPG2	-0.51	6.19E-01	-0.66	2.33E-01	0.45	5.41E-01	-0.15	8.98E-01	0.96	8.80E-02	1.11	3.22E-02
NCAPH	-0.63	6.85E-01	-0.91	2.53E-01	0.77	4.31E-01	-0.29	8.56E-01	1.40	8.00E-02	1.69	2.25E-02
NCKAP5	0.61	4.70E-01	0.21	7.85E-01	-0.66	2.52E-01	-0.40	6.14E-01	-1.27	1.24E-02	-0.87	7.87E-02
NCRNA00188	-0.37	2.38E-01	-1.10	5.06E-06	-0.40	7.38E-02	-0.73	2.38E-03	-0.04	9.38E-01	0.70	1.05E-03
NDC80	-0.80	5.62E-01	-1.35	6.08E-02	0.76	4.30E-01	-0.55	6.57E-01	1.56	4.35E-02	2.11	4.03E-03
NDRG1	-0.39	5.76E-01	-0.52	1.83E-01	0.60	1.54E-01	-0.12	8.84E-01	0.99	1.10E-02	1.12	2.92E-03
NDST1	0.32	4.65E-01	0.69	4.84E-03	-0.41	1.45E-01	0.37	2.56E-01	-0.73	5.71E-03	-1.10	6.15E-05
NEDD4L	-0.09	9.71E-01	-0.66	4.12E-01	0.74	4.08E-01	-0.57	6.10E-01	0.84	3.25E-01	1.41	4.15E-02
NEFM	-2.23	3.53E-02	-2.55	1.45E-03	-2.44	5.04E-03	-0.32	8.51E-01	-0.22	8.94E-01	0.11	9.52E-01

NEIL3	-0.36	7.48E-01	-0.86	9.06E-02	0.47	4.92E-01	-0.49	5.31E-01	0.84	1.35E-01	1.33	8.38E-03
NEK10	-0.93	2.96E-01	-1.68	2.84E-03	-0.43	6.22E-01	-0.75	3.40E-01	0.50	5.31E-01	1.25	2.96E-02
NEK2	-0.91	3.12E-01	-1.21	3.29E-02	0.27	8.01E-01	-0.30	7.95E-01	1.18	5.71E-02	1.47	1.11E-02
NEK6	-0.31	2.90E-01	-0.23	2.84E-01	-1.09	4.72E-06	0.09	8.22E-01	-0.78	2.43E-04	-0.86	4.38E-05
NEK7	0.27	8.01E-01	0.34	5.60E-01	-0.71	1.65E-01	0.06	9.59E-01	-0.98	3.74E-02	-1.04	1.93E-02
NFATC2	1.00	3.03E-01	1.55	1.08E-02	0.76	3.35E-01	0.54	5.92E-01	-0.24	8.33E-01	-0.78	2.75E-01
NFIA	0.42	6.23E-01	0.34	5.43E-01	1.20	9.44E-03	-0.09	9.37E-01	0.78	1.01E-01	0.87	4.86E-02
NFIB	0.61	6.28E-01	0.77	2.67E-01	2.81	1.20E-04	0.16	9.20E-01	2.20	1.30E-03	2.05	1.65E-03
NFKBIA	-0.35	6.09E-01	-0.33	4.28E-01	1.09	3.62E-03	0.02	9.86E-01	1.44	1.93E-04	1.42	1.42E-04
NHSL1	0.09	9.54E-01	0.20	7.54E-01	1.50	1.41E-03	0.11	9.12E-01	1.41	2.07E-03	1.30	2.84E-03
NLGN1	0.25	8.82E-01	-0.38	6.19E-01	2.38	2.28E-04	-0.63	4.46E-01	2.13	5.71E-04	2.77	1.83E-05
NLRP10	0.19	8.37E-01	0.91	8.69E-03	-0.21	7.17E-01	0.72	8.64E-02	-0.40	3.77E-01	-1.12	2.09E-03
NME5	0.28	6.38E-01	-0.07	9.02E-01	-1.07	1.09E-03	-0.35	4.17E-01	-1.35	6.99E-05	-1.01	1.04E-03
NMI	0.25	7.84E-01	1.18	1.74E-03	0.77	6.13E-02	0.93	3.10E-02	0.52	2.44E-01	-0.41	3.83E-01
NMT2	-0.37	4.95E-01	-1.08	4.97E-04	-0.38	3.22E-01	-0.71	4.21E-02	-0.01	9.94E-01	0.70	2.20E-02
NPC1	-0.89	2.32E-01	-0.84	9.18E-02	0.18	8.52E-01	0.05	9.73E-01	1.07	4.22E-02	1.02	4.08E-02
NR2F2	0.16	8.18E-01	0.14	7.31E-01	-1.17	1.58E-04	-0.02	9.84E-01	-1.33	2.56E-05	-1.31	1.96E-05
NR3C2	0.20	7.65E-01	0.48	1.12E-01	1.22	2.53E-04	0.27	5.67E-01	1.02	1.35E-03	0.74	1.17E-02
NR4A1	0.22	7.02E-01	-0.19	5.87E-01	0.98	7.81E-04	-0.41	2.28E-01	0.77	6.19E-03	1.17	5.68E-05
NR4A2	-0.37	8.11E-01	-0.28	7.73E-01	1.87	5.42E-03	0.09	9.59E-01	2.23	9.32E-04	2.15	8.70E-04
NR4A3	-0.19	8.64E-01	-0.14	8.27E-01	1.17	5.75E-03	0.05	9.67E-01	1.35	1.35E-03	1.31	1.19E-03
NRP2	-0.30	8.12E-01	-0.81	1.17E-01	1.11	4.28E-02	-0.51	5.12E-01	1.41	8.94E-03	1.92	3.41E-04
NTRK2	0.58	4.62E-01	1.00	2.30E-02	-0.87	7.79E-02	0.42	5.59E-01	-1.45	2.64E-03	-1.87	1.29E-04
NTRK3	0.60	3.04E-01	1.31	6.01E-04	-0.38	4.55E-01	0.71	1.19E-01	-0.98	1.36E-02	-1.69	5.20E-05
NUAK1	-0.02	9.95E-01	0.17	8.52E-01	-1.74	2.29E-03	0.19	8.73E-01	-1.72	2.12E-03	-1.91	5.16E-04
NUF2	-1.30	3.57E-01	-1.64	5.96E-02	0.81	5.08E-01	-0.33	8.61E-01	2.11	2.26E-02	2.45	5.72E-03
NUSAP1	-1.02	3.00E-01	-1.73	5.11E-03	0.72	3.79E-01	-0.70	4.50E-01	1.75	9.40E-03	2.45	2.76E-04
OAS1	0.39	8.69E-01	2.99	4.91E-04	1.68	6.76E-02	2.60	7.05E-03	1.29	1.82E-01	-1.31	1.48E-01
OAS2	0.50	6.06E-01	2.57	8.22E-06	-0.35	6.42E-01	2.07	5.90E-04	-0.86	1.20E-01	-2.92	1.78E-06
OAS3	0.12	9.35E-01	1.70	2.22E-04	0.23	7.60E-01	1.58	2.28E-03	0.11	9.01E-01	-1.47	1.22E-03
ODZ3	-0.17	9.32E-01	0.77	2.41E-01	1.65	1.14E-02	0.94	2.22E-01	1.82	4.81E-03	0.88	1.89E-01
OGFRL1	-0.25	5.82E-01	-0.24	3.89E-01	1.17	2.93E-05	0.01	9.87E-01	1.42	1.82E-06	1.41	1.35E-06
OGN	1.56	4.92E-01	1.36	3.47E-01	3.57	8.85E-03	-0.20	9.56E-01	2.01	1.64E-01	2.21	9.50E-02
OLFM2	0.54	2.30E-01	1.70	2.63E-06	-0.23	6.01E-01	1.16	1.17E-03	-0.77	1.46E-02	-1.94	4.26E-07

OSTN	0.30	8.08E-01	1.20	1.28E-02	-0.07	9.54E-01	0.90	1.33E-01	-0.37	6.25E-01	-1.27	1.10E-02
OXTR	0.54	5.63E-01	0.80	1.14E-01	-0.41	5.68E-01	0.26	7.89E-01	-0.95	7.55E-02	-1.21	1.50E-02
P4HA2	-0.38	4.19E-01	0.48	9.18E-02	-0.61	4.27E-02	0.86	7.24E-03	-0.23	5.66E-01	-1.09	2.62E-04
PAICS	-0.65	1.48E-01	-0.25	5.36E-01	0.55	1.18E-01	0.40	3.54E-01	1.20	4.94E-04	0.80	1.13E-02
PALMD	1.04	3.45E-01	1.88	5.02E-03	-1.02	2.07E-01	0.84	3.87E-01	-2.06	4.71E-03	-2.90	1.05E-04
PAMR1	0.17	8.75E-01	-1.40	2.98E-04	0.37	4.74E-01	-1.57	5.90E-04	0.20	7.57E-01	1.77	2.75E-05
PAPPA	0.00	9.99E-01	0.63	5.58E-02	-1.51	9.37E-05	0.63	1.13E-01	-1.51	7.36E-05	-2.14	4.77E-07
PAPPA	-0.07	9.50E-01	0.43	1.64E-01	-1.34	8.85E-05	0.49	1.74E-01	-1.27	1.27E-04	-1.76	1.30E-06
PAPPA2	-0.42	7.21E-01	-0.20	8.21E-01	0.80	2.06E-01	0.22	8.45E-01	1.23	3.22E-02	1.00	7.08E-02
PAPSS1	0.31	3.22E-01	0.29	1.64E-01	-0.83	2.95E-04	-0.02	9.72E-01	-1.14	4.25E-06	-1.12	3.80E-06
PARP14	0.27	7.74E-01	1.44	3.96E-04	0.43	4.18E-01	1.17	9.30E-03	0.16	8.25E-01	-1.01	1.10E-02
PARP8	0.32	5.36E-01	0.38	1.90E-01	-2.64	1.52E-09	0.06	9.27E-01	-2.96	2.32E-10	-3.02	2.63E-10
PARP9	0.41	6.21E-01	1.80	6.12E-05	0.51	3.26E-01	1.39	3.43E-03	0.10	9.03E-01	-1.29	2.36E-03
PART1	-0.09	9.18E-01	-0.03	9.50E-01	1.35	2.04E-05	0.06	9.33E-01	1.43	6.21E-06	1.38	6.95E-06
PBK	-1.42	4.27E-01	-2.35	2.27E-02	0.67	6.95E-01	-0.93	5.91E-01	2.09	6.67E-02	3.02	4.85E-03
PCDH10	-0.16	9.31E-01	0.36	6.35E-01	-1.16	5.48E-02	0.52	5.59E-01	-1.00	1.01E-01	-1.52	6.64E-03
PCOLCE2	0.56	6.08E-01	0.48	4.93E-01	1.41	1.89E-02	-0.09	9.57E-01	0.84	1.90E-01	0.93	1.14E-01
PCSK1	-0.50	5.94E-01	-0.70	1.69E-01	1.68	1.59E-03	-0.20	8.50E-01	2.19	7.82E-05	2.38	1.71E-05
PCSK5	0.43	4.37E-02	1.32	2.05E-08	-0.11	6.41E-01	0.90	1.76E-05	-0.54	1.64E-03	-1.44	3.04E-09
PDCD1LG2	-0.01	9.98E-01	0.91	8.40E-02	-0.41	5.94E-01	0.92	1.50E-01	-0.40	6.03E-01	-1.32	1.17E-02
PDE1C	0.07	9.60E-01	-0.27	5.77E-01	-1.04	9.41E-03	-0.34	5.75E-01	-1.10	5.16E-03	-0.76	4.23E-02
PDE3A	-0.31	7.89E-01	-0.39	5.33E-01	-2.76	5.89E-06	-0.08	9.55E-01	-2.45	2.13E-05	-2.37	2.06E-05
PDE4B	-0.25	8.56E-01	0.38	5.57E-01	0.88	1.20E-01	0.63	3.67E-01	1.13	3.48E-02	0.49	4.38E-01
PDE5A	-0.24	8.85E-01	-1.44	8.82E-03	-3.31	3.72E-06	-1.19	7.04E-02	-3.07	7.02E-06	-1.87	1.26E-03
PDGFRA	-0.13	8.83E-01	-0.56	4.94E-02	0.50	1.21E-01	-0.43	2.44E-01	0.63	4.13E-02	1.06	4.74E-04
PDGFRL	0.13	9.75E-01	0.16	9.37E-01	2.42	4.29E-02	0.03	9.92E-01	2.29	5.35E-02	2.26	4.38E-02
PDLIM1	0.29	9.26E-01	0.43	7.74E-01	-1.85	7.67E-02	0.14	9.58E-01	-2.13	3.47E-02	-2.27	1.75E-02
PEG10	0.13	9.28E-01	0.18	7.96E-01	1.36	3.43E-03	0.05	9.65E-01	1.24	6.34E-03	1.19	6.13E-03
PENK	0.19	7.24E-01	-1.02	9.65E-05	0.01	9.83E-01	-1.21	9.28E-05	-0.18	6.34E-01	1.03	1.09E-04
PHLDA1	-0.07	9.59E-01	-0.94	7.09E-03	0.07	9.36E-01	-0.87	3.18E-02	0.13	8.45E-01	1.01	5.58E-03
PIK3R3	1.33	5.42E-03	1.43	2.63E-04	0.74	7.20E-02	0.09	9.20E-01	-0.59	1.68E-01	-0.68	7.84E-02
PITPNC1	-0.60	2.34E-01	-0.69	3.55E-02	1.19	1.27E-03	-0.09	9.11E-01	1.79	8.62E-06	1.88	3.48E-06
PLA2G16	-0.72	3.04E-01	-1.04	1.76E-02	-1.43	3.51E-03	-0.32	6.85E-01	-0.71	1.64E-01	-0.39	5.16E-01
PLA2G4A	0.38	6.55E-01	1.09	7.09E-03	0.37	5.37E-01	0.71	1.62E-01	-0.01	9.92E-01	-0.72	9.37E-02

PLAU	0.71	2.67E-01	1.01	1.21E-02	-0.06	9.50E-01	0.31	6.71E-01	-0.77	8.95E-02	-1.08	1.01E-02
PLAUR	-0.69	3.05E-01	-0.41	4.26E-01	0.41	5.03E-01	0.28	7.25E-01	1.10	1.71E-02	0.82	6.63E-02
PLBD1	0.33	5.26E-01	0.49	8.02E-02	1.06	6.75E-04	0.16	7.52E-01	0.73	1.40E-02	0.56	4.69E-02
PLCE1	0.74	4.20E-01	0.86	1.24E-01	1.47	1.19E-02	0.12	9.30E-01	0.73	2.64E-01	0.61	3.57E-01
PLCL2	0.32	7.65E-01	-0.03	9.79E-01	1.10	2.35E-02	-0.34	6.63E-01	0.79	1.18E-01	1.13	1.34E-02
PLEKHH2	0.30	6.55E-01	0.74	2.20E-02	1.14	1.76E-03	0.44	3.32E-01	0.84	1.71E-02	0.40	3.08E-01
PLIN2	-1.26	5.59E-03	-1.82	1.09E-05	-0.04	9.66E-01	-0.56	2.15E-01	1.22	1.55E-03	1.79	1.43E-05
PLK1	-1.13	2.93E-01	-1.55	2.13E-02	0.07	9.68E-01	-0.42	7.41E-01	1.20	1.18E-01	1.62	2.00E-02
PLK2	0.48	4.93E-01	-0.24	6.69E-01	1.21	4.36E-03	-0.72	1.30E-01	0.73	8.80E-02	1.45	4.59E-04
PLK4	-0.43	8.55E-01	-0.74	4.82E-01	1.18	2.52E-01	-0.32	8.64E-01	1.61	8.51E-02	1.93	2.55E-02
PLXDC1	1.17	4.13E-01	1.77	3.43E-02	0.54	6.97E-01	0.60	6.91E-01	-0.64	6.20E-01	-1.24	1.86E-01
PLXND1	-0.13	8.18E-01	0.01	9.73E-01	-1.13	2.00E-05	0.14	7.22E-01	-1.01	6.35E-05	-1.15	7.17E-06
PMAIP1	-0.05	9.86E-01	-0.11	9.24E-01	3.04	4.40E-05	-0.06	9.73E-01	3.08	2.62E-05	3.15	1.36E-05
PODN	0.21	8.98E-01	0.74	1.64E-01	-0.97	8.35E-02	0.54	4.92E-01	-1.18	2.98E-02	-1.72	1.14E-03
PODXL	-1.69	1.26E-02	-1.92	4.07E-04	2.01	6.39E-04	-0.23	8.37E-01	3.70	1.91E-07	3.93	5.75E-08
POLQ	-0.37	7.06E-01	-0.42	4.45E-01	0.62	2.65E-01	-0.05	9.69E-01	0.99	4.26E-02	1.04	2.37E-02
PPAP2B	0.03	9.84E-01	-0.69	2.06E-02	0.85	9.44E-03	-0.71	4.07E-02	0.82	1.11E-02	1.53	1.27E-05
PPP1R14A	0.31	8.23E-01	0.18	8.43E-01	-1.09	6.89E-02	-0.13	9.26E-01	-1.40	1.58E-02	-1.27	2.09E-02
PPP2R3A	-0.05	9.56E-01	1.12	2.26E-05	0.99	2.28E-04	1.17	9.28E-05	1.04	1.05E-04	-0.14	7.10E-01
PRC1	-1.22	2.05E-01	-2.14	1.04E-03	0.11	9.39E-01	-0.92	2.85E-01	1.33	5.66E-02	2.25	8.60E-04
PRELP	0.57	5.71E-01	0.23	7.80E-01	1.47	9.83E-03	-0.34	7.25E-01	0.90	1.31E-01	1.24	2.07E-02
PRG4	0.25	9.62E-01	-0.89	6.73E-01	3.89	1.47E-02	-1.14	6.56E-01	3.64	2.06E-02	4.78	1.69E-03
PRICKLE1	0.12	9.01E-01	0.10	8.47E-01	-2.50	1.24E-08	-0.02	9.85E-01	-2.62	4.57E-09	-2.60	4.07E-09
PRIM1	-0.26	8.72E-01	-0.39	6.16E-01	0.99	1.27E-01	-0.13	9.30E-01	1.25	4.12E-02	1.38	1.72E-02
PRKAB2	0.37	4.16E-01	0.23	4.95E-01	-1.03	6.09E-04	-0.14	7.93E-01	-1.40	1.26E-05	-1.26	3.08E-05
PRR11	-1.06	3.82E-01	-1.68	1.96E-02	0.42	7.36E-01	-0.62	6.09E-01	1.48	6.41E-02	2.10	5.10E-03
PRSS35	0.29	4.64E-01	0.84	4.02E-04	-0.20	5.51E-01	0.54	4.05E-02	-0.49	4.55E-02	-1.03	5.27E-05
PRTFDC1	-0.13	9.07E-01	-0.38	3.32E-01	0.78	3.33E-02	-0.25	6.75E-01	0.91	1.20E-02	1.16	1.05E-03
PSG4	-0.83	1.96E-01	-1.30	2.66E-03	-0.06	9.51E-01	-0.47	4.81E-01	0.77	1.13E-01	1.23	5.37E-03
PSG5	-1.29	2.35E-01	-2.27	1.54E-03	-0.27	8.45E-01	-0.98	3.15E-01	1.02	2.28E-01	2.00	6.13E-03
PSG7	-1.15	2.56E-01	-1.66	9.89E-03	-0.26	8.40E-01	-0.51	6.48E-01	0.89	2.60E-01	1.41	3.57E-02
PSIP1	0.07	9.53E-01	-0.53	1.14E-01	1.04	3.73E-03	-0.60	1.30E-01	0.96	5.71E-03	1.57	2.47E-05
PTER	-0.22	7.67E-01	-0.51	1.11E-01	0.90	8.07E-03	-0.29	5.61E-01	1.11	1.09E-03	1.41	5.30E-05
PTGES	-0.58	4.18E-01	-0.75	7.86E-02	-2.47	3.72E-06	-0.17	8.51E-01	-1.89	8.71E-05	-1.72	1.63E-04

PTGFR	0.31	6.77E-01	-0.17	7.51E-01	1.12	3.59E-03	-0.48	3.17E-01	0.81	3.01E-02	1.29	5.19E-04
PTGFRN	0.45	2.90E-01	0.15	7.05E-01	1.10	3.48E-04	-0.30	4.65E-01	0.65	2.35E-02	0.95	8.04E-04
PTPLAD2	0.29	6.41E-01	-0.35	3.14E-01	0.84	1.08E-02	-0.64	7.86E-02	0.55	1.04E-01	1.19	2.83E-04
PTTG1	-0.99	3.04E-01	-1.56	9.18E-03	0.17	8.99E-01	-0.58	5.64E-01	1.15	8.64E-02	1.73	5.58E-03
PTX3	-0.83	1.96E-01	-1.42	1.14E-03	-1.08	2.10E-02	-0.59	3.16E-01	-0.25	7.23E-01	0.34	5.81E-01
RAB27B	-0.83	6.60E-01	-1.14	2.61E-01	-2.84	4.80E-03	-0.31	8.83E-01	-2.00	4.30E-02	-1.70	7.62E-02
RAB38	0.12	8.70E-01	0.31	2.60E-01	1.89	9.40E-08	0.19	6.57E-01	1.78	1.87E-07	1.58	8.22E-07
RAB3B	-1.02	2.51E-01	-1.85	1.44E-03	-0.05	9.69E-01	-0.83	2.81E-01	0.97	1.40E-01	1.80	2.52E-03
RAC2	-0.77	3.88E-01	-1.55	3.44E-03	-1.86	1.71E-03	-0.79	2.67E-01	-1.09	5.98E-02	-0.31	7.16E-01
RACGAP1	-0.54	4.82E-01	-0.93	2.77E-02	0.33	6.13E-01	-0.39	5.77E-01	0.86	6.19E-02	1.25	4.03E-03
RAD51AP1	-0.67	7.02E-01	-1.02	2.50E-01	0.79	4.81E-01	-0.35	8.39E-01	1.46	1.05E-01	1.81	2.78E-02
RALGPS2	-0.17	8.86E-01	-1.01	8.27E-03	0.56	2.37E-01	-0.84	6.75E-02	0.73	9.22E-02	1.57	2.07E-04
RANBP3L	0.65	5.89E-01	1.13	6.89E-02	-0.25	8.34E-01	0.48	6.54E-01	-0.90	2.16E-01	-1.37	2.79E-02
RASA4	0.68	2.82E-01	0.16	8.14E-01	1.77	1.42E-04	-0.52	3.50E-01	1.09	1.09E-02	1.61	2.04E-04
RASSF9	0.44	5.43E-01	0.45	2.97E-01	-1.12	7.32E-03	0.01	9.94E-01	-1.56	2.83E-04	-1.57	1.70E-04
RBL1	-0.55	5.20E-01	-0.71	1.42E-01	0.56	3.39E-01	-0.16	8.79E-01	1.11	2.39E-02	1.27	6.79E-03
RBM8A	0.56	3.81E-01	0.76	4.71E-02	1.06	1.04E-02	0.20	7.87E-01	0.51	2.77E-01	0.30	5.74E-01
RBP4	0.21	8.40E-01	0.37	4.26E-01	4.23	1.95E-10	0.16	8.42E-01	4.03	3.89E-10	3.86	9.62E-10
RCAN2	0.43	6.54E-01	-0.28	6.86E-01	-0.59	3.24E-01	-0.71	2.52E-01	-1.02	4.43E-02	-0.31	6.59E-01
RDH10	0.48	6.92E-01	1.31	1.83E-02	0.84	2.09E-01	0.83	2.64E-01	0.36	6.88E-01	-0.47	5.48E-01
REV3L	0.19	8.83E-01	0.10	8.96E-01	1.04	2.45E-02	-0.09	9.36E-01	0.85	6.68E-02	0.94	3.07E-02
RFC3	-0.50	6.38E-01	-0.38	5.79E-01	0.79	1.98E-01	0.11	9.31E-01	1.29	2.04E-02	1.17	2.64E-02
RGNEF	-0.35	5.44E-01	-0.62	4.09E-02	0.43	2.45E-01	-0.27	5.93E-01	0.78	1.68E-02	1.06	9.95E-04
RGS2	-0.27	7.88E-01	-0.15	8.23E-01	1.58	5.72E-04	0.11	9.07E-01	1.85	7.91E-05	1.73	1.07E-04
RHOJ	0.92	1.59E-01	1.02	2.26E-02	0.62	2.62E-01	0.10	9.32E-01	-0.30	6.79E-01	-0.40	5.16E-01
RNF125	0.07	9.18E-01	-0.11	7.10E-01	1.00	3.24E-05	-0.18	5.91E-01	0.93	6.22E-05	1.11	4.28E-06
RNF141	-0.23	5.24E-01	-0.54	5.32E-03	-1.00	2.34E-05	-0.30	2.27E-01	-0.77	3.89E-04	-0.47	1.89E-02
RNF150	0.06	9.71E-01	0.19	7.90E-01	-1.04	3.16E-02	0.13	9.00E-01	-1.10	2.09E-02	-1.23	6.78E-03
RNF180	-0.01	9.98E-01	0.19	7.45E-01	-2.07	1.51E-05	0.20	7.95E-01	-2.06	1.02E-05	-2.26	2.30E-06
RNF19B	0.51	2.32E-01	1.15	1.28E-04	0.86	5.34E-03	0.64	5.07E-02	0.34	3.30E-01	-0.30	4.03E-01
ROR1	0.57	6.35E-02	1.11	1.66E-05	0.29	3.09E-01	0.53	3.80E-02	-0.28	3.07E-01	-0.82	5.63E-04
RPS4Y1	-0.09	9.79E-01	-0.04	9.83E-01	4.64	2.26E-05	0.05	9.86E-01	4.73	1.23E-05	4.68	9.54E-06
RSAD2	0.06	9.76E-01	1.94	5.15E-04	0.13	9.14E-01	1.88	3.17E-03	0.07	9.54E-01	-1.81	1.37E-03
RUNX2	-0.54	4.75E-01	-0.51	2.86E-01	-1.28	5.48E-03	0.03	9.81E-01	-0.75	1.15E-01	-0.78	7.77E-02

SAMD5	0.34	6.22E-01	0.55	1.21E-01	-0.50	2.28E-01	0.21	7.42E-01	-0.84	2.26E-02	-1.05	2.98E-03
SAMD9	-0.06	9.68E-01	1.11	5.48E-03	0.22	7.59E-01	1.17	1.15E-02	0.27	6.67E-01	-0.89	3.08E-02
SAMD9L	0.47	2.82E-01	1.51	4.48E-06	0.51	9.89E-02	1.04	1.80E-03	0.04	9.42E-01	-1.00	6.63E-04
SATB2	0.13	9.32E-01	0.45	3.82E-01	-1.07	2.53E-02	0.33	6.70E-01	-1.20	1.13E-02	-1.52	9.51E-04
SBSN	-1.09	1.89E-01	-1.22	2.83E-02	-0.03	9.85E-01	-0.13	9.27E-01	1.06	8.63E-02	1.19	3.75E-02
SC4MOL	1.30	1.28E-02	1.19	3.10E-03	0.36	5.41E-01	-0.11	9.13E-01	-0.94	3.01E-02	-0.83	4.46E-02
SC5DL	0.76	1.42E-01	1.03	4.03E-03	0.30	5.70E-01	0.26	6.77E-01	-0.46	2.98E-01	-0.73	4.93E-02
SCARA3	-0.15	8.83E-01	-0.43	2.58E-01	2.00	4.85E-06	-0.28	6.37E-01	2.16	1.46E-06	2.44	1.38E-07
SCARNA9L	-0.28	7.66E-01	-0.33	5.18E-01	0.83	5.54E-02	-0.05	9.65E-01	1.11	9.01E-03	1.16	4.21E-03
SCG2	-0.36	2.48E-01	0.55	5.11E-03	-0.48	2.86E-02	0.91	3.21E-04	-0.12	7.14E-01	-1.03	1.12E-05
SCG5	0.07	9.61E-01	-0.48	3.24E-01	0.53	3.25E-01	-0.55	3.51E-01	0.46	4.14E-01	1.01	1.94E-02
SCRG1	0.63	7.79E-01	1.52	1.18E-01	2.75	6.89E-03	0.89	5.62E-01	2.12	3.49E-02	1.23	2.51E-01
SCUBE3	-0.49	7.46E-01	-0.17	8.91E-01	-1.95	7.12E-03	0.33	8.08E-01	-1.46	4.29E-02	-1.78	8.62E-03
SDC2	0.33	2.80E-01	0.61	1.68E-03	-0.45	3.24E-02	0.28	2.70E-01	-0.78	3.27E-04	-1.06	5.09E-06
SELENBP1	0.13	8.99E-01	-0.41	2.39E-01	-0.93	7.91E-03	-0.54	1.78E-01	-1.06	2.36E-03	-0.52	1.36E-01
SEMA3A	0.30	9.38E-01	0.75	6.43E-01	-2.37	6.11E-02	0.45	8.59E-01	-2.67	3.00E-02	-3.11	7.79E-03
SEPT6	-0.08	9.20E-01	-0.11	7.63E-01	1.24	2.26E-05	-0.03	9.59E-01	1.32	7.02E-06	1.36	3.65E-06
SEPW1	0.65	1.11E-01	0.23	5.27E-01	-0.88	5.24E-03	-0.42	2.78E-01	-1.53	1.08E-05	-1.11	3.15E-04
SERPINB2	-0.01	9.98E-01	1.32	2.39E-01	-2.95	8.21E-03	1.34	3.51E-01	-2.94	7.69E-03	-4.28	1.42E-04
SERPINF1	0.79	5.39E-01	1.28	6.18E-02	1.56	3.58E-02	0.49	6.88E-01	0.77	3.87E-01	0.28	8.14E-01
SERTAD4	0.63	5.84E-01	0.83	1.89E-01	-1.46	2.33E-02	0.20	8.83E-01	-2.09	1.24E-03	-2.29	3.16E-04
SESN3	0.35	6.45E-01	0.05	9.47E-01	-0.82	4.72E-02	-0.30	6.33E-01	-1.17	3.77E-03	-0.86	2.37E-02
SFRP2	0.25	9.23E-01	2.15	5.34E-03	1.68	4.99E-02	1.90	3.53E-02	1.43	1.00E-01	-0.47	7.04E-01
SGCD	0.54	2.43E-01	0.75	1.18E-02	1.04	2.00E-03	0.21	7.02E-01	0.50	1.51E-01	0.29	4.68E-01
SGK1	-0.11	8.26E-01	-0.13	6.23E-01	0.92	4.97E-05	-0.02	9.70E-01	1.03	8.62E-06	1.05	4.65E-06
SGOL1	-0.76	5.42E-01	-0.87	2.30E-01	0.87	2.93E-01	-0.10	9.57E-01	1.63	2.12E-02	1.73	1.01E-02
SGOL2	-0.26	8.30E-01	-0.12	8.87E-01	0.90	8.47E-02	0.14	8.99E-01	1.16	2.09E-02	1.02	3.31E-02
SH2D4A	-0.77	1.57E-01	-0.56	1.59E-01	0.27	6.41E-01	0.21	7.72E-01	1.04	9.94E-03	0.83	3.01E-02
SH3BGRL2	0.33	6.78E-01	-0.08	9.08E-01	-0.93	2.20E-02	-0.41	4.74E-01	-1.26	1.98E-03	-0.85	2.56E-02
SHOX	0.13	8.72E-01	-0.06	9.10E-01	1.23	1.42E-04	-0.19	7.12E-01	1.10	3.80E-04	1.29	3.94E-05
SHOX	0.13	8.72E-01	-0.06	9.10E-01	1.23	1.42E-04	-0.19	7.12E-01	1.10	3.80E-04	1.29	3.94E-05
SIDT2	0.05	9.61E-01	0.07	8.97E-01	-1.28	1.18E-04	0.02	9.87E-01	-1.33	6.14E-05	-1.34	3.08E-05
SIRPA	-0.26	7.81E-01	-0.29	5.76E-01	0.95	2.34E-02	-0.03	9.82E-01	1.20	3.60E-03	1.23	1.99E-03
SKA1	-0.30	7.28E-01	-0.49	2.35E-01	0.54	2.34E-01	-0.19	7.95E-01	0.84	3.93E-02	1.03	7.41E-03

SKA3	-0.59	6.98E-01	-1.11	1.29E-01	1.02	2.28E-01	-0.52	6.73E-01	1.61	3.31E-02	2.13	3.24E-03
SLC14A1	-0.03	9.95E-01	-3.03	4.19E-04	-1.38	1.50E-01	-3.00	2.34E-03	-1.35	1.53E-01	1.65	5.37E-02
SLC16A9	0.25	6.96E-01	-0.09	8.58E-01	1.23	3.46E-04	-0.35	4.32E-01	0.97	2.64E-03	1.32	7.34E-05
SLC1A3	1.15	1.61E-01	1.46	8.99E-03	0.74	3.01E-01	0.31	7.81E-01	-0.41	6.37E-01	-0.72	2.75E-01
SLC22A3	-0.93	2.43E-01	-0.55	3.65E-01	-1.80	1.88E-03	0.38	6.83E-01	-0.87	1.43E-01	-1.25	1.87E-02
SLC25A20	-0.06	9.54E-01	0.31	3.65E-01	-0.73	2.40E-02	0.38	3.66E-01	-0.66	3.81E-02	-1.04	8.48E-04
SLC2A1	-0.55	3.38E-01	-0.19	7.32E-01	-1.15	3.60E-03	0.37	5.11E-01	-0.59	1.46E-01	-0.96	8.49E-03
SLC35F2	-0.11	9.01E-01	-0.16	6.97E-01	1.17	2.73E-04	-0.05	9.48E-01	1.28	7.63E-05	1.33	2.98E-05
SLC38A1	-0.25	8.89E-01	-0.25	7.88E-01	1.31	4.03E-02	0.00	9.98E-01	1.56	1.27E-02	1.56	8.74E-03
SLC39A8	-0.04	9.85E-01	0.73	9.94E-02	1.35	4.30E-03	0.77	1.52E-01	1.39	2.90E-03	0.62	1.96E-01
SLC41A2	0.42	2.34E-01	1.01	7.32E-05	0.23	4.77E-01	0.59	2.90E-02	-0.19	5.71E-01	-0.78	1.32E-03
SLC43A3	-1.09	9.47E-02	-1.77	2.74E-04	1.47	3.84E-03	-0.68	2.69E-01	2.57	6.90E-06	3.25	1.45E-07
SLC44A2	0.22	7.04E-01	0.36	2.08E-01	-1.17	1.56E-04	0.14	7.88E-01	-1.39	1.34E-05	-1.54	2.89E-06
SLC46A3	0.11	9.49E-01	0.39	5.18E-01	-0.82	1.19E-01	0.28	7.51E-01	-0.93	6.70E-02	-1.21	1.07E-02
SLC7A2	-0.04	9.86E-01	0.02	9.85E-01	1.33	4.60E-02	0.07	9.69E-01	1.37	3.55E-02	1.31	3.56E-02
SLC9A9	0.55	7.24E-01	0.01	9.96E-01	1.48	5.27E-02	-0.54	6.54E-01	0.93	2.70E-01	1.48	3.84E-02
SLIT2	0.27	8.74E-01	1.25	3.37E-02	1.67	9.54E-03	0.98	1.94E-01	1.40	2.84E-02	0.42	6.35E-01
SLIT3	0.66	3.39E-01	1.14	6.87E-03	0.83	8.29E-02	0.48	4.52E-01	0.17	8.35E-01	-0.32	6.13E-01
SMAD3	-0.19	7.24E-01	-1.12	3.10E-05	0.05	9.25E-01	-0.93	1.33E-03	0.24	4.61E-01	1.17	2.05E-05
SMAD9	-0.66	7.57E-02	-0.65	1.28E-02	0.57	5.36E-02	0.00	9.97E-01	1.22	7.41E-05	1.22	4.71E-05
SMC2	-0.48	4.83E-01	-0.31	5.28E-01	0.53	2.58E-01	0.17	8.31E-01	1.00	1.36E-02	0.84	3.13E-02
SMC4	-0.25	5.59E-01	-0.22	4.15E-01	0.83	1.09E-03	0.03	9.61E-01	1.09	4.70E-05	1.06	3.94E-05
SMOC2	0.64	8.24E-01	1.45	2.37E-01	-1.49	2.89E-01	0.81	6.86E-01	-2.13	8.69E-02	-2.94	1.03E-02
SNORA42	-0.09	9.35E-01	-0.69	2.96E-02	-1.19	8.76E-04	-0.60	1.22E-01	-1.10	1.55E-03	-0.50	1.48E-01
SNORA5A	-0.32	7.62E-01	-0.37	5.26E-01	-1.15	1.90E-02	-0.05	9.71E-01	-0.83	9.88E-02	-0.78	1.02E-01
SNORA75	-0.56	5.01E-01	-1.05	2.06E-02	-1.98	1.95E-04	-0.49	4.94E-01	-1.42	3.98E-03	-0.93	4.74E-02
SNORD116-17	0.27	6.47E-01	0.01	9.86E-01	-0.81	8.78E-03	-0.25	5.88E-01	-1.08	6.19E-04	-0.82	4.91E-03
SNORD116-17	0.27	6.47E-01	0.01	9.86E-01	-0.81	8.78E-03	-0.25	5.88E-01	-1.08	6.19E-04	-0.82	4.91E-03
SNORD116-2	0.64	1.55E-01	-0.12	8.22E-01	-0.46	2.18E-01	-0.76	3.47E-02	-1.11	1.23E-03	-0.35	3.73E-01
SNORD116-3	0.53	2.63E-01	0.13	7.96E-01	-0.87	7.97E-03	-0.40	3.41E-01	-1.40	6.89E-05	-1.00	1.46E-03
SNORD116-3	0.53	2.63E-01	0.13	7.96E-01	-0.87	7.97E-03	-0.40	3.41E-01	-1.40	6.89E-05	-1.00	1.46E-03
SNORD116-5	0.62	3.03E-01	0.16	8.04E-01	-0.78	6.52E-02	-0.47	3.99E-01	-1.40	8.83E-04	-0.93	1.66E-02
SNORD116-5	0.62	3.03E-01	0.16	8.04E-01	-0.78	6.52E-02	-0.47	3.99E-01	-1.40	8.83E-04	-0.93	1.66E-02
SNORD116-6	0.28	7.44E-01	-0.31	5.21E-01	-0.89	2.85E-02	-0.59	2.22E-01	-1.17	3.61E-03	-0.58	1.52E-01

SNORD116-8	0.38	5.32E-01	-0.03	9.69E-01	-1.13	1.54E-03	-0.40	3.81E-01	-1.51	5.89E-05	-1.11	9.91E-04
SNORD14C	0.00	9.98E-01	-0.12	8.75E-01	0.96	3.11E-02	-0.12	9.03E-01	0.96	2.94E-02	1.08	9.93E-03
SNORD33	0.37	5.28E-01	0.22	5.99E-01	-0.93	6.29E-03	-0.15	8.21E-01	-1.30	2.28E-04	-1.16	5.22E-04
SNORD49B	-0.25	6.25E-01	-1.08	9.98E-05	0.12	8.09E-01	-0.82	5.78E-03	0.37	2.26E-01	1.20	3.94E-05
SNORD82	-0.26	7.93E-01	-0.60	1.57E-01	0.72	1.15E-01	-0.35	6.07E-01	0.98	2.36E-02	1.33	1.58E-03
SOCS2	-0.29	4.59E-01	-0.89	1.49E-04	0.75	2.15E-03	-0.61	1.67E-02	1.04	5.94E-05	1.64	6.58E-08
SOD2	-0.24	8.85E-01	-0.41	5.88E-01	0.81	2.28E-01	-0.16	9.02E-01	1.06	8.81E-02	1.22	3.30E-02
SORBS2	-0.04	9.53E-01	-0.06	8.55E-01	-2.23	1.95E-10	-0.02	9.73E-01	-2.19	2.32E-10	-2.17	2.63E-10
SORT1	0.07	9.54E-01	-0.26	4.97E-01	-1.86	2.38E-06	-0.33	4.84E-01	-1.93	1.18E-06	-1.60	8.40E-06
SP110	-0.09	9.46E-01	1.07	2.76E-03	0.41	3.83E-01	1.16	5.45E-03	0.50	2.47E-01	-0.66	7.84E-02
SPAG5	-0.62	6.17E-01	-0.85	1.98E-01	0.65	4.41E-01	-0.23	8.65E-01	1.27	5.91E-02	1.51	1.71E-02
SPATA18	0.68	2.24E-02	0.54	1.57E-02	-0.36	1.81E-01	-0.14	7.48E-01	-1.03	7.87E-05	-0.90	2.55E-04
SPC25	-0.36	7.94E-01	-0.56	4.12E-01	0.70	3.33E-01	-0.20	8.79E-01	1.06	8.95E-02	1.26	2.92E-02
SPICE1	0.09	9.06E-01	0.85	2.98E-04	-0.27	3.62E-01	0.76	3.43E-03	-0.35	1.74E-01	-1.11	1.71E-05
SPP1	-0.12	9.61E-01	0.89	2.13E-01	-0.77	3.76E-01	1.00	2.46E-01	-0.65	4.74E-01	-1.65	1.38E-02
SPTLC3	0.28	8.68E-01	-0.67	3.06E-01	0.81	2.52E-01	-0.95	1.99E-01	0.53	5.15E-01	1.48	1.19E-02
SQLE	1.10	1.02E-02	1.42	7.32E-05	0.55	1.49E-01	0.32	5.51E-01	-0.55	1.36E-01	-0.87	9.22E-03
SRGN	-0.12	9.64E-01	-0.40	7.22E-01	-4.41	3.72E-06	-0.28	8.64E-01	-4.29	4.22E-06	-4.01	6.81E-06
SRPX2	0.42	5.22E-01	0.13	8.21E-01	-0.74	5.56E-02	-0.29	6.30E-01	-1.16	2.36E-03	-0.87	1.47E-02
ST8SIA1	0.91	3.44E-01	1.74	3.05E-03	0.49	5.71E-01	0.83	2.94E-01	-0.41	6.51E-01	-1.25	3.86E-02
ST8SIA1	1.11	1.33E-01	1.83	4.91E-04	0.91	1.14E-01	0.72	2.89E-01	-0.19	8.37E-01	-0.92	8.49E-02
STAMBPL1	-0.09	9.30E-01	-0.06	9.22E-01	1.68	1.86E-05	0.04	9.70E-01	1.77	6.02E-06	1.74	5.40E-06
STARD4	1.15	1.75E-01	1.67	3.58E-03	-0.36	7.04E-01	0.53	5.80E-01	-1.51	1.48E-02	-2.04	8.18E-04
STAT4	0.36	7.40E-01	0.29	6.67E-01	-1.37	8.17E-03	-0.07	9.60E-01	-1.72	9.42E-04	-1.66	8.78E-04
STC1	-1.49	1.17E-02	-1.45	1.58E-03	-1.07	3.12E-02	0.04	9.75E-01	0.42	5.09E-01	0.38	5.43E-01
STC2	-0.96	1.57E-01	-1.03	2.79E-02	-1.40	6.37E-03	-0.06	9.62E-01	-0.44	5.10E-01	-0.37	5.74E-01
STEAP1	-0.44	7.46E-01	0.17	8.72E-01	1.26	5.26E-02	0.61	5.13E-01	1.70	7.54E-03	1.09	7.68E-02
STEAP2	-0.17	9.27E-01	-0.32	6.89E-01	1.52	1.20E-02	-0.16	9.08E-01	1.68	4.81E-03	1.84	1.46E-03
STEAP4	-0.05	9.95E-01	-2.00	1.55E-01	2.54	8.73E-02	-1.95	2.71E-01	2.59	7.71E-02	4.54	1.12E-03
STIL	-0.29	8.19E-01	-0.05	9.59E-01	0.81	1.64E-01	0.24	8.14E-01	1.10	4.14E-02	0.86	1.04E-01
STK32B	0.22	9.01E-01	1.10	4.27E-02	0.21	8.43E-01	0.88	2.04E-01	-0.01	9.98E-01	-0.89	1.29E-01
STMN1	-0.85	2.62E-01	-1.71	6.57E-04	0.02	9.88E-01	-0.85	1.59E-01	0.87	1.11E-01	1.72	8.11E-04
STYXL1	-0.21	7.13E-01	-0.69	6.63E-03	-1.02	5.20E-04	-0.48	1.26E-01	-0.81	3.63E-03	-0.33	2.82E-01
SVIL	0.37	8.05E-01	-0.32	7.21E-01	1.14	8.74E-02	-0.69	4.34E-01	0.77	2.97E-01	1.46	1.66E-02

SYDE2	0.31	5.97E-01	0.26	4.95E-01	-1.19	4.55E-04	-0.05	9.45E-01	-1.50	2.18E-05	-1.45	2.28E-05
SYNE2	-0.42	2.43E-01	0.12	7.33E-01	1.01	2.14E-04	0.54	4.67E-02	1.43	2.07E-06	0.89	4.27E-04
SYNPO2	0.32	5.37E-01	0.07	8.84E-01	-0.85	4.18E-03	-0.24	5.85E-01	-1.17	1.59E-04	-0.93	1.11E-03
SYT14	-1.13	2.58E-01	-1.35	3.65E-02	-0.49	6.22E-01	-0.21	8.86E-01	0.64	4.69E-01	0.86	2.49E-01
TACC3	-0.42	7.53E-01	-0.79	2.05E-01	0.58	4.62E-01	-0.37	7.33E-01	1.00	1.21E-01	1.37	1.97E-02
TAGLN	0.14	9.51E-01	-0.29	7.77E-01	-2.21	2.03E-03	-0.44	7.12E-01	-2.35	9.32E-04	-1.92	3.88E-03
TANC1	0.39	6.91E-01	0.53	3.10E-01	-0.55	3.59E-01	0.14	8.99E-01	-0.93	6.00E-02	-1.07	2.12E-02
TBC1D8	-0.06	9.61E-01	-0.30	5.03E-01	0.76	4.67E-02	-0.24	7.10E-01	0.82	2.83E-02	1.06	3.24E-03
TBC1D8B	0.00	9.98E-01	0.50	5.10E-02	-0.50	7.82E-02	0.50	1.04E-01	-0.50	7.58E-02	-1.00	2.83E-04
TBL1X	-0.33	3.63E-01	-0.83	2.98E-04	0.36	1.54E-01	-0.50	4.88E-02	0.69	3.48E-03	1.19	5.58E-06
TCP11L2	-0.49	6.81E-01	-1.62	3.63E-03	0.05	9.71E-01	-1.13	9.16E-02	0.54	4.88E-01	1.67	3.76E-03
TES	0.29	6.57E-01	0.50	1.39E-01	-0.66	6.41E-02	0.20	7.33E-01	-0.95	5.91E-03	-1.16	6.89E-04
TEX2	0.23	4.57E-01	1.11	1.14E-06	-0.19	4.45E-01	0.87	1.22E-04	-0.42	2.84E-02	-1.30	9.36E-08
TFPI	-0.03	9.86E-01	-0.25	6.89E-01	-1.95	1.17E-04	-0.22	7.99E-01	-1.92	1.10E-04	-1.70	2.71E-04
TFRC	-1.45	1.22E-03	-0.76	2.96E-02	-0.49	2.52E-01	0.69	1.10E-01	0.96	1.17E-02	0.27	5.95E-01
TGFB2	0.50	4.54E-01	-0.07	9.24E-01	0.98	1.85E-02	-0.57	2.58E-01	0.48	3.21E-01	1.05	7.42E-03
TGFB3	0.71	2.04E-01	0.84	2.31E-02	1.05	9.82E-03	0.13	8.80E-01	0.34	5.26E-01	0.21	7.26E-01
TGM2	-0.32	8.63E-01	0.35	7.14E-01	-1.87	8.28E-03	0.67	5.09E-01	-1.55	2.61E-02	-2.22	1.08E-03
THBS4	0.05	9.89E-01	0.30	8.11E-01	4.04	2.16E-05	0.26	8.85E-01	3.99	1.65E-05	3.73	2.54E-05
THRB	0.21	7.65E-01	0.18	6.91E-01	1.80	3.72E-06	-0.04	9.65E-01	1.58	1.36E-05	1.62	7.09E-06
TIGD2	-0.63	2.02E-02	-0.73	5.77E-04	-1.14	9.88E-06	-0.10	8.11E-01	-0.51	2.23E-02	-0.41	5.72E-02
TIPARP	1.11	3.40E-03	1.09	4.00E-04	0.23	6.00E-01	-0.02	9.82E-01	-0.87	5.99E-03	-0.85	5.03E-03
TJP2	-0.10	8.95E-01	0.25	3.59E-01	1.15	4.97E-05	0.35	2.62E-01	1.25	1.25E-05	0.89	4.09E-04
TLCD1	0.43	1.04E-01	1.13	1.56E-06	0.07	8.45E-01	0.70	1.80E-03	-0.36	8.27E-02	-1.06	3.50E-06
TLR3	0.45	5.94E-01	1.00	1.84E-02	0.36	5.76E-01	0.55	3.66E-01	-0.10	9.16E-01	-0.65	1.72E-01
TM4SF1	0.17	8.37E-01	-0.45	1.99E-01	0.92	8.97E-03	-0.62	1.15E-01	0.75	3.22E-02	1.37	1.25E-04
TMCO7	0.29	7.35E-01	0.72	5.14E-02	-0.94	2.02E-02	0.44	4.17E-01	-1.22	2.41E-03	-1.66	6.10E-05
TMEM106A	-0.05	9.61E-01	0.54	9.58E-02	-0.47	2.09E-01	0.59	1.23E-01	-0.42	2.78E-01	-1.01	1.99E-03
TMEM117	0.45	2.39E-01	1.15	4.22E-05	-0.03	9.61E-01	0.70	1.58E-02	-0.48	8.17E-02	-1.18	3.47E-05
TMEM154	0.09	9.44E-01	-0.23	6.45E-01	1.33	6.65E-04	-0.31	5.88E-01	1.24	1.11E-03	1.56	6.10E-05
TMEM195	0.09	8.98E-01	0.02	9.57E-01	1.97	8.15E-09	-0.07	8.98E-01	1.88	1.54E-08	1.95	6.12E-09
TMEM30B	0.28	7.79E-01	0.39	4.43E-01	1.03	2.19E-02	0.11	9.14E-01	0.75	1.03E-01	0.64	1.54E-01
TMEM45A	-0.17	7.02E-01	0.01	9.72E-01	-0.99	5.70E-05	0.19	5.85E-01	-0.82	3.80E-04	-1.00	2.21E-05
TMPO	-0.04	9.82E-01	-0.38	4.78E-01	0.67	1.82E-01	-0.34	6.43E-01	0.71	1.45E-01	1.05	1.61E-02

TNC	0.32	6.80E-01	1.06	3.04E-03	0.87	2.57E-02	0.75	8.07E-02	0.56	1.81E-01	-0.19	7.46E-01
TNFAIP6	0.19	9.24E-01	2.68	5.39E-05	1.69	9.61E-03	2.49	6.98E-04	1.50	2.01E-02	-0.99	1.23E-01
TNFRSF10D	-0.03	9.87E-01	-1.13	1.28E-02	-0.40	5.55E-01	-1.10	3.97E-02	-0.37	5.96E-01	0.73	1.43E-01
TNFRSF19	0.62	9.79E-02	-0.10	8.19E-01	-0.81	5.65E-03	-0.72	1.88E-02	-1.43	1.02E-05	-0.71	9.48E-03
TNIK	-0.66	4.72E-01	-0.45	4.88E-01	-1.54	6.40E-03	0.21	8.52E-01	-0.88	1.35E-01	-1.09	4.05E-02
TNS3	-0.50	2.77E-01	-0.34	2.98E-01	-1.21	2.99E-04	0.15	7.88E-01	-0.71	2.30E-02	-0.87	3.89E-03
TOB2	-0.21	8.37E-01	-0.64	1.04E-01	0.62	1.65E-01	-0.43	4.43E-01	0.82	4.64E-02	1.25	1.55E-03
TOB2	-0.21	8.37E-01	-0.64	1.04E-01	0.62	1.65E-01	-0.43	4.43E-01	0.82	4.64E-02	1.25	1.55E-03
TOP2A	-1.32	2.65E-01	-2.42	1.61E-03	0.38	7.82E-01	-1.10	2.80E-01	1.70	3.82E-02	2.80	5.47E-04
TPK1	0.53	3.00E-01	1.19	3.96E-04	0.51	1.77E-01	0.66	8.66E-02	-0.02	9.83E-01	-0.68	4.06E-02
TPST2	0.12	9.09E-01	0.51	1.17E-01	-0.71	3.84E-02	0.40	3.77E-01	-0.83	1.42E-02	-1.23	3.00E-04
TRAF5	0.67	2.14E-01	0.50	1.98E-01	-0.44	3.36E-01	-0.18	8.07E-01	-1.12	3.84E-03	-0.94	1.03E-02
TRIM16	-0.04	9.65E-01	0.60	8.77E-03	-0.45	8.40E-02	0.63	1.68E-02	-0.41	1.15E-01	-1.04	5.59E-05
TRIM16L	0.12	8.58E-01	0.80	1.39E-03	-0.41	1.58E-01	0.68	1.74E-02	-0.53	4.82E-02	-1.21	2.21E-05
TRIP13	-0.80	5.75E-01	-0.85	3.13E-01	0.72	4.86E-01	-0.05	9.85E-01	1.52	5.92E-02	1.57	3.88E-02
TRPC4	-0.60	1.50E-01	-0.69	1.34E-02	-1.82	1.15E-06	-0.10	8.80E-01	-1.22	1.71E-04	-1.12	2.69E-04
TRPS1	0.08	9.58E-01	0.06	9.41E-01	1.01	2.67E-02	-0.02	9.87E-01	0.93	3.93E-02	0.95	2.60E-02
TSHZ2	0.15	9.56E-01	-0.49	6.11E-01	1.06	2.13E-01	-0.64	5.85E-01	0.91	2.99E-01	1.55	3.25E-02
TSPAN11	-0.05	9.81E-01	-0.76	1.54E-01	0.71	2.45E-01	-0.71	2.97E-01	0.76	1.98E-01	1.46	4.54E-03
TSPAN12	0.46	3.97E-01	1.12	7.44E-04	-0.20	7.21E-01	0.66	9.13E-02	-0.66	6.41E-02	-1.32	2.03E-04
TSPAN13	0.17	9.18E-01	0.88	7.94E-02	-0.49	4.71E-01	0.72	2.79E-01	-0.66	2.74E-01	-1.38	6.61E-03
TSPAN18	-0.05	9.87E-01	-1.49	5.82E-02	-3.05	5.65E-04	-1.43	1.35E-01	-3.00	5.37E-04	-1.57	5.16E-02
TSPAN2	0.43	8.52E-01	0.79	4.36E-01	-1.34	1.71E-01	0.37	8.35E-01	-1.76	5.15E-02	-2.13	1.22E-02
TSPAN5	0.26	6.28E-01	0.53	4.72E-02	1.09	3.58E-04	0.27	5.29E-01	0.83	3.98E-03	0.56	4.07E-02
TTK	-0.89	5.93E-01	-1.10	2.31E-01	0.84	4.70E-01	-0.21	9.18E-01	1.73	5.92E-02	1.95	2.35E-02
TUBB2A	0.56	2.52E-01	1.13	5.15E-04	0.71	4.23E-02	0.57	1.42E-01	0.14	7.99E-01	-0.43	2.37E-01
TUBB2A	0.56	2.52E-01	1.13	5.15E-04	0.71	4.23E-02	0.57	1.42E-01	0.14	7.99E-01	-0.43	2.37E-01
TUFT1	0.33	5.06E-01	0.53	5.28E-02	-0.85	4.80E-03	0.20	6.83E-01	-1.18	1.59E-04	-1.38	1.45E-05
TYMS	-0.71	5.31E-01	-1.54	1.01E-02	0.34	7.47E-01	-0.83	3.23E-01	1.05	1.25E-01	1.88	2.76E-03
UBE2C	-0.28	8.24E-01	-0.54	3.40E-01	0.75	2.05E-01	-0.26	7.88E-01	1.03	5.53E-02	1.29	1.04E-02
UCHL1	0.01	9.95E-01	0.39	3.79E-01	-0.68	1.12E-01	0.38	5.30E-01	-0.70	9.83E-02	-1.07	5.66E-03
UCK2	0.32	4.28E-01	1.11	3.09E-05	0.36	1.98E-01	0.79	3.96E-03	0.04	9.42E-01	-0.75	2.23E-03
UCP2	0.16	8.81E-01	-0.30	5.06E-01	0.82	3.67E-02	-0.46	3.54E-01	0.66	1.00E-01	1.12	2.49E-03
ULBP1	-0.21	9.07E-01	-0.43	5.64E-01	1.93	1.92E-03	-0.21	8.61E-01	2.14	5.56E-04	2.35	1.29E-04

UNC5B	0.20	8.82E-01	0.39	4.82E-01	-0.67	1.94E-01	0.19	8.39E-01	-0.87	7.04E-02	-1.06	1.76E-02
UQCR11	-0.06	9.55E-01	-0.36	2.24E-01	-1.07	4.78E-04	-0.30	4.60E-01	-1.01	6.62E-04	-0.71	9.37E-03
USP18	0.34	6.60E-01	1.61	8.09E-05	-0.03	9.75E-01	1.27	3.62E-03	-0.37	4.75E-01	-1.64	8.39E-05
USP2	-0.06	9.45E-01	0.01	9.82E-01	0.96	1.22E-03	0.08	9.02E-01	1.03	4.99E-04	0.95	7.22E-04
USP53	0.14	9.30E-01	0.24	7.48E-01	-1.16	2.49E-02	0.10	9.37E-01	-1.30	1.10E-02	-1.40	4.29E-03
USP9Y	-0.02	9.95E-01	-0.02	9.89E-01	2.88	3.24E-05	0.00	1.00E+00	2.90	2.13E-05	2.90	1.43E-05
UTY	0.12	9.18E-01	-0.07	9.21E-01	1.46	3.48E-04	-0.19	7.90E-01	1.33	6.75E-04	1.52	1.06E-04
VCAM1	0.54	7.83E-01	0.83	3.85E-01	-3.92	5.05E-05	0.29	8.74E-01	-4.46	7.02E-06	-4.75	2.30E-06
VDR	0.05	9.52E-01	0.51	3.17E-02	-0.85	1.49E-03	0.46	1.16E-01	-0.91	6.58E-04	-1.37	3.50E-06
VGLL3	0.15	8.84E-01	-0.08	8.96E-01	-1.64	4.21E-05	-0.23	7.11E-01	-1.79	1.02E-05	-1.56	3.64E-05
VRK2	0.39	2.88E-01	0.48	4.04E-02	1.03	1.58E-04	0.09	8.68E-01	0.64	1.13E-02	0.55	2.11E-02
WDR76	-0.85	4.42E-01	-1.33	3.46E-02	0.69	4.22E-01	-0.49	6.60E-01	1.53	2.44E-02	2.02	2.14E-03
WEE1	-0.72	4.45E-01	-1.33	1.28E-02	0.30	7.49E-01	-0.61	4.52E-01	1.02	8.95E-02	1.63	3.52E-03
WEE1	-0.39	7.33E-01	-0.84	1.10E-01	0.50	4.73E-01	-0.45	5.93E-01	0.89	1.16E-01	1.34	9.44E-03
WNT5A	-0.25	8.67E-01	-0.73	1.93E-01	-1.38	1.54E-02	-0.48	5.80E-01	-1.13	4.69E-02	-0.65	2.96E-01
XAF1	0.26	8.04E-01	1.71	1.54E-04	0.42	4.77E-01	1.45	3.43E-03	0.16	8.44E-01	-1.29	3.20E-03
XPNPEP1	-0.08	9.20E-01	0.25	3.46E-01	-0.79	1.92E-03	0.33	2.89E-01	-0.71	3.85E-03	-1.04	5.34E-05
ZADH2	-0.06	9.44E-01	-0.23	4.44E-01	0.95	5.45E-04	-0.17	7.03E-01	1.01	2.25E-04	1.18	2.23E-05
ZC3H12C	0.08	9.35E-01	-0.15	7.37E-01	1.68	6.23E-06	-0.23	6.49E-01	1.60	9.20E-06	1.83	1.07E-06
ZDHHC2	0.05	9.49E-01	-0.49	1.51E-02	0.55	1.32E-02	-0.54	2.19E-02	0.51	2.19E-02	1.05	1.31E-05
ZEB1	0.33	5.83E-01	-0.12	8.08E-01	0.93	5.98E-03	-0.46	2.69E-01	0.60	7.89E-02	1.06	1.14E-03
ZFP36	0.43	6.46E-01	-0.06	9.42E-01	0.94	6.59E-02	-0.50	4.87E-01	0.51	4.03E-01	1.01	3.37E-02
ZFPM2	0.09	9.34E-01	-0.10	8.72E-01	2.32	3.68E-07	-0.19	7.70E-01	2.23	5.42E-07	2.42	1.01E-07
ZFY	0.08	9.50E-01	0.06	9.24E-01	1.58	7.67E-05	-0.02	9.87E-01	1.50	1.09E-04	1.52	5.79E-05
ZNF267	0.28	7.24E-01	0.79	2.44E-02	1.02	8.27E-03	0.51	2.84E-01	0.74	5.48E-02	0.23	6.69E-01
ZNF385D	0.29	9.41E-01	0.34	8.64E-01	2.99	1.34E-02	0.05	9.88E-01	2.70	2.35E-02	2.65	1.94E-02
ZNF415	0.00	9.98E-01	-0.41	2.20E-01	-1.03	2.19E-03	-0.40	3.44E-01	-1.03	1.95E-03	-0.63	4.46E-02
ZNF474	0.19	6.12E-01	1.28	4.85E-07	-0.12	6.83E-01	1.09	1.76E-05	-0.32	1.42E-01	-1.40	6.82E-08
ZNF521	0.23	9.50E-01	0.00	9.99E-01	2.14	5.13E-02	-0.23	9.27E-01	1.91	8.29E-02	2.14	3.60E-02
ZNF730	-0.10	9.57E-01	0.06	9.55E-01	1.46	8.44E-03	0.16	8.95E-01	1.56	4.31E-03	1.41	6.99E-03
