# Supplementary material for: Identification of Pathways Mediating Growth Differentiation Factor5-Induced Tenogenic Differentiation in Human Bone Marrow Stromal Cells
Source: PLoS One. 2015 Nov 3;10(11):e0140869. doi: 10.1371/journal.pone.0140869 (PMC4631504; doi:10.1371/journal.pone.0140869)
Supplement: S8 Table — (PDF) [file pone.0140869.s012.pdf]

S8 Table. Pathways regulated by GDF5-induced tenogensis in hMSC.

Pathway regulated in hMSC at day 4 of 100 ng/ml GDF5 induction (Total= 8 pathways)

**Pathway name**	**Genes involved**	***p*-value**
Cholesterol biosynthesis	FDPS↑, SC4MOL↑, ERG1↑	3.603E-04
Glycolysis and gluconeogenesis p.3 / Human version	ENO↓, ENO2↓	6.822E-04
Glycolysis and gluconeogenesis p.3	ENO↓, ENO2↓	6.822E-04
Immune response_TREM1 signalling pathway	PI3K reg class IA↑, CCL2↑	4.092E-03
Immune response_IL-17 signalling pathways	PI3K reg class IA↑, CCL2↑	4.229E-03
Glycolysis and gluconeogenesis (short map)	ENO2↓	5.096E-03
Development_VEGF signaling via VEGFR2 - generic cascades	PI3K reg class IA↑, CCL2↑	8.143E-03
Regulation of lipid metabolism_Insulin regulation of fatty acid methabolism	PI3K reg class IA↑, FADS2↑	9.105E-03

Pathway regulated in hMSC at day 10 of 100 ng/ml GDF5 induction (Total= 21 pathways)

**Pathway name**	**Genes involved**	***p*-value**
Cell cycle_Role of APC in cell cycle regulation	PLK1↓, BUBR1↓, Aurora-A↓, Cyclin A↓, Cyclin B↓, Nek2A↓, Securin↓, APC↓, CDC18L(CDC6)↑	3.540E-10
Cell cycle_The metaphase checkpoint	Survivin↓, Aurora-A↓, Nek2A↓, PLK1↓, BUBR1↓, AF15q14↓, CENP-F↓	2.731E-08
Cell cycle_Chromosome condensation in prometaphase	Cyclin A↓, Cyclin B↓, CAP-G↓, Aurora-A↓, CAP-G/G2↓, Condensin↓, TOP2↓	3.203E-07
Cholesterol Biosynthesis	IDI1↑, HMGCS1↑, HMDH↑, ERG1↑, FDPS↑, SC4MOL↑, SC5D↑	2.325E-04
Cell cycle_Initiation of mitosis	PLK1↓, Wee1↓, FOXM1↓, Cyclin B2↓	3.811E-04
Immune response_Antiviral actions of Interferons	OAS1↑, OAS2↑, OAS3↑, 2′-5′-oligoadenylate synthetase↑, MxA↑	7.921E-04
Cell cycle_Role of Nek in cell cycle regulation	PI3K reg class IA↑, Nek2A↓, tubulin beta↑, Aurora-A↓	1.004E-03
Cell cycle_Spindle assembly and chromosome separation	Anaphase-promoting complex (APC)↓, Nek2A↓, Securin↓, Aurora-A↓	1.130E-03
Development_Angiopoietin - Tie2 signaling	Angiopoietin 3↑, PI3K reg class IA↑, Grb14↑, Survivin↓	1.415E-03
Development_TGF-beta-dependent induction of EMT via SMADs	Endothelin-1↓, SMAD3↓, ID2↓, E-cadherin↑	1.415E-03
Cytoskeleton remodeling_Keratin filaments	Tubulin beta↑, Keratin 14↑, Keratin 16↑, Kereatin 17↑	1.575E-03
Cell cycle_Regulation of G1/S transition (part 1)	Anaphase-promoting complex (APC) ↓, PP2A regulatory↑, SMAD3↓, Cyclin A↓, CDK6↑	1.931E-03
Cell cycle_Cell cycle (generic schema)	CDK6↑, CyclinA↓, Cyclin B↓	3.038E-03

S8 Table continued

Immune response_NF-AT signaling and leukocyte interactions	NF-AT1↑, cPAL2↑, Ca(II) channel↑, Eotaxin↑	3.914E-03
Immune response _CCR3 signaling in eosinophils	Eotaxin↑, Eotaxin 3↑, Rac2↓, MyHC↑, Myosin II ↑	4.553E-03
Arachidonic acid production	FADS1↑, PA24A↑, cPLA2↑, PLA2↑	5.290E-03
DNA damage_ATM / ATR regulation of G2 / M checkpoint	Wee1↓, Cyclin A↓, Cyclin B↓	5.643E-03
Cell cycle_Transition and termination of DNA replication	Cyclin A↓, TOP1 alpha↓, TOP2↓	6.966E-03
Immune response_Innate immune response to RNA viral infection	TLR3↓, RIG-I↓, MDA-5↓	6.966E-03
Cell cycle_Role of SCF complex in cell cycle regulation	APC↓, PLK1↓, Wee1↓	7.690E-03
Immune response_TREM1 signaling pathway	PI3K reg class IA↑, CCL2↑, NFATC2↑	9.488E-03

Pathway regulated in tenocytes (Total= 18 pathways)

**Pathway name**	**Genes involved**	***p*-value**
Immune response_Alternative complement pathway	C3↑, C3a↑, iC3b↑, C3c↑, C3dg↑, C3b↑, C5 convertase (C3bBb)↑, Clusterin↑	1.129E-07
Immune response_Lectin induced complement pathway	C3↑, C3a↑, C3b↑, C3c↑, C3dg↑, iC3b↑, DAF↑, C5 convertase (C2aC4bC3b)↑	7.265E-07
Immune response_Classical complement pathway	C3↑, C3a↑, C3b↑, C3c↑, C3dg↑, iC3b↑, DAF↑, C5 convertase (C2aC4bC3b)↑	1.166E-06
Development_Regulation of epithelial-to-mesenchymal transition (EMT)	HGF↓, WNT↓, Jagged1↓, TGF-beta 3↑, Endothelin-1↓, ENDRA↓, ACTA2 ↓	5.553E-05
Immune response_MIF-mediated glucocorticoid regulation	I-kB↑, NFKBIA↑, IL-8↑, VCAM↓	3.282E-04
Development_TGF-beta-dependent induction of EMT via SMADs	Endothelin-1↓ , TGF-beta↑, TGF-beta 3↑, Jagged1↓	2.010E-03
Transcription_Role of AP-1 in regulation of cellular metabolism	ITGA2↑, TSG-6↑, GCL cat↑, GCL reg↑	2.733E-03
Apoptosis and survival_Lymphotoxin-beta receptor signaling	IL-8↑, I-kB↑, VCAM↓, SDF-1↓	3.951E-03
Cell adhesion_Ephrin signaling	Ephrin-B↓, Ephrin-B receptors↑, Ephrin-B receptor 1↑, Ephrin-A receptors↓	5.075E-03
Immune response_PGE2 signaling in immune response	PGE2R2↑, PGES↓, IL-8↑, HGF↓	5.075E-03
Development_TGF-beta-dependent induction of EMT via RhoA, PI3K and ILK.	TGF-beta 3↑, ACTA2↓, I-kB↑, Actin↓	5.492E-03
Immune response_Histamine H1 receptor signaling in immune response	IL-8↑, VCAM1↓, NFKBIA↑, I-kB↑	6.394E-03
Muscle contraction_Relaxin signaling pathway	Endothelin-1↓, PDE4D↑, NFKBIA↑, I-kB↑	6.394E-03

S8 Table continued

Development_PEDF signaling	PEDF (serpinF1)↑, NFKBIA↑, c-IAP2↑, BDNF↓	6.881E-03
Development_Cross-talk between VEGF and Angiopoietin 1 signaling pathways	VCAM1↓, I-kB↑, Angiopoietin 1↓	7.347E-03
Development_S1P2 and S1P3 receptors in cell proliferation and differentiation	ACTA2↓, Transgelin↓, Actin↓	7.347E-03
Immune response_HMGB1/RAGE signaling pathway	VCAM1↓, I-kB↑, NFKBIA↑, IL-8↑	9.068E-03
Muscle contraction_GPCRs in the regulation of smooth muscle tone	Endothelin-1↓, ENDRA↓, PGE2R2↑, MRLC↓, Myosin II↓	9.278E-03

Pathway regulated in GDF5-induced hMSC (day 4 and day 10) (Total= 3 pathways)

**Pathway name**	**Change**	***p*-value**		
		**GDF5-induced hMSC (Day 4)**	**GDF5-induced hMSC (Day 10)**	**Day 10 vs day 4**
Development_Angiopoietin - Tie2 signaling	+	5.622E-2	1.415E-3	8.316E-2
Development_TGF-beta-dependent induction of EMT via SMADs	+	5.622E-2	1.415E-3	8.316E-2
Immune response_IL-13 signaling via JAK-STAT	+	7.019E-2	2.393E-2	5.175E-3

Pathway regulated in GDF5-induced hMSC (day 4 and day 10) and tenocytes (Total= 11 pathways)

**Pathway name**	**Change**	***p*-value**		
		**GDF5-induced hMSC (Day 4)**	**GDF5-induced hMSC (Day 10)**	**Tenocytes**
Glycolysis and gluconeogenesis p.3 / Human version	-	6.822E-4	4.463E-2	5.296E-2
Glycolysis and gluconeogenesis p.3	-	6.822E-4	4.463E-2	5.296E-2
Cell cycle_Role of Nek in cell cycle regulation	+	5.152E-2	1.004E-3	8.790E-2
Development_Angiopoietin - Tie2 signaling	+	5.622E-2	1.415E-3	1.024E-1
Development_TGF-beta-dependent induction of EMT via SMADs	+	5.622E-2	1.415E-3	2.010E-3
Immune response_TREM1 signaling pathway	+	4.092E-3	9.488E-3	1.313E-2
Immune response_IL-17 signaling pathways	+	4.229E-3	2.078E-1	1.390E-2
Glycolysis and gluconeogenesis (short map)	-	5.096E-3	6.630E-2	2.738E-1
Development_PEDF signaling	+	7.787E-2	5.030E-1	6.881E-3
Development_VEGF signaling via VEGFR2 - generic cascades	+	8.143E-3	1.155E-1	3.765E-1

S8 Table continued

Regulation of lipid metabolism_Insulin regulation of fatty acid methabolism	+	9.105E-3	3.592E-1	7.538E-1

Pathway regulated in tenocytes compared to GDF5-induced hMSC (day 4 and day 10) (Total= 41 pathways)

Pathway name	Change	*p*-value		
		**Tenocytes vs control hMSC**	**Tenocytes vs GDF5-induced hMSC (Day4)**	**Tenocytes vs GDF5-induced hMSC (Day 10)**
Cell cycle_Chromosome condensation in prometaphase	+	2.806E-1	1.132E-7	2.303E-12
Cell cycle_Role of APC in cell cycle regulation	+	3.948E-1	1.365E-1	1.134E-8
Cell cycle_Initiation of mitosis	+	5.699E-2	8.134E-6	1.519E-8
Immune response_Alternative complement pathway	+	1.129E-7	1.866E-1	7.618E-4
Immune response_Lectin induced complement pathway	+	7.265E-7	2.614E-1	1.259E-2
Immune response_Classical complement pathway	+	1.166E-6	2.840E-1	1.602E-2
Development_TGF-beta-dependent induction of EMT via SMADs	+	2.010E-3	1.576E-1	4.825E-6
Development_Regulation of epithelial-to-mesenchymal transition (EMT)	-	5.553E-5	2.906E-4	1.190E-5
Immune response_MIF-mediated glucocorticoid regulation	+	3.282E-4	6.401E-5	2.618E-5
Immune response_Histamine H1 receptor signaling in immune response	+	6.394E-3	4.521E-5	2.313E-3
Cell adhesion_ECM remodeling	+	4.671E-2	7.675E-5	6.418E-4
Cell cycle_Role of Nek in cell cycle regulation	+	8.790E-2	1.365E-1	2.501E-4
DNA damage_ATM/ATR regulation of G1/S checkpoint	+	3.948E-1	1.365E-1	2.501E-4
Cell cycle_Sister chromatid cohesion	+	2.918E-1	9.471E-3	3.251E-4
GTP metabolism	-	5.128E-2	7.298E-4	6.803E-2
Transcription_Role of AP-1 in regulation of cellular metabolism	+	2.733E-3	9.389E-4	2.968E-1
Cell cycle_Role of SCF complex in cell cycle regulation	+	3.656E-1	4.481E-1	1.245E-3
Apoptosis and survival_Lymphotoxin-beta receptor signaling	+	3.951E-3	1.491E-3	1.179E-1
Transcription_Role of VDR in regulation of genes involved in osteoporosis	-	6.889E-2	3.513E-1	1.683E-3
Immune response_IL-1 signaling pathway	+	3.059E-2	1.842E-3	1.306E-1
Development_TGF-beta-dependent induction of EMT via RhoA, PI3K and ILK.	+	5.492E-3	6.578E-2	1.851E-3

S8 Table continued

Development_NOTCH1-mediated pathway for NF-KB activity modulation	+	1.546E-2	4.689E-3	2.597E-3
Immune response_Gastrin in inflammatory response	+	2.216E-2	2.639E-3	1.348E-1
Immune response_HMGB1/RAGE signaling pathway	+	9.068E-3	4.206E-3	1.729E-2
Cell adhesion_Cell-matrix glycoconjugates	+	1.174E-1	4.104E-2	4.270E-3
Airway smooth muscle contraction in asthma	-	5.606E-2	1.041E-1	5.062E-3
Cell adhesion_Ephrin signaling	+	5.075E-3	1.269E-2	3.898E-2
Immune response_PGE2 signaling in immune response	+	5.075E-3	1.269E-2	1.372E-1
Immune response_Role of PKR in stress-induced antiviral cell response	+	5.853E-2	5.753E-3	2.229E-1
Cell adhesion_Tight junctions	+	1.803E-2	5.772E-3	2.754E-1
Cell adhesion_Cadherin-mediated cell adhesion	+	6.113E-2	1.507E-2	5.953E-3
Muscle contraction_Delta-type opioid receptor in smooth muscle contraction	-	6.113E-2	1.507E-2	5.953E-3
Development_S1P2 and S1P3 receptors in cell proliferation and differentiation	-	7.347E-3	1.507E-2	5.953E-3
Muscle contraction_Relaxin signaling pathway	+	6.394E-3	1.582E-2	1.574E-1
Development_PEDF signaling	+	6.881E-3	6.341E-1	5.080E-2
Immune response_IL-17 signaling pathways	+	1.390E-2	7.148E-3	2.804E-2
Development_Cross-talk between VEGF and Angiopoietin 1 signaling pathways	+	7.347E-3	1.507E-2	3.704E-2
Cell adhesion_PLAU signaling	+	1.226E-1	7.691E-3	2.455E-2
Transport_Macropinocytosis regulation by growth factors	+	7.437E-2	3.841E-2	8.979E-3
Muscle contraction_GPCRs in the regulation of smooth muscle tone	-	9.278E-3	2.627E-2	9.435E-3
Apoptosis and survival_Granzyme A signaling	+	3.755E-1	2.220E-2	9.983E-3
Cell cycle_Chromosome condensation in prometaphase	+	2.806E-1	1.132E-7	2.303E-12

↑ Up-regulated; ↓ Down-regulated
